# 4,7-Disubstituted 7*H*-Pyrrolo[2,3-d]pyrimidines and Their Analogs as Antiviral Agents against Zika Virus

**DOI:** 10.3390/molecules26133779

**Published:** 2021-06-22

**Authors:** Ruben Soto-Acosta, Eunkyung Jung, Li Qiu, Daniel J. Wilson, Robert J. Geraghty, Liqiang Chen

**Affiliations:** Center for Drug Design, College of Pharmacy, University of Minnesota, Minneapolis, MN 55455, USA; rsotoaco@umn.edu (R.S.-A.); ejung@umn.edu (E.J.); liqiu2017@gmail.com (L.Q.); wils0125@umn.edu (D.J.W.)

**Keywords:** Zika virus, dengue virus, flavivirus, antiviral agents

## Abstract

Discovery of compound **1** as a Zika virus (ZIKV) inhibitor has prompted us to investigate its 7*H*-pyrrolo[2,3-d]pyrimidine scaffold, revealing structural features that elicit antiviral activity. Furthermore, we have demonstrated that 9*H*-purine or 1*H*-pyrazolo[3,4-d]pyrimidine can serve as an alternative core structure. Overall, we have identified 4,7-disubstituted 7*H*-pyrrolo[2,3-d]pyrimidines and their analogs including compounds **1**, **8** and **11** as promising antiviral agents against flaviviruses ZIKV and dengue virus (DENV). While the molecular target of these compounds is yet to be elucidated, 4,7-disubstituted 7*H*-pyrrolo[2,3-d]pyrimidines and their analogs are new chemotypes in the design of small molecules against flaviviruses, an important group of human pathogens.

## 1. Introduction

The *Flaviviruses* are the largest genus in the *Flaviviridae* family and include well-known human pathogens such as dengue virus (DENV), West Nile virus (WNV), yellow fever virus (YFV), Japanese encephalitis virus (JEV) and Zika virus (ZIKV) [1]. DENV infects 390 million people, among whom 100 million exhibit disease symptoms, ranging from self-limiting dengue fever to life-threatening dengue hemorrhagic fever/shock syndrome [2]. DENV remains a focus of continued antiviral research, which has been bolstered by public–private partnerships. Promising direct-acting antiviral targets include E protein, NS2B-NS3 (protease), NS4B and NS5 (RNA-dependent RNA polymerase, RdRp) [3,4,5]. Host factors that are important for viral replication or development of symptoms have also been explored [5]. While a number of promising anti-DENV agents have been tested in advanced preclinical or clinical studies, there are no specific antiviral therapeutics approved for DENV infection [6].

ZIKV has attracted public attention due to its global outbreaks and subsequent declaration as a global public health emergency by the World Health Organization (WHO) in 2016. While ZIKV infection usually causes no or mild symptoms, its association with severe neurological disorders, including microcephaly in newborn infants and Guillain-Barré syndrome in adults, has made ZIKV a global health concern [7]. The global epidemic of ZIKV and its devastating neurological complications have spurred ZIKV vaccine development and searches for small molecule anti-ZIKV therapeutics. Efforts have been devoted to targeting the ZIKV proteins, including NS2B-NS3 and NS5, facilitated by lessons learned from drug discovery in other *Flaviviridae* members, such as hepatitis C virus (HCV), DENV and WNV [8,9,10]. Moreover, phenotypic screening of compound libraries and repurposing of approved drugs have been attractive approaches to discovering anti-ZIKV drugs [11], offering opportunities to identify previously unappreciated targets, particularly host factors. However, the recent rapid decline of ZIKV infection cases has led to an end to the emergency status and ZIKV has ceased to be a world-wide public health concern. Nonetheless, the current pandemic of coronavirus disease 2019 (COVID-19) has served as a warning that reemergence of ZIKV or emergence of ZIKV-like viruses cannot be excluded, calling for a continued search for novel antiviral strategies.

**Figure 1:**
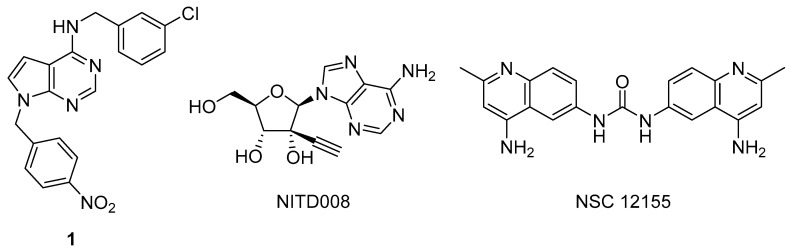
Hit compound **1** and reference compounds.

In our effort to discover antiviral agents against ZIKV, we screened our in-house compound library and identified compound **1** (Figure 1 and Table 1). Encouraged by its promising antiviral activity, we initiated a medicinal chemistry project to explore the substituents on positions 4 and 7 within compound **1**’s 7*H*-pyrrolo[2,3-d]pyrimidine scaffold, leading to analogs of excellent anti-ZIKV and -DENV activity. Here we report our structure-activity relationship (SAR) studies on compound **1** and evaluation of the resulting analogs in ZIKV and DENV.

## 2. Results and Discussion

To effectively search for ZIKV inhibitors, we constructed a luciferase-expressing ZIKV (see Supplemental Information) and established a ZIKV reporter assay. Using this assay, we screened our in-house compound library (at 10 µM) for anti-ZIKV activity along with NITD008 (Figure 1, at 1 µM) [12], a flavivirus RdRp inhibitor, and NSC 12155 (Figure 1, at 10 µM) [13], a flavivirus NS5 methyltransferase (MTase) inhibitor, as reference compounds. Gratifyingly, we identified compound **1**, which possessed anti-ZIKV activity without drastically compromised cell viability (Table 1). Compound **1**’s EC_50_ (reporter assay) and CC_50_ values were determined to be 5.25 µM and 20.0 µM, respectively, highlighting good antiviral activity and relatively low cytotoxicity. As a result, compound **1** was evaluated in a titer-reduction assay, which revealed this compound’s strong antiviral activity (93% titer reduction) at 8.5 µM. Further dose-response analysis gave rise to an EC_50_ (titer-reduction) of 5.21 µM. These remarkable results suggested that compound **1** represented a promising chemical scaffold and warranted further SAR studies.

To guide our SAR studies, we established a general testing scheme. All newly synthesized compounds were first evaluated at 10 µM in the ZIKV reporter assay and those that showed about 60% viability and about 60% inhibition were tested in the gold standard titer reduction assay at 8.5 µM or at 1.5 µM for those deemed toxic at 8.5 µM. If about 90% inhibition was reached in the titer-reduction assay, dose-response experiments were performed to determine EC_50_ (titer-reduction) values. For active compounds, EC_50_ in the reporter assay and CC_50_ were also measured.

Compound **1** contained a 7*H*-pyrrolo[2,3-d]pyrimidine core structure with substituents at positions 4 and 7. Our initial SAR study focused on ring A, which was located at position 7 (Table 1). Since the nitro group in compound **1** was an electron-withdrawing group, we first chose to reduce it to the corresponding aniline donned with an electron-donating property, giving rise to compound **2**. It showed enhanced cytotoxicity in the reporter assay, a finding that was further supported by the toxicity seen in the titer-reduction assay. Second, we prepared compound **3** with no substituents on ring A. While compound **3** was less toxic, the antiviral activity was also diminished. These two modifications suggested that an electron-withdrawing group like nitro was beneficial. As a result, we synthesized compounds **4** and **5**, in which a nitro group was placed at the ortho and meta positions, respectively. While compound **4** was less active, compound **5** exhibited antiviral activity comparable to those of compound **1** in both assays, suggesting that the meta position was another feasible modification site. After identifying these two potential modification sites, we proceeded to prepare compounds **6**–**8** and **9**–**11**. In compounds **6**–**8**, an electron-withdrawing group including trifluoromethyl, methylsufonyl, and cyano, respectively, was placed at the *para* position while in **9**–**11** an electron-withdrawing group was appended at the meta position. Evaluation of these compounds revealed that an electron-withdrawing group at either the *para* or *meta* position generally led to good anti-ZIKV activity at levels in line with that of compound **1**. Specifically, compounds **8** and **11**, in which a cyano group was used, exhibited excellent antiviral ability in the titer reduction assay. Both compounds possessed an EC_99_ value of about 13 µM even though compound **11** was slightly more toxic than compound **8** as judged by their CC_50_ values. Taken together, these results showed that introducing a simple electron-withdrawing group, preferably nitro and cyano, at the *para* or *meta* position led to excellent anti-ZIKV activity, especially in terms of titer-reducing capability. Therefore, a further structural exploration of ring A was justified.

We next investigated fused and heterocyclic rings by preparing and testing compounds **12**–**16**. Replacement of the phenyl ring in compound **1** with a 1-naphthyl or 2-naphthyl ring as seen in compound **12** or **13** led to diminished antiviral activity. Compound **14**, in which a quinolone ring was used to take advantage of its electron-deficient nature, exerted complete inhibition (EC_50_ = 0.93 µM) but showed high cytotoxicity when tested at 10 µM in the ZIKV reporter assay. To accurately assess the activity of compound **14**, it was tested at lower concentrations in the titer-reduction assay, by which the EC_90_ value was determined to be 2.28 µM. Given the fact that compound **14** has a CC_50_ value of 5.67 µM, its therapeutic window was relatively narrow. We also attempted to merge multiple structural features. For instance, a naphthyl ring was combined with a cyano group in compound **15** while a quinolone ring was used in conjunction with a chloro group in **16**. Unfortunately, these two compounds displayed negligible titer-reducing capability even though they had good activity in the reporter assay. Taken together, our exploration of fused and heterocyclic rings resulted in no significant improvement in antiviral activity.

After examination of ring A, we proceeded to study ring B at position 4 by synthesizing two groups of compounds (Table 2). Because the *para*-nitrobenzyl moiety in compound **1** had been proved to be one of the best substituents, it was retained in the first groups (compounds **17**–**24**) and variation of ring B was then explored. First, we prepared compound **17**, which had no substitution at position 4 of the 7*H*-pyrrolo[2,3-d]pyrimidine core. Compound **17**’s lack of antiviral activity clearly demonstrated that a substituent at position 4 was needed. A simple benzyl substituent in compound **18** resulted in higher toxicity when compared with compound **1**, suggesting that substitution on the phenyl was preferred. Accordingly, a chloro group at the *ortho* position was explored and the resulting compound **19** showed activity comparable to that of compound **1**. On the other hand, a *para*-chloro group in **20** gave rise to higher cytotoxicity. A combination of two chloro groups placed at different positions was also examined. Among the resultant compounds **21**–**23**, **23** exhibited high anti-ZIKV activity not only in the reporter (EC_50_ = 5.70 µM) but also in the titer-reduction assay. Notably, it achieved a three-log titer reduction (EC_99.9_ = 12.3 µM, footnote of Table 2) and had an CC_50_ value of 26.8 µM. These parameters were slightly better than those of compound **8**, one of the best inhibitors. However, use of a 1-naphthyl ring in compound **24** failed to improve antiviral activity. We also investigated another group of compounds **25**–**28**, in which a *para*-cyanobenzyl moiety as seen in compound **8** was adopted at position 7 and variation of ring B was studied. When the chloro group in compound **8** was replaced with an electron-donating methyl group, the resulting compound **25** was more toxic than compound **8**, suggesting that an electron-withdrawing group was desirable. Compounds **26**–**28**, in which chloro groups at different positions were combined, showed no significant enhancement of antiviral activity. Collectively, these structural modifications showed that a position 4 substitution was needed and electron-withdrawing group(s) on the phenyl ring were desired for anti-ZIKV property.

Besides the 7*H*-pyrrolo[2,3-d]pyrimidine scaffold, we also investigated 9*H*-purine (**29** and **30**) and 1*H*-pyrazolo[3,4-d]pyrimidine (**31** and **32**) as a potential core structure (Table 3). Since a para-nitro or -cyanobenzyl substituent in combination with a *meta*-chlorobenzylamine elicited high antiviral activity, they were retained in our new study. Compared with active inhibitors that contained a 7*H*-pyrrolo[2,3-d]pyrimidine core, new compounds appeared to be generally less toxic and possessed similar anti-ZIKV activity in the reporter assay. Also importantly, they exhibited good to excellent titer-reducing capacity. Among these compounds, **30** had an EC_90_ value of 12.4 µM in the titer reduction assay and a CC_50_ value of 49.3 µM, indicative of its excellent activity and relatively low cytotoxicity. Taken together, these results suggested that 9*H*-purine and 1*H*-pyrazolo[3,4-d]pyrimidine were viable replacements of the 7*H*-pyrrolo[2,3-d]pyrimidine scaffold.

**Figure 2:**
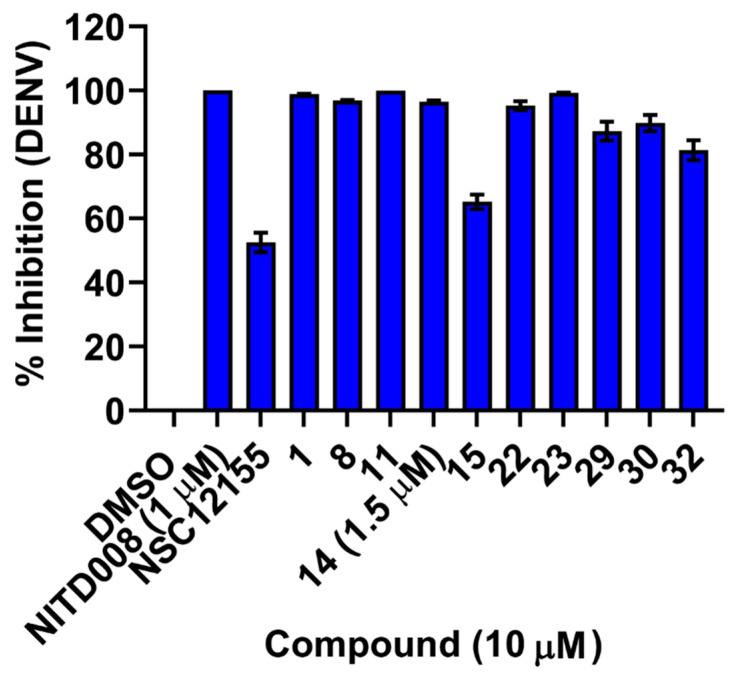
Inhibition of DENV by selected compounds. Huh7 cells were plated in 24 well plate. Next day, cells were inoculated with DENV (MOI = 0.05). After 2 h of infection inoculum was retired and the cells were treated with inhibitors at 8.5 µM (except for compound **14**, 1.5 µM) for 72 h. Next, supernatants were assayed for viral titer by plaque assay and titers were normalized vs DMSO and expressed as % inhibition. The experiment was performed two independent times and each sample was performed in triplicate. The bars depict mean plus standard error of the mean.

Encouraged by the high antiviral activity exhibited by the 4,7-disubstituted 7*H*-pyrrolo[2,3-d]pyrimidines and their analogs, selected compounds were also tested against DENV-2 with NITD008 (1 µM) and NSC12155 (8.5 µM) as reference inhibitors (Figure 2). Compounds were tested at 8.5 µM except for compound **14**, which was used at 1.5 µM. All selected inhibitors showed higher anti-DENV activity than the reference NSC12155 and several compounds offered >90% protection against DENV. Interestingly, among the top inhibitors were compounds **1**, **8**, **11** and **23**, which also exhibited the highest antiviral activity in ZIKV. These preliminary results suggested that the 4,7-disubstituted 7*H*-pyrrolo[2,3-d]pyrimidines and their analogs could represent a new class of antiviral agents against flaviviruses and they might work through a common mechanism of action.

**Scheme 1:**
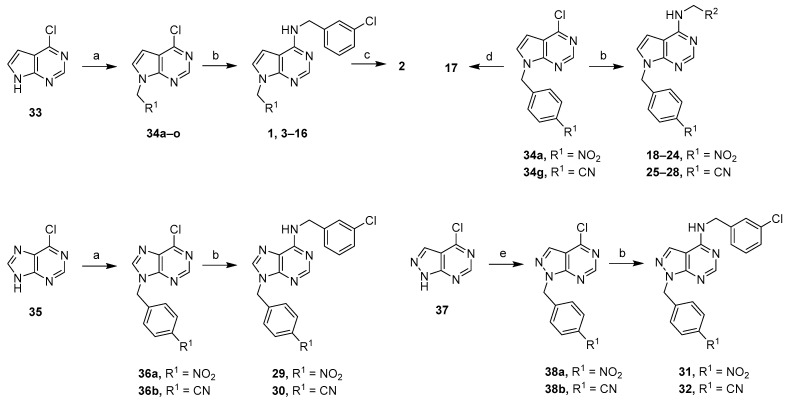
Syntheses of 4,7-disubstituted 7*H*-pyrrolo[2,3-d]pyrimidines and their analogs. *Reagents and conditions*: (a) bromides, K_2_CO_3_, CH_3_CN, rt; (b) amines, DIPEA, 2-methoxyethan-1-ol, 100 °C; (c) SnCl_2_, EtOH, 70 °C; (d) NH_3_, H_2_O, 1,4-dioxane, 120 °C; (e) bromides, Et_3_N, DMF, rt.

The syntheses of 4,7-disubstituted 7*H*-pyrrolo[2,3-d]pyrimidines and their analogs were straightforward (Scheme 1). To study the effect of ring A attached to the 7*H*-pyrrolo[2,3-d]pyrimidine core structure, alkylation (K_2_CO_3_ in CH_3_CN) of 7*H*-pyrrolo[2,3-d]pyrimidine (**33**) was accomplished generally without column purification to give chlorides **34a**–**o**, which were subsequently treated with 3-chlorobenzylamine to afford compounds **1** and **3**–**16** in excellent yields. Reduction of the nitro group in compound **1** with SnCl_2_ gave **2**. To study the effect of ring B, chloride **34a** underwent aminolysis to afford compound **17** while intermediate **34a** and **34g** were treated with various amines to furnish compounds **18**–**24** and **25**–**28**, respectively. To investigate the effect of scaffold replacement, 6-chloropurine (**35**) was alkylated under the same conditions as those that were used for **33**. When **35** was *N*-alkylated with 4-nitrobenzyl bromide, the *N*9 alkylated isomer **36a [14]** was isolated in 61% yield. This regioisomer was confirmed by a combination of ¹H-¹³C heteronuclear multiple quantum correlation (HMQC) and heteronuclear multiple bond correlation spectroscopy (HMBC) (Figure S2). Similarly, chloride **36b [15]** was obtained as an *N*9-alkylated isomer in the presence of K_2_CO_3_. In contrast, different conditions (Et_3_N in DMF) were needed to ensure successful alkylation of 4-chloropyrazolo[3,4-d]pyrimidine (**37**) to give chlorides **38a** and **38b**, whose *N*1 substitution was also established by HMBC experiments. Upon treatment with 3-chlorobenzylamine, the resulting chlorides **36a**–**b** and **38a**–**b**, were converted into purines **29**–**30** and pyrazolo[3,4-d]pyrimidines **31–32**, respectively. The substitution pattern of these regioisomers was further confirmed by a combination of HMQC and HMBC with compound **29** shown as an example in Figure S3.

## 3. Conclusions

Identification of compound **1** as an anti-ZIKV agent has prompted us to investigate its 7*H*-pyrrolo[2,3-d]pyrimidine core structure, leading to several inhibitors including compounds **1**, **8** and **11** that possess promising anti-ZIKV activity, especially in the gold standard titer-reduction assay. Our SAR studies have revealed that rings A and B at positions 7 and 4, respectively, are desired. For ring A, while fused and heterocyclic rings can be tolerated, a phenyl ring decorated with an electron-withdrawing group, preferably nitro and cyano, at the para or meta position gives rise to excellent titer-reducing capability in ZIKV. For ring B, electron-withdrawing group(s) on a phenyl ring are also preferred. However, we have not been able to identify an optimal combination of electron-withdrawing groups. This could be due to the limited scope of electron-withdrawing groups explored in the current study. Furthermore, we have demonstrated that 9*H*-purine or 1*H*-pyrazolo[3,4-d]pyrimidine can serve as an alternative scaffold, generally leading to reduced cytotoxicity. We propose a general pharmacophore model in which the central 7*H*-pyrrolo[2,3-d]pyrimidine ring organizes rings A and B (preferably donned with electro-withdrawing groups) into an orientation that elicits antiviral activity. In addition, 7*H*-pyrrolo[2,3-d]pyrimidine can be replaced by 9*H*-purine or 1*H*-pyrazolo[3,4-d]pyrimidine, which supports a future study on scaffold-hopping. Furthermore, selected compounds also inhibit DENV-2. To our best knowledge, no similar compounds built on a 7*H*-pyrrolo[2,3-d]pyrimidine, 9*H*-purine, or 1*H*-pyrazolo[3,4-d]pyrimidine core structure has been reported as flavivirus inhibitors. Therefore, 4,7-disubstituted 7*H*-pyrrolo[2,3-d]pyrimidines and their analogs hold promise as new chemotypes in the design of antiviral agents against flaviviruses, an important group of human pathogens. Nonetheless, the compounds we have discovered suffer from relatively low therapeutic indices, a critical issue that will be addressed in our future studies.

The molecular target of these antiviral agents is yet to be elucidated. One possible viral target is the ZIKV NS5 MTase, which is required for generating the type I 5′ cap through sequential *N*7 and 2′-*O* methylation of the viral RNA cap using *S*-adenosylmethionine as a methyl donor and releasing *S*-adenosylhomocysteine as a byproduct. Studies have shown that *N*7 methylation is crucial for viral replication [16,17]and 2′-*O* methylation helps viruses evade the host innate immune response.[18] Unfortunately, compound **1** was inactive (IC_50_ > 100 µM) against ZIKV NS5 MTase in our biochemical assay, suggesting that NS5 MTase was unlikely the molecular target of these antiviral compounds. It is also possible that they target a host factor that is essential for virus replication. While identifying the target of a small molecule remains challenging, activity-based protein profiling (ABPP) [19] holds promise because it offers an unbiased approach to identify the molecular target of a wide range of small molecules [20,21,22]. ABPP requires design and synthesis of an ABPP probe, a task that will be facilitated by our experience[23] with this proteomic approach and the SAR information obtained from the current study. In summary, 4,7-disubstituted 7*H*-pyrrolo[2,3-d]pyrimidines and their analogs are promising antiviral agents against flaviviruses ZIKV and DENV. Further exploration of the core structures and appended rings and elucidation of the molecular target are warranted.

## 4. Experimental Section

### 4.1. General Procedures

All commercial reagents were used as provided unless otherwise indicated. An anhydrous solvent dispensing system (J. C. Meyer, Laguna Beach, CA, USA) using two packed columns of neutral alumina was used for drying THF, Et_2_O, and CH_2_Cl_2,_ whereas two packed columns of molecular sieves were used to dry DMF. Solvents were dispensed under argon. Flash chromatography was performed with RediSep R_f_ silica gel columns (Teledyne ISCO, Lincoln, NE, USA) on a CombiFlash^®^ R_f_ system (Teledyne ISCO, Lincoln, NE, USA) using the solvents as indicated. Nuclear magnetic resonance spectra were recorded on a Varian 600 MHz (Palo Alto, CA, USA) or Bruker 400 MHz (Billerica, MA, USA) spectrometer with Me_4_Si or signals from residual solvent as the internal standard for ^1^H or ^13^C. Chemical shifts are reported in ppm, and signals are described as s (singlet), d (doublet), t (triplet), q (quartet), m (multiplet), br s (broad singlet), and dd (double doublet). Values given for coupling constants are first order. High resolution mass spectra were recorded on an TOF II TOF/MS instrument (Agilent, Santa Clara, CA, USA) equipped with either an ESI or APCI interface at the University of Minnesota Center for Drug Design.

### 4.2. Chemistry

*4-Chloro-7-(4-nitrobenzyl)-7H-pyrrolo[2,3-d]pyrimidine* (**34a**). To a suspension of 4-chloro-7*H*-pyrrolo[2,3-d]pyrimidine (**33**, 614 mg, 4.00 mmol) in anhydrous CH_3_CN (40 mL) were added K_2_CO_3_ (1.67 g, 12.1 mmol) and 4-nitrobenzyl bromide (1.04 g, 4.81 mmol). The resulting mixture was allowed to stir at rt for 20 h and concentrated. The residue was suspended in MeOH (20 mL) and poured into stirring water (120 mL). The precipitate was filtered, washed with water and hexanes, dried in vacuo to give compound **34a** as a pale fluffy solid (1.11 g, 96%). ^1^H-NMR (400 MHz, CDCl_3_) δ 8.67 (s, 1H), 8.18 (d, *J* = 8.7 Hz, 2H), 7.35 (d, *J* = 8.7 Hz, 2H), 7.24 (d, *J* = 3.6 Hz, 1H), 6.69 (d, *J* = 3.6 Hz, 1H), 5.57 (s, 2H); ^13^C-NMR (100 MHz, CDCl_3_) δ 152.8, 151.4, 151.3, 147.9, 143.6, 128.9, 128.3, 124.4, 117.7, 100.9, 48.0. HRMS (ESI^+^) *m*/*z* calcd. for C_13_H_10_ClN_4_O_2_ [M + H]^+^ 289.0487, found 289.0487.

*7-Benzyl-4-chloro-7H-pyrrolo[2,3-d]pyrimidine* (**34b**). Compound **34b** was prepared from **33** (154 mg, 1.00 mmol) and benzyl bromide (143 µL, 1.20 mmol) in a fashion similar to the one described for compound **34a** [24]. Brownish syrup, 251 mg, quantitative yield. ^1^H-NMR (400 MHz, CDCl_3_) δ 8.72 (s, 1H), 7.34–7.22 (m, 6H), 6.66 (d, *J* = 3.6 Hz, 1H), 5.48 (s, 2H); ^13^C-NMR (100 MHz, CDCl_3_) δ 151.0, 149.7, 145.1, 135.9, 130.2, 129.1, 128.4, 127.8, 117.5, 100.5, 48.8. HRMS (ESI^+^) *m*/*z* calcd. for C_13_H_11_ClN_3_ [M + H]^+^ 244.0636, found 244.0636.

*4-Chloro-7-(2-nitrobenzyl)-7H-pyrrolo[2,3-d]pyrimidine* (**34c**). Compound **34c** was prepared from **33** (154 mg, 1.00 mmol) and 2-nitrobenzyl bromide (260 mg, 1.20 mmol) in a fashion similar to the one described for compound **34a**. Yellowish solid, 280 mg, yield 97%. ^1^H-NMR (400 MHz, CDCl_3_) δ 8.63 (s, 1H), 8.15 (dd, *J* = 8.0, 1.2 Hz, 1H), 7.53–7.47 (m, 2H), 7.33 (d, *J* = 3.6 Hz, 1H), 6.86 (dd, *J* = 7.8, 1.2 Hz, 1H), 6.71 (d, *J* = 3.6 Hz, 1H), 5.49 (s, 2H); ^13^C-NMR (100 MHz, CDCl_3_) δ 152.7, 151.6, 151.3, 147.7, 134.3, 132.4, 129.7, 129.4, 129.2, 125.6, 117.8, 100.7, 46.0. HRMS (ESI^+^) *m*/*z* calcd. for C_13_H_10_ClN_4_O_2_ [M + H]^+^ 289.0487, found 289.0487.

*4-Chloro-7-(3-nitrobenzyl)-7H-pyrrolo[2,3-d]pyrimidine* (**34d**). Compound **34d** was prepared from **33** (154 mg, 1.00 mmol) and 3-nitrobenzyl bromide (260 mg, 1.20 mmol) in a fashion similar to the one described for compound **34a**. Yellowish solid, 266 mg, yield 92%. ^1^H-NMR (400 MHz, CDCl_3_) δ 8.67 (s, 1H), 8.16 (dd, J = 7.5, 1.8 Hz, 1H), 8.11 (s, 1H), 7.56–7.49 (m, 2H), 7.26 (d, J = 3.6 Hz, 1H), 6.69 (d, J = 3.6 Hz, 1H), 5.57 (s, 2H); ^13^C-NMR (100 MHz, CDCl_3_) δ 152.7, 151.3 (×2), 148.7, 138.6, 133.7, 130.2, 128.8, 123.4, 122.6, 117.7, 100.9, 47.9. HRMS (ESI^+^) *m*/*z* calcd for C_13_H_10_ClN_4_O_2_ [M + H]^+^ 289.0487, found 289.0487.

*4-Chloro-7-(4-(trifluoromethyl)benzyl)-7H-pyrrolo[2,3-d]pyrimidine* (**34e**). Compound **34e** was prepared from **33** (154 mg, 1.00 mmol) and 4-trifluoromethylbenzyl bromide (288 mg, 1.20 mmol) in a fashion similar to the one described for compound **34a** [24]. Pale solid, 307 mg, yield 98%. ^1^H-NMR (400 MHz, CDCl_3_) δ 8.67 (s, 1H), 7.59 (d, *J* = 8.4 Hz, 2H), 7.31 (d, *J* = 8.4 Hz, 2H), 7.22 (d, *J* = 3.6 Hz, 1H), 6.66 (d, *J* = 3.6 Hz, 1H), 5.52 (s, 2H); ^13^C-NMR (100 MHz, CDCl_3_) δ 152.7, 151.4, 151.3, 140.4, 130.7 (q, J_CF_ = 32.3 Hz), 129.0, 127.9, 126.1 (q, J_CF_ = 3.6 Hz), 124.0 (q, J_CF_ = 272.6 Hz), 117.7, 100.6, 48.1. HRMS (ESI^+^) *m*/*z* calcd. for C_14_H_10_ClF_3_N_3_ [M + H]^+^ 312.0510, found 312.0509.

*4-Chloro-7-(4-(methylsulfonyl)benzyl)-7H-pyrrolo[2,3-d]pyrimidine* (**34f**). Compound **34f** was prepared from **33** (154 mg, 1.00 mmol) and 4-methylsulfonylbenzyl bromide (299 mg, 1.20 mmol) in a fashion similar to the one described for compound **34a**. Pale solid, 305 mg, yield 95%. ^1^H-NMR (400 MHz, CDCl_3_) δ 8.67 (s, 1H), 7.89 (d, *J* = 8.4 Hz, 2H), 7.38 (d, *J* = 8.4 Hz, 2H), 7.24 (d, *J* = 3.6 Hz, 1H), 6.69 (d, *J* = 3.6 Hz, 1H), 5.56 (s, 2H), 3.02 (s, 3H); ^13^C-NMR (100 MHz, CDCl_3_) δ 152.7, 151.3 (×2), 142.6, 140.6, 129.0, 128.4, 128.3, 117.7, 100.8, 48.1, 44.6. HRMS (ESI^+^) *m*/*z* calcd. for C_14_H_13_ClN_3_O_2_S [M + H]^+^ 322.0412, found 322.0411.

*4-((4-Chloro-7H-pyrrolo[2,3-d]pyrimidin-7-yl)methyl)benzonitrile* (**34g**). Compound **34g** was prepared from **33** (154 mg, 1.00 mmol) and 4-cyanobenzyl bromide (236 mg, 1.20 mmol) in a fashion similar to the one described for compound **34a** [25]. White solid, 270 mg, quantitative yield. ^1^H-NMR (400 MHz, CDCl_3_) δ 8.66 (s, 1H), 7.62 (d, *J* = 8.4 Hz, 2H), 7.29 (d, *J* = 8.4 Hz, 2H), 7.22 (d, *J* = 3.6 Hz, 1H), 6.68 (d, *J* = 3.6 Hz, 1H), 5.52 (s, 2H); ^13^C-NMR (100 MHz, CDCl_3_) δ 152.8, 151.4 (×2), 141.7, 132.9, 128.9, 128.1, 118.4, 117.7, 112.4, 100.9, 48.2. HRMS (ESI^+^) *m*/*z* calcd. for C_14_H_10_ClN_4_ [M + H]^+^ 269.0589, found 269.0589.

*4-Chloro-7-(3-(trifluoromethyl)benzyl)-7H-pyrrolo[2,3-d]pyrimidine* (**34h**). Compound **34h** was prepared from **33** (154 mg, 1.00 mmol) and 3-trifluoromethylbenzyl bromide (286 mg, 1.20 mmol) in a fashion similar to the one described for compound **34a** [24]. Yellowish solid, 296 mg, yield 95%. ^1^H-NMR (400 MHz, CDCl_3_) δ 8.66 (s, 1H), 7.55 (d, *J* = 8.0 Hz, 1H), 7.51 (s, 1H), 7.44 (t, *J* = 8.0 Hz, 1H), 7.37 (d, *J* = 8.0 Hz, 1H), 7.22 (d, *J* = 3.6 Hz, 1H), 6.64 (d, *J* = 3.6 Hz, 1H), 5.51 (s, 2H); ^13^C-NMR (100 MHz, CDCl_3_) δ 152.6, 151.3, 151.2, 137.5, 131.5 (q, J_CF_ = 33.9 Hz), 131.0, 129.7, 128.9, 125.3 (q, J_CF_ = 3.4 Hz), 124.4 (q, J_CF_ = 3.4 Hz), 123.9 (q, J_CF_ = 273.5 Hz), 117.7, 100.6, 48.1. HRMS (ESI^+^) *m*/*z* calcd. for C_14_H_10_ClF_3_N_3_ [M + H]^+^ 312.0510, found 312.0510.

*4-Chloro-7-(3-(methylsulfonyl)benzyl)-7H-pyrrolo[2,3-d]pyrimidine* (**34i**). Compound **34i** was prepared from **33** (154 mg, 1.00 mmol) and 3-methylsulfonylbenzyl bromide (300 mg, 1.20 mmol) in a fashion similar to the one described for compound **34a**. Pale solid, 288 mg, yield 90%. ^1^H-NMR (400 MHz, CDCl_3_) δ 8.66 (s, 1H), 7.87 (d, *J* = 7.8 Hz, 1H), 7.86 (s, 1H), 7.53 (t, *J* = 7.8 Hz, 1H), 7.46 (d, *J* = 7.8 Hz, 1H), 7.25 (d, *J* = 3.6 Hz, 1H), 6.66 (d, *J* = 3.6 Hz, 1H), 5.54 (s, 2H), 3.02 (s, 3H); ^13^C-NMR (100 MHz, CDCl_3_) δ 152.7, 151.3, 151.2, 141.5, 138.3, 132.9, 130.3, 128.9, 127.3, 126.4, 117.7, 100.8, 48.0, 44.5. HRMS (ESI^+^) *m*/*z* calcd. for C_14_H_13_ClN_3_O_2_S [M + H]^+^ 322.0412, found 322.0408.

*3-((4-Chloro-7H-pyrrolo[2,3-d]pyrimidin-7-yl)methyl)benzonitrile* (**34j**). Compound **34j** was prepared from **33** (154 mg, 1.00 mmol) and 3-cyanobenzyl bromide (235 mg, 1.20 mmol) in a fashion similar to the one described for compound **34a**. Pale solid, 273 mg, quantitative yield. ^1^H-NMR (400 MHz, CDCl_3_) δ 8.66 (s, 1H), 7.59 (td, *J* = 5.0, 1.6 Hz, 1H), 7.49 (s, 1H), 7.46–7.44 (m, 2H), 7.22 (d, *J* = 3.6 Hz, 1H), 6.68 (d, *J* = 3.6 Hz, 1H), 5.49 (s, 2H); ^13^C-NMR (100 MHz, CDCl_3_) δ 152.7, 151.3 (×2), 138.1, 132.0, 131.9, 131.0, 130.0, 128.8, 118.3, 117.7, 113.4, 100.9, 47.9. HRMS (ESI^+^) *m*/*z* calcd. for C_14_H_10_ClN_4_ [M + H]^+^ 269.0589, found 269.0560.

*4-Chloro-7-(naphthalen-1-ylmethyl)-7H-pyrrolo[2,3-d]pyrimidine* (**34k**). Compound **34k** was prepared from **33** (154 mg, 1.00 mmol) and 1-(bromomethyl)naphthalene (265 mg, 1.20 mmol) in a fashion similar to the one described for compound **34a**. Yellowish solid, 298 mg, quantitative yield. ^1^H-NMR (400 MHz, CDCl_3_) δ 8.74 (s, 1H), 7.98 (d, *J* = 8.2 Hz, 1H), 7.89–7.85 (m, 2H), 7.52–7.41 (m, 3H), 7.27 (d, *J* = 8.2 Hz, 1H), 7.09 (d, *J* = 3.6 Hz, 1H), 6.56 (d, *J* = 3.6 Hz, 1H), 5.90 (s, 2H); ^13^C-NMR (100 MHz, CDCl_3_) δ 152.4, 151.2, 151.0, 134.0, 131.5, 131.3, 129.5, 129.1, 129.0, 127.2, 127.0, 126.4, 125.5, 123.0, 117.7, 100.1, 46.7. HRMS (ESI^+^) *m*/*z* calcd. for C_17_H_13_ClN_3_ [M + H]^+^ 294.0793, found 294.0791.

*4-Chloro-7-(naphthalen-2-ylmethyl)-7H-pyrrolo[2,3-d]pyrimidine* (**34l**). Compound **34l** was prepared from **33** (154 mg, 1.00 mmol) and 2-(bromomethyl)naphthalene (265 mg, 1.20 mmol) in a fashion similar to the one described for compound **34a**. Pale solid, 302 mg, quantitative yield. ^1^H-NMR (400 MHz, CDCl_3_) δ 8.70 (s, 1H), 7.80–7.75 (m, 3H), 7.66 (s, 1H), 7.49–7.45 (m, 2H), 7.30 (dd, *J* = 8.2, 1.6 Hz, 1H), 7.24 (d, *J* = 3.6 Hz, 1H), 6.63 (d, *J* = 3.6 Hz, 1H), 5.60 (s, 2H); ^13^C-NMR (100 MHz, CDCl_3_) δ 152.0, 151.3, 150.7, 133.6, 133.3, 133.1, 129.5, 129.1, 127.9, 127.8, 126.8, 126.7, 126.5, 125.4, 117.6, 100.3, 48.8. HRMS (ESI^+^) *m*/*z* calcd. for C_17_H_13_ClN_3_ [M + H]^+^ 294.0793, found 294.0787.

*4-((4-Chloro-7H-pyrrolo[2,3-d]pyrimidin-7-yl)methyl)quinolone* (**34m**). Compound **34m** was prepared from **33** (154 mg, 1.00 mmol) and 4-(bromomethyl)quinoline (266 mg, 1.20 mmol) in a fashion similar to the one described for compound **34a**. Tan solid, 274 mg, yield 93%. ^1^H-NMR (400 MHz, CDCl_3_) δ 8.82 (d, *J* = 4.4 Hz, 1H), 8.70 (s, 1H), 8.17 (d, *J* = 8.2 Hz, 1H), 8.05 (d, *J* = 8.2 Hz, 1H), 7.77 (td, *J* = 8.0, 1.6 Hz, 1H), 7.61 (td, *J* = 8.0, 1.6 Hz, 1H), 7.21 (d, *J* = 3.6 Hz, 1H), 6.85 (d, *J* = 4.4 Hz, 1H), 6.70 (d, *J* = 3.6 Hz, 1H), 5.96 (s, 2H); ^13^C-NMR (100 MHz, CDCl_3_) δ 152.8, 151.4, 151.3, 150.4, 148.5, 141.5, 130.7, 130.0, 129.1, 127.7, 126.0, 122.6, 119.6, 117.7, 100.9, 45.3. HRMS (ESI^+^) *m*/*z* calcd. for C_16_H_12_ClN_4_ [M + H]^+^ 295.0745, found 295.0744.

*4-((4-Chloro-7H-pyrrolo[2,3-d]pyrimidin-7-yl)methyl)-1-naphthonitrile* (**34n**). Compound **34n** was prepared from **33** (154 mg, 1.00 mmol) and 4-(bromomethyl)-1-naphthonitrile (295 mg, 1.20 mmol) in a fashion similar to the one described for compound **34a**. Pale solid, 296 mg, yield 93%. ^1^H-NMR (400 MHz, CDCl_3_) δ 8.71 (s, 1H), 8.31 (d, *J* = 8.4 Hz, 1H), 8.16 (d, *J* = 8.4 Hz, 1H), 7.84 (d, *J* = 7.4 Hz, 1H), 7.74 (td, *J* = 7.4, 1.2 Hz, 1H), 7.67 (td, *J* = 7.4, 1.2 Hz, 1H), 7.17 (d, *J* = 3.6 Hz, 1H), 7.12 (d, *J* = 7.4 Hz, 1H), 6.66 (d, *J* = 3.6 Hz, 1H), 5.98 (s, 2H); ^13^C-NMR (100 MHz, CDCl_3_) δ 152.8, 151.3 (×2), 137.8, 132.7, 132.3, 130.7, 129.0, 128.9, 128.8, 126.4, 124.8, 123.6, 117.7, 117.5, 111.4, 100.9, 46.2. HRMS (ESI^+^) *m*/*z* calcd. for C_18_H_12_ClN_4_ [M + H]^+^ 319.0745, found 319.0731.

*2-Chloro-6-((4-chloro-7H-pyrrolo[2,3-d]pyrimidin-7-yl)methyl)quinolone* (**34o**). Compound **34o** was prepared from **33** (154 mg, 1.00 mmol) and 6-(bromomethyl)-2-chloroquinoline (307 mg, 1.20 mmol) in a fashion similar to the one described for compound **34a**. Pale solid, 329 mg, quantitative yield. ^1^H-NMR (400 MHz, CDCl_3_) δ 8.68 (s, 1H), 8.03 (d, *J* = 8.7 Hz, 1H), 7.98 (d, *J* = 8.7 Hz, 1H), 7.61–7.58 (m, 2H), 7.39 (d, *J* = 8.6 Hz, 1H), 7.27 (d, *J* = 3.6 Hz, 1H), 6.67 (d, *J* = 3.6 Hz, 1H), 5.64 (s, 2H); ^13^C-NMR (100 MHz, CDCl_3_) δ 152.7, 151.4 (×2), 151.3, 147.6, 138.8, 135.4, 130.0, 129.7, 129.1, 126.9, 126.1, 123.2, 117.7, 100.6, 48.4. HRMS (ESI^+^) *m*/*z* calcd. for C_16_H_11_Cl_2_N_4_ [M + H]^+^ 329.0355, found 329.0342.

*N-(3-Chlorobenzyl)-7-(4-nitrobenzyl)-7H-pyrrolo [2,3-d]pyrimidin-4-amine* (**1**). A mixture of **34a** (289 mg, 1.00 mmol), DIPEA (0.35 mL, 2.01 mmol) and 3-chlorobenzylamine (244 µL, 2.00 mmol) in anhydrous 2-methoxyethan-1-ol (10 mL) was heated at 100 °C for 20 h. Additional 3-chlorobenzylamine (244 µL, 2.00 mmol) was added and the resulting mixture was heated at 100 °C for 10 h. After being allowed to cool to rt, the reaction mixture was concentrated and the residue was partitioned between EtOAc (20 mL) and water (40 mL). After separation, the aqueous layer was extracted with EtOAc (20 mL). The combined organic layer was concentrated and the residue was purified by flash column chromatography (30%–90% EtOAc/hexanes) to afford compound **1** as a brownish syrup (370 mg, 94%). ^1^H-NMR (400 MHz, CDCl_3_) δ 8.39 (s, 1H), 8.15 (d, *J* = 8.6 Hz, 2H), 7.38 (s, 1H), 7.30 (d, *J* = 8.6 Hz, 2H), 7.27–7.25 (m, 3H), 6.92 (d, *J* = 3.6 Hz, 1H), 6.40 (d, *J* = 3.6 Hz, 1H), 5.49 (s, 2H), 5.43 (t, *J* = 6.0 Hz, 1H), 4.84 (d, *J* = 6.0 Hz, 2H); ^13^C-NMR (100 MHz, CDCl_3_) δ 156.4, 152.6, 150.4, 147.7, 144.8, 141.1, 134.8, 130.1, 128.1, 127.8 (×2), 125.9, 124.2, 124.1, 103.1, 98.7, 47.5, 44.6. HRMS (ESI^+^) *m*/*z* calcd. for C_20_H_17_ClN_5_O_2_ [M + H]^+^ 394.1065, found 394.1065.

*7-(4-Aminobenzyl)-N-(3-chlorobenzyl)-7H-pyrrolo[2,3-d]pyrimidin-4-amine* (**2**). A mixture of compound **1** (173 mg, 0.439 mmol) and SnCl_2_ (583 mg, 3.07 mmol) in anhydrous EtOH (6 mL) was heated at 70 °C for 21 h. After being allowed to cool to rt, the reaction mixture was diluted with EtOAc (15 mL) and saturated NaHCO_3_ (8 mL) and water (2 mL) were added. The resulting mixture was filtered through a pad of Celite. The organic layer of the filtrate was separated, washed with brine (20 mL) and dried over Na_2_SO_4_. After filtration, the filtrate was concentrated and the residue was purified by flash column chromatography using (0%–10% MeOH/CH_2_Cl_2_) to afford compound **2** as a pale solid (94 mg, 59%). ^1^H-NMR (400 MHz, CDCl_3_) δ 8.42 (s, 1H), 7.37 (s, 1H), 7.26–7.24 (m, 3H), 7.05 (d, *J* = 8.6 Hz, 2H), 6.87 (d, *J* = 3.6 Hz, 1H), 6.61 (d, *J* = 8.6 Hz, 2H), 6.29 (d, *J* = 3.6 Hz, 1H), 5.32 (t, *J* = 6.0 Hz, 1H), 5.25 (s, 2H), 4.83 (d, *J* = 6.0 Hz, 2H), 3.66 (s, 2H); ^13^C-NMR (100 MHz, CDCl_3_) δ 156.3, 152.1, 150.2, 146.2, 141.3, 134.7, 130.1, 129.2, 127.8, 127.7, 127.2, 125.9, 124.3, 115.4, 103.1, 97.6, 47.8, 44.6. HRMS (ESI^+^) *m*/*z* calcd. for C_20_H_19_ClN_5_ [M + H]^+^ 364.1323, found 364.1322.

*7-Benzyl-N-(3-chlorobenzyl)-7H-pyrrolo[2,3-d]pyrimidin-4-amine* (**3**). A mixture of **34b** (74 mg, 0.30 mmol), DIPEA (0.21 mL, 1.2 mmol) and 3-chlorobenzylamine (0.15 mL, 1.2 mmol) in anhydrous 2-methoxyethan-1-ol (3 mL) was heated at 100 °C for 24 h. After being allowed to cool to rt, the reaction mixture was concentrated and the residue was partitioned between EtOAc (10 mL) and water (10 mL). After separation, the organic layer was concentrated and the residue was purified by flash column chromatography (10%–80% EtOAc/hexanes) to afford compound **3** as a yellowish solid (79 mg, 74%). ^1^H-NMR (400 MHz, CDCl_3_) δ 8.42 (s, 1H), 7.38 (s, 1H), 7.33–7.25 (m, 8H), 7.22–7.18 (m, 2H), 6.89 (d, *J* = 3.6 Hz, 1H), 6.33 (d, *J* = 3.6 Hz, 1H), 5.39 (s, 2H), 5.36 (s, 1H), 4.83 (d, *J* = 6.0 Hz, 2H); ^13^C-NMR (100 MHz, CDCl_3_) δ 156.3, 152.2, 150.4, 141.3, 137.4, 134.7, 130.1, 128.9, 127.9, 127.8, 127.7, 127.6, 125.9, 124.5, 103.0, 97.9, 48.1, 44.7. HRMS (ESI^+^) *m*/*z* calcd. for C_20_H_18_ClN_4_ [M + H]^+^ 349.1215, found 349.1214.

*N-(3-Chlorobenzyl)-7-(2-nitrobenzyl)-7H-pyrrolo[2,3-d]pyrimidin-4-amine* (**4**). Compound **4** was prepared from **34c** (80 mg, 0.28 mmol) and 3-chlorobenzylamine (135 µL, 1.10 mmol) in a fashion similar to the one described for compound **3**. Yellow solid, 95 mg, yield 87%. ^1^H-NMR (400 MHz, CDCl_3_) δ 8.37 (s, 1H), 8.13 (dd, *J* = 8.1, 1.6 Hz, 1H), 7.49–7.39 (m, 3H), 7.30–7.26 (m, 3H), 6.99 (d, *J* = 3.6 Hz, 1H), 6.76 (dd, *J* = 8.1, 1.6 Hz, 1H), 6.43 (d, *J* = 3.6 Hz, 1H), 5.82 (s, 2H), 5.41 (t, *J* = 6.0 Hz, 1H), 4.84 (d, *J* = 6.0 Hz, 2H); ^13^C-NMR (100 MHz, CDCl_3_) δ 156.4, 152.6, 150.6, 147.5, 141.2, 134.7, 134.2, 133.7, 130.2, 129.9, 128.6, 127.9, 127.8, 125.9, 125.4, 124.9, 103.2, 98.6, 45.7, 44.7. HRMS (ESI^+^) *m*/*z* calcd. for C_20_H_17_ClN_5_O_2_ [M + H]^+^ 394.1065, found 394.1068.

*N-(3-Chlorobenzyl)-7-(3-nitrobenzyl)-7H-pyrrolo[2,3-d]pyrimidin-4-amine* (**5**). Compound **5** was prepared from **34d** (80 mg, 0.28 mmol) and 3-chlorobenzylamine (135 µL, 1.10 mmol) in a fashion similar to the one described for compound **3**. Yellow solid, 103 mg, yield 94%. ^1^H-NMR (400 MHz, CDCl_3_) δ 8.40 (s, 1H), 8.12 (ddd, *J* = 7.8, 3.8, 1.8 Hz, 1H), 8.05 (s, 1H), 7.53–7.46 (m, 2H), 7.38 (s, 1H), 7.28–7.25 (m, 3H), 6.94 (d, *J* = 3.6 Hz, 1H), 6.41 (d, *J* = 3.6 Hz, 1H), 5.49 (s, 2H), 5.46 (t, *J* = 6.0 Hz, 1H), 4.84 (d, *J* = 6.0 Hz, 2H); ^13^C-NMR (100 MHz, CDCl_3_) δ 156.4, 152.6, 150.3, 148.6, 141.1, 139.7, 134.7, 133.5, 130.1, 129.9, 127.8, 127.7, 125.9, 124.0, 123.0, 122.3, 103.1, 98.7, 47.4, 44.6. HRMS (ESI^+^) *m*/*z* calcd. for C_20_H_17_ClN_5_O_2_ [M + H]^+^ 394.1065, found 394.1065.

*N-(3-Chlorobenzyl)-7-(4-(trifluoromethyl)benzyl)-7H-pyrrolo[2,3-d]pyrimidin-4-amine* (**6**). Compound **6** was prepared from **34e** (62 mg, 0.20 mmol) and 3-chlorobenzylamine (98 µL, 0.80 mmol) in a fashion similar to the one described for compound **3**. Yellow syrup, 74 mg, yield 89%. ^1^H-NMR (400 MHz, CDCl_3_) δ 8.41 (s, 1H), 7.56 (d, *J* = 8.2 Hz, 2H), 7.39 (s, 1H), 7.30–7.27 (m, 5H), 6.91 (d, *J* = 3.6 Hz, 1H), 6.37 (d, *J* = 3.6 Hz, 1H), 5.45 (s, 2H), 5.33 (t, *J* = 6.0 Hz, 1H), 4.85 (d, *J* = 6.0 Hz, 2H); ^13^C-NMR (100 MHz, CDCl_3_) δ 156.4, 152.5, 150.4, 141.5, 141.2, 134.7, 130.2 (q, J_CF_ = 32.5 Hz), 130.1, 127.9, 127.8, 127.7, 125.9 (q, J_CF_ = 3.6 Hz), 125.9, 124.2, 124.1 (q, J_CF_ = 273.8 Hz), 103.1, 98.3, 47.7, 44.7. HRMS (ESI^+^) *m*/*z* calcd. for C_21_H_17_ClF_3_N_5_ [M + H]^+^ 431.1119, found 431.1112.

*N-(3-Chlorobenzyl)-7-(4-(methylsulfonyl)benzyl)-7H-pyrrolo[2,3-d]pyrimidin-4-amine* (**7**). Compound **7** was prepared from **34f** (64 mg, 0.20 mmol) and 3-chlorobenzylamine (98 µL, 0.80 mmol) in a fashion similar to the one described for compound **3**. Pale solid, 82 mg, yield 96%. ^1^H-NMR (400 MHz, CDCl_3_) δ 8.40 (s, 1H), 7.87 (d, *J* = 8.4 Hz, 2H), 7.38 (s, 1H), 7.34 (d, *J* = 8.4 Hz, 2H), 7.29–7.26 (m, 3H), 6.92 (d, *J* = 3.6 Hz, 1H), 6.40 (d, *J* = 3.6 Hz, 1H), 5.49 (s, 2H), 5.39 (t, *J* = 6.0 Hz, 1H), 4.84 (d, *J* = 6.0 Hz, 2H), 3.01 (s, 3H); ^13^C-NMR (100 MHz, CDCl_3_) δ 156.4, 152.6, 150.4, 143.8, 141.2, 140.1, 134.7, 130.1, 128.2, 128.1, 127.8 (×2), 125.9, 124.2, 103.1, 98.6, 47.6, 44.6 (×2). HRMS (ESI^+^) *m*/*z* calcd. for C_21_H_20_ClN_4_O_2_S [M + H]^+^ 427.0990, found 427.0993.

*4-((4-((3-Chlorobenzyl)amino)-7H-pyrrolo[2,3-d]pyrimidin-7-yl)methyl)benzonitrile* (**8**). Compound **8** was prepared from **34g** (54 mg, 0.20 mmol) and 3-chlorobenzylamine (98 µL, 0.80 mmol) in a fashion similar to the one described for compound **3**. Yellow syrup, 70 mg, yield 93%. ^1^H-NMR (400 MHz, CDCl_3_) δ 8.41 (s, 1H), 7.60 (d, *J* = 8.6 Hz, 2H), 7.40 (s, 1H), 7.31–7.27 (m, 5H), 6.92 (d, *J* = 3.6 Hz, 1H), 6.41 (d, *J* = 3.6 Hz, 1H), 5.47 (s, 2H), 5.43 (t, *J* = 6.0 Hz, 1H), 4.86 (d, *J* = 6.0 Hz, 2H); ^13^C-NMR (100 MHz, CDCl_3_) δ 156.4, 152.5, 150.4, 142.9, 141.1, 134.7, 132.7, 130.1, 127.9, 127.8 (×2), 125.9, 124.1, 118.7, 111.8, 103.1, 98.6, 47.7, 44.6. HRMS (ESI^+^) *m*/*z* calcd. for C_21_H_17_ClN_5_ [M + H]^+^ 374.1167, found 374.1174.

*N-(3-Chlorobenzyl)-7-(3-(trifluoromethyl)benzyl)-7H-pyrrolo[2,3-d]pyrimidin-4-amine* (**9**). Compound **9** was prepared from **34h** (79 mg, 0.25 mmol) and 3-chlorobenzylamine (122 µL, 1.00 mmol) in a fashion similar to the one described for compound **3**. Pale solid, 99 mg, yield 94%. ^1^H-NMR (400 MHz, CDCl_3_) δ 8.42 (s, 1H), 7.53 (d, *J* = 8.0 Hz, 2H), 7.48 (s, 1H), 7.44–7.33 (m, 3H), 7.29–7.25 (m, 3H), 6.91 (d, *J* = 3.6 Hz, 1H), 6.37 (d, *J* = 3.6 Hz, 1H), 5.45 (s, 2H), 5.37 (t, *J* = 6.0 Hz, 1H), 4.84 (d, *J* = 6.0 Hz, 2H); ^13^C-NMR (100 MHz, CDCl_3_) δ 156.4, 152.5, 150.4, 141.2, 138.5, 134.7, 131.3 (q, J_CF_ = 32.2 Hz), 130.9, 130.1, 129.5, 127.9, 127.8, 125.9, 124.8 (q, J_CF_ = 3.8 Hz), 124.3 (q, J_CF_ = 3.8 Hz), 124.2 124.0 (q, J_CF_ = 273.1 Hz), 103.1, 98.4, 47.6, 44.7. HRMS (ESI^+^) *m*/*z* calcd. for C_21_H_17_ClF_3_N_5_ [M + H]^+^ 431.1119, found 431.1109.

*N-(3-Chlorobenzyl)-7-(3-(methylsulfonyl)benzyl)-7H-pyrrolo[2,3-d]pyrimidin-4-amine* (**10**). Compound **10** was prepared from **34i** (80 mg, 0.25 mmol) and 3-chlorobenzylamine (122 µL, 1.00 mmol) in a fashion similar to the one described for compound **3**. Pale solid, 105 mg, yield 99%. ^1^H-NMR (400 MHz, CDCl_3_) δ 8.39 (s, 1H), 7.84 (dd, *J* = 8.0, 1.6 Hz, 1H), 7.81 (s, 1H), 7.50 (t, *J* = 8.0 Hz, 1H), 7.43 (dd, *J* = 8.0, 1.6 Hz, 1H), 7.28–7.25 (m, 3H), 6.92 (d, *J* = 3.6 Hz, 1H), 6.39 (d, *J* = 3.6 Hz, 1H), 5.47 (s, 2H), 5.41 (t, *J* = 6.0 Hz, 1H), 4.84 (d, *J* = 6.0 Hz, 2H), 3.01 (s, 3H); ^13^C-NMR (100 MHz, CDCl_3_) δ 156.4, 152.5, 150.3, 141.2, 141.1, 139.5, 134.7, 132.7, 130.2, 130.1, 127.9, 127.8, 126.9, 126.1, 125.9, 124.1, 103.1, 98.6, 47.5, 44.6 (×2). HRMS (ESI_+_) *m*/*z* calcd. for C_21_H_20_ClN_4_O_2_S [M + H]^+^ 427.0990, found 427.0980.

*3-((4-((3-Chlorobenzyl)amino)-7H-pyrrolo[2,3-d]pyrimidin-7-yl)methyl)benzonitrile* (**11**). Compound **11** was prepared from **34j** (67 mg, 0.25 mmol) and 3-chlorobenzylamine (122 µL, 1.00 mmol) in a fashion similar to the one described for compound **3**. Pale solid, 93 mg, quantitative yield. ^1^H-NMR (400 MHz, CDCl_3_) δ 8.40 (s, 1H), 7.55 (dd, *J* = 8.2, 1.8 Hz, 1H), 7.44–7.38 (m, 4H), 7.29–7.25 (m, 3H), 6.90 (d, *J* = 3.6 Hz, 1H), 6.39 (d, *J* = 3.6 Hz, 1H), 5.42 (t, *J* = 6.0 Hz, 1H), 5.41 (s, 2H), 4.84 (d, *J* = 6.0 Hz, 2H); ^13^C-NMR (100 MHz, CDCl_3_) δ 156.4, 152.6, 150.3, 141.1, 139.2, 134.7, 131.8, 131.6, 130.8, 130.1, 129.8, 127.9, 127.8, 125.9, 124.0, 118.6, 113.1, 103.1, 98.6, 47.3, 44.6. HRMS (ESI^+^) *m*/*z* calcd. for C_21_H_17_ClN_5_ [M + H]^+^ 374.1167, found 374.1166.

*N-(3-Chlorobenzyl)-7-(naphthalen-1-ylmethyl)-7H-pyrrolo[2,3-d]pyrimidin-4-amine* (**12**). Compound **12** was prepared from **34k** (89 mg, 0.30 mmol) and 3-chlorobenzylamine (0.15 mL, 1.2 mmol) in a fashion similar to the one described for compound **3**. Yellowish solid, 91 mg, yield 75%. ^1^H-NMR (400 MHz, CDCl_3_) δ 8.45 (s, 1H), 7.81–7.75 (m, 3H), 7.65 (s, 1H), 7.48–7.44 (m, 2H), 7.39 (s, 1H), 7.33 (dd, *J* = 8.6, 1.8 Hz, 1H), 7.28–7.24 (m, 3H), 6.93 (d, *J* = 3.6 Hz, 1H), 6.34 (d, *J* = 3.6 Hz, 1H), 5.55 (s, 2H), 5.39 (t, *J* = 6.0 Hz, 1H), 4.84 (d, *J* = 6.0 Hz, 2H); ^13^C-NMR (100 MHz, CDCl_3_) δ 156.3, 152.3, 150.4, 141.3, 134.9, 134.7, 133.4, 133.0, 130.1, 128.3, 128.0, 127.8 (×2), 127.7, 126.5 (×2), 126.2, 125.9, 125.6, 124.5, 103.1, 98.0, 48.3, 44.7. HRMS (ESI^+^) *m*/*z* calcd. for C_24_H_20_ClN_4_ [M + H]^+^ 399.1371, found 399.1367.

*N-(3-Chlorobenzyl)-7-(naphthalen-2-ylmethyl)-7H-pyrrolo[2,3-d]pyrimidin-4-amine* (**13**). Compound **13** was prepared from **34l** (90 mg, 0.31 mmol) and 3-chlorobenzylamine (0.15 mL, 1.2 mmol) in a fashion similar to the one described for compound **3**. Pale solid, 111 mg, yield 91%. ^1^H-NMR (400 MHz, CDCl_3_) δ 8.49 (s, 1H), 8.04 (dd, *J* = 7.4, 1.8 Hz, 1H), 7.87 (dd, *J* = 7.4, 1.8 Hz, 1H), 7.83 (d, *J* = 8.2 Hz, 1H), 7.51–7.46 (m, 2H), 7.43 (d, *J* = 8.2 Hz, 1H), 7.39 (s, 1H), 7.29–7.25 (m, 3H), 7.22 (d, *J* = 7.4 Hz, 1H), 6.80 (d, *J* = 3.6 Hz, 1H), 6.29 (d, *J* = 3.6 Hz, 1H), 5.84 (s, 2H), 5.32 (t, *J* = 6.0 Hz, 1H), 4.84 (d, *J* = 6.0 Hz, 2H); ^13^C-NMR (100 MHz, CDCl_3_) δ 156.4, 152.3, 150.2, 141.3, 134.7, 134.0, 132.6, 131.4, 130.1, 129.1, 128.9, 127.9, 127.8, 126.9, 126.7, 126.2, 125.9, 125.5, 124.3, 123.3, 103.1, 97.8, 46.0, 44.7. HRMS (ESI^+^) *m*/*z* calcd. for C_24_H_20_ClN_4_ [M + H]^+^ 399.1371, found 399.1365.

*N-(3-Chlorobenzyl)-7-(quinolin-4-ylmethyl)-7H-pyrrolo[2,3-d]pyrimidin-4-amine* (**14**). Compound **14** was prepared from **34m** (74 mg, 0.25 mmol) and 3-chlorobenzylamine (122 µL, 1.00 mmol) in a fashion similar to the one described for compound **3**. Pale solid, 84 mg, yield 84%. ^1^H-NMR (400 MHz, CDCl_3_) δ 8.80 (d, *J* = 4.4 Hz, 1H), 8.44 (s, 1H), 8.15 (dd, *J* = 8.4, 1.2 Hz, 1H), 8.08 (dd, *J* = 8.4, 1.2 Hz, 1H), 7.75 (td, *J* = 7.8, 1.3 Hz, 1H), 7.60 (td, *J* = 7.8, 1.3 Hz, 1H), 7.40 (s, 1H), 7.31–7.27 (m, 3H), 6.90 (d, *J* = 3.6 Hz, 1H), 6.81 (d, *J* = 4.4 Hz, 1H), 6.41 (d, *J* = 3.6 Hz, 1H), 5.89 (s, 2H), 5.37 (t, *J* = 6.0 Hz, 1H), 4.86 (d, *J* = 6.0 Hz, 2H); ^13^C-NMR (100 MHz, CDCl_3_) δ 156.5, 152.6, 150.5 (×2), 148.4, 142.7, 141.2, 134.8, 130.5, 130.2, 129.8, 127.9, 127.8, 127.4, 126.2, 126.0, 124.4, 122.8, 119.4, 103.1, 98.6, 44.9, 44.7. HRMS (ESI^+^) *m*/*z* calcd. for C_23_H_19_ClN_4_ [M + H]^+^ 400.1323, found 400.1311.

*4-((4-((3-Chlorobenzyl)amino)-7H-pyrrolo[2,3-d]pyrimidin-7-yl)methyl)-1-naphthonitrile* (**15**). Compound **15** was prepared from **34n** (80 mg, 0.25 mmol) and 3-chlorobenzylamine (122 µL, 1.00 mmol) in a fashion similar to the one described for compound **3**. Pale solid, 104 mg, yield 98%. ^1^H-NMR (400 MHz, CDCl_3_) δ 8.43 (s, 1H), 8.28 (dd, *J* = 8.4, 1.2 Hz, 1H), 8.18 (dd, *J* = 8.4, 1.2 Hz, 1H), 7.81 (d, *J* = 7.8 Hz, 1H), 7.72 (td, *J* = 7.8, 1.3 Hz, 1H), 7.66 (td, *J* = 7.8, 1.3 Hz, 1H), 7.39 (s, 1H), 7.29–7.26 (m, 3H), 7.04 (d, *J* = 7.8 Hz, 1H), 6.85 (d, *J* = 3.6 Hz, 1H), 6.38 (d, *J* = 3.6 Hz, 1H), 5.90 (s, 2H), 5.42 (t, *J* = 6.0 Hz, 1H), 4.85 (d, *J* = 6.0 Hz, 2H); ^13^C-NMR (100 MHz, CDCl_3_) δ 156.5, 152.6, 150.3, 141.1, 139.0, 134.7, 132.6, 132.4, 130.7, 130.1, 128.8, 128.5, 127.9, 127.8, 126.2, 125.9, 124.4, 124.2, 123.8, 117.8, 110.8, 103.1, 98.6, 45.8, 44.7. HRMS (ESI^+^) *m*/*z* calcd. for C_25_H_19_ClN_5_ [M + H]^+^ 424.1323, found 424.1294.

*N-(3-Chlorobenzyl)-7-((2-chloroquinolin-6-yl)methyl)-7H-pyrrolo[2,3-d]pyrimidin-4-amine* (**16**). Compound **16** was prepared from **34o** (310 mg, 0.942 mmol) and 3-chlorobenzylamine (0.46 mL, 3.78 mmol) in a fashion similar to the one described for compound **3**. Pale solid, 381 mg, yield 93%. ^1^H-NMR (400 MHz, CDCl_3_) δ 8.43 (s, 1H), 8.01 (d, *J* = 8.6 Hz, 1H), 7.96 (d, *J* = 8.6 Hz, 1H), 7.60–7.56 (m, 2H), 7.39 (s, 1H), 7.36 (d, *J* = 8.6 Hz, 1H), 7.29–7.25 (m, 3H), 6.95 (d, *J* = 3.6 Hz, 1H), 6.39 (d, *J* = 3.6 Hz, 1H), 5.57 (s, 2H), 5.42 (t, *J* = 6.0 Hz, 1H), 4.85 (d, *J* = 6.0 Hz, 2H); ^13^C-NMR (100 MHz, CDCl_3_) δ 156.4, 152.5, 151.0, 150.4, 147.5, 141.2, 138.9, 136.5, 134.7, 130.1 (×2), 129.4, 127.8 (×2), 126.9, 125.9, 125.8, 124.3, 122.9, 103.1, 98.4, 47.8, 44.6. HRMS (ESI^+^) *m*/*z* calcd. for C_23_H_18_Cl_2_N_5_ [M + H]^+^ 434.0934, found 434.0910.

*7-(4-Nitrobenzyl)-7H-pyrrolo[2,3-d]pyrimidin-4-amine* (**17**). A mixture of compound **34a** (201 mg, 0.696 mmol) in 1,4-dioxane (5.0 mL) and strong ammonia (5.0 mL) in a seal tube was heated at 120 °C for 4 h. After being allowed to cool to rt, the reaction mixture was concentrated and the residue was purified by flash column chromatography (0%–10% MeOH/CH_2_Cl_2_) to afford compound **17** as a yellow solid (109 mg, 58%). ^1^H-NMR (400 MHz, DMSO-*d*_6_) δ 8.16 (d, *J* = 8.7 Hz, 2H), 8.04 (s, 1H), 7.38 (d, *J* = 8.7 Hz, 2H), 7.24 (d, *J* = 3.6 Hz, 1H), 7.02 (s, 2H), 6.61 (d, *J* = 3.6 Hz, 1H), 5.47 (s, 2H); ^13^C-NMR (100 MHz, DMSO-*d*_6_) δ 157.7, 152.1, 149.8, 147.0, 146.2, 128.4, 124.5, 123.9, 102.6, 99.6, 46.8. HRMS (ESI^+^) *m*/*z* calcd. for C_13_H_12_N_5_O_2_ [M + H]^+^ 270.0986, found 270.0985.

*N-Benzyl-7-(4-nitrobenzyl)-7H-pyrrolo[2,3-d]pyrimidin-4-amine* (**18**). Compound **18** was prepared from **34a** (58 mg, 0.20 mmol) and benzylamine (87 µL, 0.80 mmol) in a fashion similar to the one described for compound **3**. Yellow solid, 68 mg, yield 95%. ^1^H-NMR (400 MHz, CDCl_3_) δ 8.40 (s, 1H), 8.15 (d, *J* = 8.7 Hz, 2H), 7.42–7.35 (m, 4H), 7.33–7.28 (m, 3H), 6.90 (d, *J* = 3.6 Hz, 1H), 6.39 (d, *J* = 3.6 Hz, 1H), 5.49 (s, 2H), 5.35 (t, *J* = 6.0 Hz, 1H), 4.86 (d, *J* = 6.0 Hz, 2H); ^13^C-NMR (100 MHz, CDCl_3_) δ 156.6, 152.7, 150.4, 147.7, 144.9, 138.8, 129.0, 128.1, 127.9, 127.8, 124.2, 123.9, 103.1, 98.8, 47.5, 45.4. HRMS (ESI^+^) *m*/*z* calcd. for C_20_H_18_N_5_O_2_ [M + H]^+^ 360.1455, found 360.1450.

*N-(2-Chlorobenzyl)-7-(4-nitrobenzyl)-7H-pyrrolo[2,3-d]pyrimidin-4-amine* (**19**). Compound **19** was prepared from **34a** (80 mg, 0.28 mmol) and 2-chlorobenzylamine (134 µL, 1.11 mmol) in a fashion similar to the one described for compound **3**. Yellow semi-solid, 108 mg, yield 99%. ^1^H-NMR (400 MHz, CDCl_3_) δ 8.39 (s, 1H), 8.14 (d, *J* = 8.6 Hz, 2H), 7.51–7.48 (m, 1H), 7.40–7.37 (m, 1H), 7.29 (d, *J* = 8.6 Hz, 2H), 7.25–7.21 (m, 2H), 6.90 (d, *J* = 3.6 Hz, 1H), 6.39 (d, *J* = 3.6 Hz, 1H), 5.52 (t, *J* = 6.0 Hz, 1H), 5.48 (s, 2H), 4.95 (d, *J* = 6.0 Hz, 2H); ^13^C-NMR (100 MHz, CDCl_3_) δ 156.5, 152.6, 150.4, 147.6, 144.8, 136.3, 133.7, 130.0, 129.7, 129.0, 128.1, 127.2, 124.2, 124.0, 103.2, 98.8, 47.8, 43.1. HRMS (ESI^+^) *m*/*z* calcd. for C_20_H_17_ClN_5_O_2_ [M + H]^+^ 394.1065, found 394.1064.

*N-(4-Chlorobenzyl)-7-(4-nitrobenzyl)-7H-pyrrolo[2,3-d]pyrimidin-4-amine* (**20**). Compound **20** was prepared from **34a** (80 mg, 0.28 mmol) and 4-chlorobenzylamine (135 µL, 1.11 mmol) in a fashion similar to the one described for compound **3**. Yellow solid, 109 mg, quantitative yield. ^1^H-NMR (400 MHz, CDCl_3_) δ 8.39 (s, 1H), 8.15 (d, *J* = 8.6 Hz, 2H), 7.33–7.29 (m, 6H), 6.91 (d, *J* = 3.6 Hz, 1H), 6.39 (d, *J* = 3.6 Hz, 1H), 5.49 (s, 2H), 5.34 (t, *J* = 6.0 Hz, 1H), 4.83 (d, *J* = 6.0 Hz, 2H); ^13^C-NMR (100 MHz, CDCl_3_) δ 156.4, 152.6, 150.4, 147.7, 144.8, 137.5, 133.5, 129.2, 129.0, 128.1, 124.2, 124.1, 103.1, 98.7, 47.5, 44.6. HRMS (ESI^+^) *m*/*z* calcd. for C_20_H_17_ClN_5_O_2_ [M + H]^+^ 394.1065, found 394.1062.

*N-(2,3-Dichlorobenzyl)-7-(4-nitrobenzyl)-7H-pyrrolo[2,3-d]pyrimidin-4-amine* (**21**). Compound **21** was prepared from **34a** (58 mg, 0.20 mmol) and 2,3-dichlorobenzylamine (0.11 mL, 0.82 mmol) in a fashion similar to the one described for compound **3**. Yellowish solid, 86 mg, quantitative yield. ^1^H-NMR (400 MHz, CDCl_3_) δ 8.38 (s, 1H), 8.15 (d, *J* = 8.6 Hz, 2H), 7.43–7.38 (m, 2H), 7.31 (d, *J* = 8.6 Hz, 2H), 7.17 (t, *J* = 7.7 Hz, 1H), 6.92 (d, *J* = 3.6 Hz, 1H), 6.39 (d, *J* = 3.6 Hz, 1H), 5.54 (t, *J* = 6.0 Hz, 1H), 5.48 (s, 2H), 4.96 (d, *J* = 6.0 Hz, 2H); ^13^C-NMR (100 MHz, CDCl_3_) δ 156.4, 152.5, 150.4, 147.7, 144.8, 138.8, 133.4, 131.8, 129.7, 128.1, 127.9, 127.5, 124.2 (×2), 103.2, 98.6, 47.5, 43.7. HRMS (ESI^+^) *m*/*z* calcd. for C_20_H_16_Cl_2_N_5_O_2_ [M + H]^+^ 428.0676, found 428.0660.

*N-(2,5-Dichlorobenzyl)-7-(4-nitrobenzyl)-7H-pyrrolo[2,3-d]pyrimidin-4-amine* (**22**). Compound **22** was prepared from **34a** (58 mg, 0.20 mmol) and 2,5-dichlorobenzylamine (0.11 mL, 0.82 mmol) in a fashion similar to the one described for compound **3**. Yellowish solid, 83 mg, yield 96%. ^1^H-NMR (400 MHz, CDCl_3_) δ 8.41 (s, 1H), 8.16 (d, *J* = 8.6 Hz, 2H), 7.48 (d, *J* = 2.4 Hz, 1H), 7.34–7.30 (m, 3H), 7.20 (dd, *J* = 8.4, 2.4 Hz, 1H), 6.93 (d, *J* = 3.6 Hz, 1H), 6.40 (d, *J* = 3.6 Hz, 1H), 5.51 (t, *J* = 6.0 Hz, 1H), 5.49 (s, 2H), 4.92 (d, *J* = 6.0 Hz, 2H); ^13^C-NMR (100 MHz, CDCl_3_) δ 156.3, 152.5, 150.4, 147.7, 144.7, 138.2, 133.1, 131.7, 130.8, 129.6, 128.9, 128.1, 124.2 (×2), 103.2, 98.6, 47.5, 42.7. HRMS (ESI^+^) *m*/*z* calcd. for C_20_H_16_Cl_2_N_5_O_2_ [M + H]^+^ 428.0676, found 428.0670.

*N-(3,4-Dichlorobenzyl)-7-(4-nitrobenzyl)-7H-pyrrolo[2,3-d]pyrimidin-4-amine* (**23**). Compound **23** was prepared from **34a** (46 mg, 0.16 mmol) and 3,4-dichlorobenzylamine (115 mg, 0.653 mmol) in a fashion similar to the one described for compound **3**. Yellowish solid, 65 mg, yield 95%. ^1^H-NMR (400 MHz, CDCl_3_) δ 8.39 (s, 1H), 8.16 (d, *J* = 8.6 Hz, 2H), 7.49 (d, *J* = 2.3 Hz, 1H), 7.41 (d, *J* = 8.2 Hz, 1H), 7.32 (d, *J* = 8.6 Hz, 2H), 7.14 (dd, *J* = 8.4, 2.3 Hz, 1H), 6.94 (d, *J* = 3.6 Hz, 1H), 6.40 (d, *J* = 3.6 Hz, 1H), 5.50 (s, 2H), 5.40 (t, *J* = 6.0 Hz, 1H), 4.83 (d, *J* = 6.0 Hz, 2H); ^13^C-NMR (100 MHz, CDCl_3_) δ 156.3, 152.5, 150.4, 147.7, 144.7, 138.1, 133.0, 132.0, 130.8, 129.6, 128.1, 127.1, 124.3, 124.2, 103.1, 98.6, 47.5, 44.1. HRMS (ESI^+^) *m*/*z* calcd. for C_20_H_16_Cl_2_N_5_O_2_ [M + H]^+^ 428.0676, found 428.0665.

*N-(Naphthalen-1-ylmethyl)-7-(4-nitrobenzyl)-7H-pyrrolo[2,3-d]pyrimidin-4-amine* (**24**). Compound **24** was prepared from **34a** (58 mg, 0.20 mmol) and naphthalen-1-ylmethanamine (0.12 mL, 0.82 mmol) in a fashion similar to the one described for compound **3**. Yellowish solid, 63 mg, yield 76%. ^1^H-NMR (400 MHz, CDCl_3_) δ 8.48 (s, 1H), 8.15 (d, *J* = 8.8 Hz, 2H), 8.13–8.11 (m, 1H), 7.92–7.89 (m, 1H), 7.85 (d, *J* = 8.2 Hz, 1H), 7.58–7.51 (m, 3H), 7.46 (dd, *J* = 8.2, 6.8 Hz, 1H), 7.31 (d, *J* = 8.8 Hz, 2H), 6.89 (d, *J* = 3.6 Hz, 1H), 6.32 (d, *J* = 3.6 Hz, 1H), 5.49 (s, 2H), 5.30–5.28 (m, 3H); ^13^C-NMR (100 MHz, CDCl_3_) δ 156.4, 152.7, 150.3, 147.7, 144.9, 134.1, 133.9, 131.7, 129.0, 128.8, 128.1, 126.8 (×2), 126.2, 125.6, 124.2, 123.9, 123.7, 103.1, 98.8, 47.5, 43.7. HRMS (ESI^+^) *m*/*z* calcd. for C_24_H_20_N_5_O_2_ [M + H]^+^ 410.1612, found 410.1611.

*4-((4-((3-Methylbenzyl)amino)-7H-pyrrolo[2,3-d]pyrimidin-7-yl)methyl)benzonitrile* (**25**). Compound **25** was prepared from **34g** (45 mg, 0.17 mmol) and 3-methylbenzylamine (85 µL, 0.68 mmol) in a fashion similar to the one described for compound **3**. Yellowish semi-solid, 56 mg, yield 95%. ^1^H-NMR (400 MHz, CDCl_3_) δ 8.40 (s, 1H), 7.58 (d, *J* = 8.4 Hz, 2H), 7.27–7.18 (m, 5H), 7.12 (d, *J* = 7.4 Hz, 1H), 6.88 (d, *J* = 3.6 Hz, 1H), 6.38 (d, *J* = 3.6 Hz, 1H), 5.44 (s, 2H), 5.32 (t, *J* = 6.0 Hz, 1H), 4.81 (d, *J* = 6.0 Hz, 2H), 2.35 (s, 3H); ^13^C-NMR (100 MHz, CDCl_3_) δ 156.5, 152.6, 150.3, 142.9, 138.7 (×2), 132.7, 128.8, 128.7, 128.5, 127.9, 125.0, 123.9, 118.7, 111.8, 103.0, 98.8, 47.7, 45.5, 21.5. HRMS (ESI^+^) *m*/*z* calcd. for C_22_H_20_N_5_ [M + H]^+^ 354.1713, found 354.1712.

*4-((4-((2-Chlorobenzyl)amino)-7H-pyrrolo[2,3-d]pyrimidin-7-yl)methyl)benzonitrile* (**26**). Compound **26** was prepared from **34g** (45 mg, 0.17 mmol) and 2-chlorobenzylamine (81 µL, 0.67 mmol) in a fashion similar to the one described for compound **3**. Pale solid, 55 mg, yield 88%. ^1^H-NMR (400 MHz, CDCl_3_) δ 8.39 (s, 1H), 7.58 (d, *J* = 8.6 Hz, 2H), 7.49 (dd, *J* = 7.4, 3.0 Hz, 1H), 7.39 (dd, *J* = 7.4, 3.0 Hz, 1H), 7.26–7.22 (m, 4H), 6.86 (d, *J* = 3.6 Hz, 1H), 6.38 (d, *J* = 3.6 Hz, 1H), 5.47 (t, *J* = 6.0 Hz, 1H), 5.44 (s, 2H), 4.95 (d, *J* = 6.0 Hz, 2H); ^13^C-NMR (100 MHz, CDCl_3_) δ 156.5, 152.6, 150.4, 142.9, 136.3, 133.7, 132.7, 130.0, 129.8, 129.0, 127.9, 127.2, 124.1, 118.7, 111.8, 103.1, 98.6, 47.7, 43.1. HRMS (ESI^+^) *m*/*z* calcd. for C_21_H_17_ClN_5_ [M + H]^+^ 374.1167, found 374.1167.

*4-((4-((2,3-Dichlorobenzyl)amino)-7H-pyrrolo[2,3-d]pyrimidin-7-yl)methyl)benzonitrile* (**27**). Compound **27** was prepared from **34g** (45 mg, 0.17 mmol) and 2,3-dichlorobenzylamine (118 mg, 0.67 mmol) in a fashion similar to the one described for compound **3**. Pale solid, 58 mg, yield 85%. ^1^H-NMR (400 MHz, CDCl_3_) δ 8.37 (s, 1H), 7.58 (d, *J* = 8.4 Hz, 2H), 7.40 (dd, *J* = 7.8, 1.7 Hz, 2H), 7.24 (d, *J* = 8.4 Hz, 2H), 7.16 (t, *J* = 7.8 Hz, 1H), 6.90 (d, *J* = 3.6 Hz, 1H), 6.38 (d, *J* = 3.6 Hz, 1H), 5.54 (t, *J* = 6.0 Hz, 1H), 5.44 (s, 2H), 4.96 (d, *J* = 6.0 Hz, 2H); ^13^C-NMR (100 MHz, CDCl_3_) δ 156.4, 152.5, 150.4, 142.8, 138.8, 133.4, 132.7, 131.8, 129.6, 128.0, 127.9, 127.6, 124.2, 118.7, 111.9, 103.2, 98.5, 47.7, 43.6. HRMS (ESI^+^) *m*/*z* calcd. for C_21_H_16_Cl_2_N_5_ [M + H]^+^ 408.0777, found 408.0777.

*4-((4-((2,5-Dichlorobenzyl)amino)-7H-pyrrolo[2,3-d]pyrimidin-7-yl)methyl)benzonitrile* (**28**). Compound **28** was prepared from **34g** (45 mg, 0.17 mmol) and 2,5-dichlorobenzylamine (90 µL, 0.67 mmol) in a fashion similar to the one described for compound **3**. Pale solid, 54 mg, yield 79%. ^1^H-NMR (400 MHz, CDCl_3_) δ 8.39 (s, 1H), 7.59 (d, *J* = 8.4 Hz, 2H), 7.48 (d, *J* = 2.5 Hz, 1H), 7.32 (d, *J* = 8.6 Hz, 1H), 7.26 (d, *J* = 8.4 Hz, 2H), 7.20 (dd, *J* = 8.6, 2.5 Hz, 1H), 6.91 (d, *J* = 3.6 Hz, 1H), 6.39 (d, *J* = 3.6 Hz, 1H), 5.48 (t, *J* = 6.0 Hz, 1H), 5.44 (s, 2H), 4.92 (d, *J* = 6.0 Hz, 2H); ^13^C-NMR (100 MHz, CDCl_3_) δ 156.3, 152.5, 150.4, 142.8, 138.3, 133.1, 132.8, 131.7, 130.8, 129.6, 128.9, 128.0, 124.3, 118.7, 111.9, 103.2, 98.5, 47.7, 42.7. HRMS (ESI^+^) *m*/*z* calcd. for C_21_H_16_Cl_2_N_5_ [M + H]^+^ 408.0777, found 408.0777.

*6-Chloro-9-(4-nitrobenzyl)-9H-purine* (**36a**). To a suspension of 6-chloropurine (**35**, 309 mg, 2.00 mmol) in anhydrous CH_3_CN (20 mL) were added K_2_CO_3_ (830 mg, 6.00 mmol) and 4-nitrobenzyl bromide (516 mg, 2.39 mmol) [14,15]. The resulting mixture was allowed to stir at rt for 22 h and concentrated. The residue was suspended in MeOH (10 mL) and poured into stirring water (60 mL). The precipitate was filtered, washed with water and dried. The residue was purified by flash column chromatography (10%–100% EtOAc/hexanes) to afford compound **36a** as a white solid (354 mg, 61%). ^1^H-NMR (400 MHz, CDCl_3_) δ 8.78 (s, 1H), 8.23 (d, *J* = 8.8 Hz, 2H), 8.17 (s, 1H), 7.48 (d, *J* = 8.8 Hz, 2H), 5.58 (s, 2H); ^13^C-NMR (100 MHz, CDCl_3_) δ 152.6, 151.9, 151.8, 148.3, 144.7, 141.6, 131.7, 128.7, 124.6, 47.2. HRMS (ESI^+^) *m*/*z* calcd. for C_12_H_9_ClN_5_O_2_ [M + H]^+^ 290.0439, found 290.0437.

*4-((6-Chloro-9H-purin-9-yl)methyl)benzonitrile* (**36b**). Compound **36b** was prepared from 6-chloropurine (**35**, 155 mg, 1.00 mmol) and 4-cyanobenzyl bromide (235 mg, 1.20 mmol) in a fashion similar to the one described for compound **36a [15]**. White solid, 171 mg, yield 63%. ^1^H-NMR (400 MHz, CDCl_3_) δ 8.76 (s, 1H), 8.15 (s, 1H), 7.65 (d, *J* = 8.6 Hz, 2H), 7.40 (d, *J* = 8.8 Hz, 2H), 5.52 (s, 2H); ^13^C-NMR (100 MHz, CDCl_3_) δ 152.5, 151.9, 151.7, 144.8, 139.8, 133.1, 131.7, 128.5, 118.1, 113.1, 47.4. HRMS (ESI^+^) *m*/*z* calcd. for C_13_H_9_ClN_5_ [M + H]^+^ 270.0541, found 270.0543.

*N-(3-Chlorobenzyl)-9-(4-nitrobenzyl)-9H-purin-6-amine* (**29**). Compound **29** was prepared from **36a** (72 mg, 0.25 mmol) and 3-chlorobenzylamine (122 µL, 1.00 mmol) in a fashion similar to the one described for compound **3**. Pale solid, 93 mg, yield 95%. ^1^H-NMR (400 MHz, CDCl_3_) δ 8.42 (s, 1H), 8.20 (d, *J* = 8.6 Hz, 2H), 7.71 (s, 1H), 7.42 (d, *J* = 8.6 Hz, 2H), 7.37 (s, 1H), 7.25–7.23 (m, 3H), 6.40 (t, *J* = 6.0 Hz, 1H), 5.47 (s, 2H), 4.88 (s, 2H); ^13^C-NMR (100 MHz, CDCl_3_) δ 154.9, 153.8, 149.7, 148.0, 142.9, 140.8, 139.6, 134.7, 130.1, 128.5, 127.8 (×2), 125.9, 125.4, 119.8, 46.5, 44.1. HRMS (ESI^+^) *m*/*z* calcd. for C_19_H_16_ClN_6_O_2_ [M + H]^+^ 395.1018, found 395.1002.

*4-((6-((3-Chlorobenzyl)amino)-9H-purin-9-yl)methyl)benzonitrile* (**30**). Compound **30** was prepared from **36b** (49 mg, 0.18 mmol) and 3-chlorobenzylamine (88 µL, 0.72 mmol) in a fashion similar to the one described for compound **3**. Pale solid, 51 mg, yield 75%. ^1^H-NMR (400 MHz, CDCl_3_) δ 8.42 (s, 1H), 7.70 (s, 1H), 7.64 (d, *J* = 8.6 Hz, 2H), 7.37–7.35 (m, 3H), 7.25–7.23 (m, 3H), 6.36 (t, *J* = 6.0 Hz, 1H), 5.42 (s, 2H), 4.88 (s, 2H); ^13^C-NMR (100 MHz, CDCl_3_) δ 154.9, 153.8, 149.7, 141.0, 140.8, 139.6, 134.7, 133.0, 130.1, 128.3, 127.8 (×2), 125.9, 119.8, 118.4, 112.6, 46.8, 44.1. HRMS (ESI+) *m*/*z* calcd. for C_20_H_16_ClN_6_ [M + H]^+^ 375.1119, found 375.1119.

*4-Chloro-1-(4-nitrobenzyl)-1H-pyrazolo[3,4-d]pyrimidine* (**38a**). A mixture of 4-chloropyrazolo[3,4-d]pyrimidine (**37**, 155 mg, 1.00 mmol), 4-nitrobenzyl bromide (259 mg, 1.20 mmol) and Et_3_N (0.14 mL, 1.00 mmol) in DMF (10 mL) was allowed to stir at rt for 24 h. The volume of the reaction mixture was reduced to about 3 mL and water (30 mL) was added while stirred. The precipitate was filtered, washed with water and dried. The resulting residue was purified by flash column chromatography (5%–60% and then 80% EtOAc/hexanes) to afford compound **38a** as a pale solid (77 mg, 26%). ^1^H-NMR (400 MHz, DMSO-*d*_6_) δ 8.92 (s, 1H), 8.58 (s, 1H), 8.18 (d, *J* = 8.6 Hz, 2H), 7.50 (d, *J* = 8.6 Hz, 2H), 5.88 (s, 2H); ^13^C-NMR (100 MHz, DMSO-*d*_6_) δ 155.1, 153.9, 153.1, 147.0, 143.7, 133.1, 128.8, 123.8, 113.2, 49.9. HRMS (ESI^−^) *m*/*z* calcd. for C_12_H_7_ClN_5_O_2_ [M − H]^−^ 288.0294, found 288.0298.

*4-((4-Chloro-1H-pyrazolo[3,4-d]pyrimidin-1-yl)methyl)benzonitrile* (**38b**). Compound **38b** was prepared from 4-chloropyrazolo[3,4-d]pyrimidine (**37**, 155 mg, 1.00 mmol) and 4-cyanobenzyl bromide (235 mg, 1.20 mmol) in a fashion similar to the one described for compound **38a**. White solid, 69 mg, yield 26%. ^1^H-NMR (400 MHz, DMSO-*d*_6_) δ 8.91 (s, 1H), 8.56 (s, 1H), 7.80 (d, *J* = 8.6 Hz, 2H), 7.42 (d, *J* = 8.6 Hz, 2H), 5.82 (s, 2H); ^13^C-NMR (100 MHz, DMSO-*d*_6_) δ 155.0, 153.9, 153.1, 141.8, 133.0, 132.6, 128.5, 118.5, 113.2, 110.7, 50.1. HRMS (ESI^−^) *m*/*z* calcd. for C_13_H_7_ClN_5_ [M − H]^−^ 268.0395, found 268.0405.

*N-(3-Chlorobenzyl)-1-(4-nitrobenzyl)-1H-pyrazolo[3,4-d]pyrimidin-4-amine* (**31**). Compound **31** was prepared from **38a** (52 mg, 0.18 mmol) and 3-chlorobenzylamine (88 µL, 0.72 mmol) in a fashion similar to the one described for compound **3**. Tan solid, 70 mg, yield 98%. ^1^H-NMR (400 MHz, CDCl_3_) δ 8.44 (s, 1H), 8.16 (d, *J* = 8.6 Hz, 2H), 7.89 (s, 1H), 7.46 (d, *J* = 8.6 Hz, 2H), 7.37 (s, 1H), 7.30–7.27 (m, 3H), 5.84 (s, 1H), 5.67 (s, 2H), 4.85 (d, *J* = 6.0 Hz, 2H); ^13^C-NMR (100 MHz, CDCl_3_) δ 156.5, 153.8, 147.7, 143.7, 139.7, 135.0, 131.3, 130.4, 128.9, 128.2, 128.0, 127.8, 126.0, 125.9, 124.1, 50.1, 44.5. HRMS (ESI^+^) *m*/*z* calcd. for C_19_H_16_ClN_6_O_2_ [M + H]^+^ 395.0995, found 395.0996.

*4-((4-((3-Chlorobenzyl)amino)-1H-pyrazolo[3,4-d]pyrimidin-1-yl)methyl)benzonitrile* (**32**). Compound **32** was prepared from **38b** (49 mg, 0.18 mmol) and 3-chlorobenzylamine (88 µL, 0.72 mmol) in a fashion similar to the one described for compound **3**. Pale solid, 65 mg, yield 95%. ^1^H-NMR (400 MHz, CDCl_3_) δ 8.43 (s, 1H), 7.88 (s, 1H), 7.59 (d, *J* = 8.6 Hz, 2H), 7.39 (d, *J* = 8.6 Hz, 2H), 7.37 (s, 1H), 7.30–7.27 (m, 3H), 5.79 (s, 1H), 5.67 (s, 2H), 4.84 (d, *J* = 6.0 Hz, 2H); ^13^C-NMR (100 MHz, CDCl_3_) δ 156.4, 153.8, 147.5, 141.8, 139.8, 135.0, 132.7, 130.3, 128.7, 128.2, 128.0, 127.8, 126.0, 125.8, 118.7, 112.0, 50.4, 45.1. HRMS (ESI^+^) *m*/*z* calcd. for C_20_H_16_ClN_6_ [M + H]^+^ 375.1119, found 375.1113.

### 4.3. Cell Lines and Virus Stocks

The hepatocyte-derived cellular carcinoma cell line Huh7 was used for ZIKV and DENV studies. Huh7 cells were maintained in Minimum Essential Medium (MEM) supplemented with 10% fetal bovine serum (FBS), 100 IU streptomycin/penicillin per mL, 1 mM sodium pyruvate, 1× non-essential amino acids, 1× Glutamax (Gibco Life Technologies, Grand Island, NY, USA) and 10 μg/mL plasmocin (InvivoGen, San Diego, CA, USA). Vero (African green monkey kidney) cells were used to perform plaque assays for all viruses in this study. Vero cells were maintained in Dulbeco’s Modified Eagle Medium (DMEM) supplemented with 10% FBS, 100 IU streptomycin/penicillin per mL and 10 μg/mL plasmocin (InvivoGen). DENV-2 New Guinea C strain (ATCC VR-1584) and ZIKV Asian-American strain H/PAN/2015/CDC-259359 (ATCC VR-1859) stocks were generated in C6/36 mosquito cell cultures (ATCC CRL-1660) as indicated [26].

### 4.4. Antiviral Assays

#### 4.4.1. ZIKV Reporter Assay

The initial screening for ZIKV was performed using the in-house developed infectious ZIKV reporter expressing Nanoluc (ZIKV PAN1 Nluc) (see details in SI). Briefly, 1.5 × 10^4^ Huh7 cells per well were seeded in 96 well plates. Next day, cells were inoculated with the ZIKV reporter at a multiplicity of infection (MOI) of 0.2 in infection medium (MEM medium supplemented with 1% FBS, 100 IU streptomycin/penicillin per mL, 1 mM sodium pyruvate and 10 mM HEPES). After 2 h, the inoculum was replaced with post-infection medium (MEM medium supplemented with 5% FBS, 100 IU streptomycin/penicillin per mL, 1 mM sodium pyruvate and 10 mM HEPES) containing compound of interest (10 µM). We used the flavivirus NS5 MTase inhibitor NSC12155^13^ (10 µM) and the NS5 RdRp inhibitor NITD008^12^ (1 µM) as positive controls for inhibition or 0.05% DMSO as vehicle control. Seventy-two hours post-infection (hpi), the cells were lysed with 50 µL 1× luciferase lysis buffer (Promega, Madison, WI, USA), incubated 10 min at room temp and stored at −80 °C. Thirty-five µL of lysed cells were transferred to a white 96-well plate and combined with 35 µL Nano-Glo^®^ luciferase assay substrate (Promega) diluted 1:50 in Nano-Glo^®^ luciferase assay buffer (Promega). The reaction was incubated for 2 min and luminescence readings were acquired using a Neo 2 plate reader (BioTek, Winooski, VT, USA). Percent inhibition was calculated in GraphPad Prism (San Diego, CA, USA) normalizing the luciferase signal to infected cells treated with DMSO (0% inhibition) and uninfected cells treated with DMSO (100% inhibition). EC_50_ values were calculated in GraphPad Prism using data obtained from dose-response experiments. For compounds **1** and **8**, a range of concentrations between 0.05 and 15 µM were used. For compounds **11**, **14**, **23** and NSC12155, we used concentrations from 0.005 to 90 µM and 0.002 to 45 µM for NITD008.

#### 4.4.2. ZIKV and DENV Titer-Reduction Assay

Selected compounds were validated at 8.5 µM or 1.5 µM (for compounds considered toxic at higher concentrations) using titer-reduction assays. Huh7 cells (1.5 × 10^5^ per well) were plated in 24-well plates. Next day, the cells were inoculated with ZIKV or DENV (MOI 0.2 or 0.05, respectively) and two hours later, the inoculum was replaced with post-infection medium containing compound of interest at 8.5 µM or 1.5 µM. Seventy-two hpi, supernatants from infected and treated cells were collected and viral titers were measured by plaque assay. To visualize plaques, confluent Vero cells in 24-well plates were inoculated with 1:10 serial dilutions of supernatants. After two hours, the inoculum was retired and replaced with 800 µL of overlay medium (MEM medium containing 1.3% methylcellulose, 2% FBS, 100 IU streptomycin/penicillin per mL and 10 mM HEPES). After 5 days (7 days for DENV), cells were washed with PBS, fixed with 2% formaldehyde, and stained using 0.5% crystal violet in 25% methanol. Infectious virus titer (pfu/mL) was determined using the following formula: number of plaques × dilution factor × (1/inoculum volume (mL)). Viral titers were normalized with DMSO-treated and infected cells (0% inhibition). The limit of detection was 200 pfu/mL (100% inhibition). For NITD008, we assigned 100% inhibition because we did not detect any plaque at any dilution tested. Dose-response assays (Figure S4, Panels B and C) were performed for selected compounds using titer-reduction assays. We used a concentration range of 0.5 to 15 µM for all selected compounds except for compound 14 (range of 0.1–3 µM). Pfu/mL were normalized as mentioned for single-dose response experiment using the values for DMSO-treated and infected cells (0% inhibition) and the limit of detection value (200 pfu/mL) as 100% inhibition. Dose-response curves were built in GraphPad Prism and EC_50_ values were obtained from those curves.

### 4.5. Cell Viability Assay

Cell viability assays for non-infected Huh7 cells treated with the testing compounds (in-house library), control compounds (NITD008, RDV) and DMSO were performed in parallel with the antiviral screening. After 3 or 5 days of treatment, depending upon the experiment, cell viability was evaluated using the MTS-based tetrazolium reduction CellTiter 96 Aqueous Non-Radioactive cell proliferation assay (Promega). Treatment was retired from the wells and the cells incubated with MTS diluted 1:10 in MEM medium supplemented with 10% FBS. Absorbance was measured at 490 nm wavelength. Readings were normalized with DMSO-treated cells (100%) and expressed as % viability. For dose-response experiments, we used concentrations between 0.01 and 90 µM and dose-response curves and CC_50_ were calculated using GraphPad Prism.

## Figures and Tables

**Table 1:**
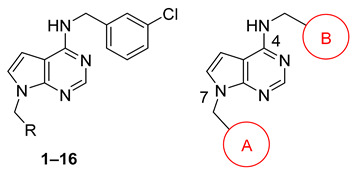
SAR study on ring A of the 7*H*-pyrrolo[2,3-d]pyrimidine scaffold against ZIKV.

Compd.	R	ZIKV Reporter Assay			Titer Reduction Assay					CC_50_ (µM)
		Inhibition (%) at 10 µM	Viability (%) at 10 µM	EC_50_ (µM)	% at 8.5 µM	% at 1.5 µM	EC_50_ (µM)	EC_90_ (µM)	EC_99_ (µM)	
**1**	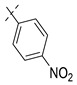	73	69	5.25	93		5.21	10.7	NR ^a^	20.0
**2**	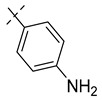	72	38			toxic				
**3**	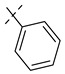	42	90		68					
**4**	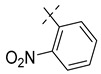	44	93		82					
**5**	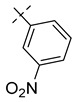	77	79		92					
**6**	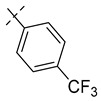	85	62		83					
**7**	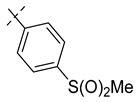	98	39			69				
**8**	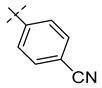	78	68	4.10	97	47	4.29	9.02	13.6	20.6
**9**	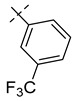	92	61		toxic					
**10**	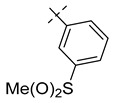	72	82		92					
**11**	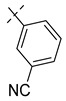	90	60	3.16	99		5.05	6.98	12.7	15.3
**12**	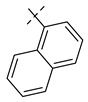	84	73		66					
**13**	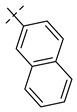	75	45			18				
**14**	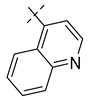	100	48	0.93		94	1.10	2.28	NR ^a^	5.67
**15**	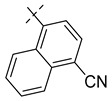	69	84		0					39.6
**16**	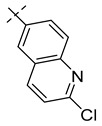	95	34			10				
DMSO		0	100		0	0				
NITD008 (1 µM)		100	100	0.14	100 (ND ^b^)					>45
NSC 12155		68	70	2.18	72	37	7.34	14.1	NR ^a^	23.0

^a^ NR, not reached; ^b^ ND, no plaque detected.

**Table 2:**
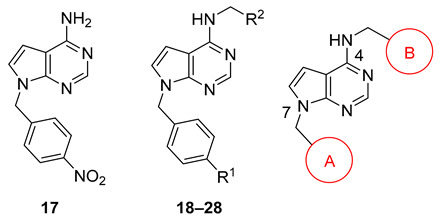
SAR study on ring B of the 7*H*-pyrrolo[2,3-d]pyrimidine scaffold against ZIKV.

Compd.	R^1^	R^2^	ZIKV Reporter Assay			Titer Reduction Assay					CC_50_ (µM)
			Inhibition (%) at 10 µM	Viability (%) at 10 µM	EC_50_ (µM)	% at 8.5 µM	% at 1.5 µM	EC_50_ (µM)	EC_90_ (µM)	EC_99_ (µM)	
**17**	NA ^a^	NA ^a^	0	92							
**18**	NO_2_	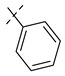	96	42							
**19**	NO_2_	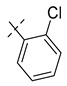	49	80		91					
**20**	NO_2_	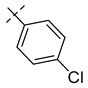	93	49			37				
**21**	NO_2_	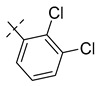	67	77		91					
**22**	NO_2_	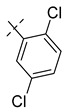	71	62		77					26.9
**23** ^b^	NO_2_	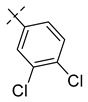	91	80	5.70	98		5.91	10.4	12.0	26.8
**24**	NO_2_	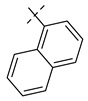	94	44			13				
**25**	CN	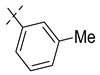	84	33							
**26**	CN	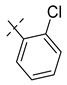	58	76							
**27**	CN	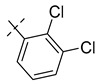	71	78							
**28**	CN	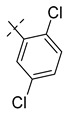	74	48							

^a^ NA, not applicable; ^b^ EC_99.9_ = 12.3 µM in the titer reduction assay.

**Table 3:**
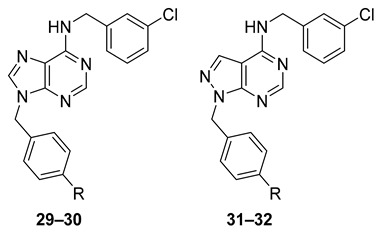
SAR study on the scaffold replacement against ZIKV.

Compd.	R	ZIKV Reporter Assay			Titer Reduction Assay					CC_50_ (µM)
		Inhibition (%) at 10 µM	Viability (%) at 10 µM	EC_50_ (µM)	% at 8.5 µM	% at 1.5 µM	EC_50_ (µM)	EC_90_ (µM)	EC_99_ (µM)	
**29**	NO_2_	59	87		90					24.5
**30**	CN	68	94		93		7.12	12.4	NR ^a^	49.3
**31**	NO_2_	83	96		86					
**32**	CN	79	88		88					17.4

^a^ NR, not reached.

## Supplementary Materials

The following are available online: Methods. Figure S1: Luciferase activity of serial dilutions of ZIKV PAN1 Nluc, Figure S2: The ^1^H and ^13^C chemical shifts of chloride **36a** and the HMBC correlations observed, Figure S3: The ^1^H and ^13^C chemical shifts of compound **29** and the HMBC correlations observed, Figure S4: Dose-response curves. Table S1: Primers and DNA sequences for pBAC ZIKV PAN1 NLuc construction Table S2: Sequence changes for pBAC-PAN1. NMR Spectra.

## Sample Availability

Samples of the compounds are available from the authors.

## Abbreviations

ABPP, activity-based protein profiling; DENV, Dengue virus; FDA, Food and Drug Administration; HCV, hepatitis C virus; HMBC, heteronuclear multiple bond correlation spectroscopy; HMQC, heteronuclear multiple quantum correlation; JEV, Japanese encephalitis virus; MOI, multiplicity of infection; MTase, methyltransferase; RdRp, RNA-dependent RNA polymerase; SAR, structure-activity relationship; WHO, World Health Organization; WNV, West Nile virus; YFV, Yellow fever virus; ZIKV, Zika virus.
