# Supplementary material for: 4,7-Disubstituted 7H-Pyrrolo[2,3-d]pyrimidines and Their Analogs as Antiviral Agents against Zika Virus
Source: Molecules. 2021 Jun 22;26(13):3779. doi: 10.3390/molecules26133779 (PMC8270260; doi:10.3390/molecules26133779)
Supplement: Supplementary file 1 [file molecules-26-03779-s001.zip › molecules-1257759-supplementary.pdf]

molecules

MDPI

Supporting Information

4,7-Disubstituted 7*H*-Pyrrolo[2,3-*d*]pyrimidines and Their Analogs as Antiviral Agents against Zika Virus

Ruben Soto-Acosta, Eunkyung Jung, Li Qiu, Daniel J. Wilson, Robert J. Geraghty* and Liqiang Chen*

Table of Contents

- Construction of ZIKA PAN NanoLuc: S1
- ZIKV RNA transcription and capping: S2
- Production of ZIKV PAN Nluc infectious virus: S2
- **Table S1.** Primers and DNA sequences for pBAC ZIKV PAN1 NLuc construction: S2
- **Table S2.** Sequence changes for pBAC-PAN1: S3
- **Figure S1.** Luciferase activity of serial dilutions of ZIKV PAN1 Nluc: S4
- **Figure S2.** The ^{1}H and ^{13}C chemical shifts of chloride **36a** and the HMBC correlations observed: S4
- **Figure S3.** The ^{1}H and ^{13}C chemical shifts of compound **29** and the HMBC correlations observed: S4
- **Figure S4.** Dose-response curves: S5
- **NMR spectra**: S6

Construction of ZIKA PAN NanoLuc

A DNA clone of the ZIKV PAN genome was constructed by performing overlapping RT-PCR using purified infected cell RNA and then inserting the fragments in one step using NEBuilder HiFi DNA Assembly kit (New England Biolabs, Ipswich, MA, USA) into a low copy bacterial artificial chromosome (BAC), pBeloBAC (New England Biolabs). Vero cells were inoculated with ZIKV PAN at an MOI of 0.1 and after CPE was observed, total RNA was isolated using the QIAamp Viral RNA Mini Kit (Qiagen, Germantown, MD, USA). Two separate RT-PCR reactions were performed using 0.1 micrograms total RNA, one primed by random hexamers and the other with primers specific to the latter third of the viral genome (PAN3A_F and PAN3_3A1_R, see Table S1). The RT reactions were performed using MuLV RT (New England Biolabs) as per manufacturer's instructions. Four microliters of the cDNA were used for PCR with primer pair PAN1_F/PAN1_R for fragment 1, PAN2_F/PAN2_R for fragment two and PAN3_F/PAN3_R27B for fragment 3. The PCR products were gel purified and approximately 100 ng of each fragment was inserted into 250 ng pBeloBAC digested with ApaI in one reaction using NEBuilder. Approximately 2.5 µL of the reaction was transformed in *E. coli* and colonies screened via *XhoI/ScaI* double restriction enzyme digestion. Positive clones were sequenced using next generation sequencing (sequence available upon request) and one clone, pBACPAN1 was chosen as the DNA molecular clone for the ZIKV PAN genome. There were a few differences from the ZIKV PAN reference sequence KX156775 (Table S2).

After construction of the ZIKV PAN DNA molecular clone, further steps were needed to insert a ribozyme at the 3′ end to ensure appropriate processing and insertion of a reporter gene to estimate virus replication. Towards those ends, a DNA fragment was synthesized containing the hepatitis D virus ribozyme (HDvR) by IDT (Table S1). Primers HDvR_1 and 2 were used to amplify the HDvR from the fragment. Primer pairs HDvR_3 and _4 were used to amplify the 5′ sequence overlapping that of the pBACPAN1 insertion site and the HDvR fragment and the primer pair HDvR_5 and _6 were used to amplify

the 3′ sequence overlapping that of the pBACPAN1 insertion site and the HDvR fragment. All three fragments were inserted into pBACPAN1 digested with BstBI and XhoI using NEBuilder HiFi DNA assembly kit (New England Biolabs) as per manufacturer's instructions. The product, pBAC_PAN1_HDvR was sequenced using next generation methods and revealed an A to G change at 2699 compared to pBAC_PAN1 sequence (numbering as in Table S2, no amino acid change) and is available upon request.

The NanoLuc luciferase gene was inserted into the ZIKV genome DNA copy using a similar strategy to that used for the HDvR. A DNA fragment containing the NanoLuc sequence was synthesized by IDT (TableS1). The NanoLuc fragment was amplified with primers NanoLuc_1 and _2 while the pBACPAN1 was amplified with primers NanoLuc_3 and *4*. The PCR fragments were inserted into pBAC_PAN1_HDvR digested with *NheI* and *PmlI* using the NEBuilder HiFi DNA assembly kit (New England Biolabs) to yield pZIKV PAN1 NanoLuc.

ZIKV RNA transcription and capping

BAC DNA was isolated using the Large Construct Kit (Qiagen) using the manufacturer's protocol. Template for RNA production was amplified using the primers ZIKVRNA_1 and _2. Transcription and RNA capping were performed in a single reaction using the HiScribe T7 Quick High Yield RNA kit (New England Biolabs) and the cap analogue ARCA (3'-O-Mem7G(5')ppp(5')G) (New England Biolabs). Reactions were carried out per the manufacturer's instructions and contained 8 mM ARCA and 1 μg template in a 20 μL reaction. RNA was purified after DNase I treatment using the RNA Clean and Concentrator-25 kit (Zymo Research, Irvine, CA, USA) with a final yield of 87 μg.

Production of ZIKV PAN Nluc infectious virus

$1x10^6$ Vero cells were electroporated with 1.5 ug of capped ZIKV PAN1 Nluc RNA obtained by in vitro transcription/capping above. Electroporation performed using Neon transfection system (ThermoFisher Scientific, Waltham, MA, USA) per their instructions. After electroporation, cells were plated in one well of a 6-well dish in infection medium (DMEM, 5% FBS, 1× penicillin/streptomycin, 10mM HEPES). The cells were cultured at $37^oC/5\% CO_2$ until cytopathic effects were observed, approximately eight days after electroporation. Supernatant was collected and 1 mL diluted by 19 mL infection medium and used to inoculate a T175 cm^2 flask with Vero cells at 60-80% confluency. After six days, cytopathic effects were observed and virus-containing supernatant collected. Serial tenfold dilutions of virus stock was diluted and used to inoculate Vero cells in a 96-well plate. After 48 hours, the cells were lysed with Renilla luciferase lysis buffer and luciferase signal was measured using the Nano-Glo® system (Promega). The results are shown in Figure S1. The concentration of virus in the stock virus prep was also determined by plaque assay on Vero cells as described in the Experimental Section with the one change of extending the assay to seven days before staining for plaques. The titer of the stock virus was determined to be 6.5×10^6 plaque forming units per mL.

Table S1. Primers and DNA sequences for pBAC ZIKV PAN1 NLuc construction.

Primer/DNA Name	DNA Sequence 5′–3′
PAN1_F	GCATCAGAGCAGATTGTACTGAGAGTAATACGACTCACTATAGAGTTGTTG ATCTGTGTGAATCAGACTGCGAC
PAN1_R	AATTTCAAGCTCTTCACTGTGCCATGGCCCTTTCATTTGGGTC
PAN2_F	CATGGCACAGTGAAGAGCTTGAAATTCGGTTTGAGGAATGCCCAGGCACTA AGGTCC
PAN2_R	CTCCGGCAATTGGGCCGCCGCGGCTTTGTAAGGCCTGCTTCCA
PAN3_F	AAGCCGCGGCGGCCCAATTGCCGGAGACCCTAGAGACCATTATGCTTTTGG GGTTGC

PAN3_R27B	TCACACCGCATATGGGACCCATGGATTTCCCCACACCGGCCGCCGCTATTC<br>GGCGATCTGTGCCTG
PAN3A_F	ACCCTAGAGACCATTATGCTTTTGGGGTTGC
PAN3_3A1_R	GACCCATGGATTTCCCCACACCGGC
HDvR_1	CGGAAAACGCAAAACAGC
HDvR_2	ACATCCCCCTTTCGCCAG
HDvR_3	GGCTAGATTTCTAGAGTTCGAAG
HDvR_4	GCTGTTTTGCGTTTTCCG
HDvR_5	CTGGCGAAAGGGGGATGTAAGCTTGAGTATTCTATAG
HDvR_6	GTGCTTCCTCGCTCACTC
NanoLuc_1	TCGAGTTTGAAGCGAAAGCTAGCAACAGTATCAACAG
NanoLuc_2	GGGCCCAGGGTTGGACTC
NanoLuc_3	TCCAACCCTGGGCCCATGAAAAACCCAAAAAAGAAATCCGGAGGATTCCG<br>GATTGTGAACATGTTGAAACGCGGAGTAGCCCGTG
NanoLuc_4	CCACATGTTTCCTCCACGTGGAC
ZIKVRNA_1	TGAAAGCGAGCTTTTTGGCC
ZIKVRNA_2	TTCACACCGCATATGGGACC
HDvR fragment (HDvR underlined) [a]	ACTAGTGGTTAGAGGAGACCCCCCGGAAAACGCAAAACAGCATATTGACG<br>CTGGGAAAGACCAGAGACTCCATGAGTTTCCACCACGCTGGCCGCCAGGCA<br>CAGATCGCCGAATAGCGGCGGCCGGTGTGGGGAAATCCATGGGTCTGGGTC<br>GGCATGGCATCTCCACCTCCTCGCGGTCCGACCTGGGCTACTTCGGTAGGCT<br>AAGGGAGAAGAATCGATGGGTCCCATATGCGGTGTGAAATACCGCACAGA<br>TGCGTAAGGAGAAAATACCGCATCAGGCGCCATTCGCCATTCAGGCTGCGC<br>AACTGTTGGGAAGGGCGATCGGTGCGGGCCTCTTCGCTATTACGCCAGCTG<br>GCGAAAGGGGGATGTGCTGCAAGGCGATTAAGTTGGGTAACGCCAGGGTTT<br>TCCCAGTCACGACGTTGTAAAACGACGGCCAGTGAATTGTAATACGACTCA<br>CTATAGGGCGAATTCGAGCTCGGTACCCGGGGATCCTCTAGAGTCGACCTG<br>CAGGCATGCA
NanoLuc fragment (NanoLuc underlined, FMDV 2A sequence in italics) [b]	CTAGCAACAGTATCAACAGGTTTTATTTTGGATTTGGAAACGAGAGTTTCTG<br>GTCATGAAAAACCCAAAAAAGAAATCCGGAGGATTCCGGATTGTCAATAT<br>GCTAAAACGCGGAGTAGCCCGTGTGAGCATGGTCTTCACACTCGAAGATTT<br>CGTTGGGGACTGGCGACAGACAGCCGGCTACAACCTGGACCAAGTCCTTGA<br>ACAGGGAGGTGTGTCCAGTTTGTTTCAGAATCTCGGGGTGTCCGTAACTCCG<br>ATCCAAAGGATTGTCCTGAGCGGTGAAAATGGGCTGAAGATCGACATCCAT<br>GTCATCATCCCGTATGAAGGTCTGAGCGGCGACCAAATGGGCCAGATCGAA<br>AAAATTTTTAAGGTGGTGTACCCTGTGGATGATCATCACTTTAAGGTGATCC<br>TGCACTATGGCACACTGGTAATCGACGGGGTTACGCCGAACATGATCGACT<br>ATTTCGGACGGCCGTATGAAGGCATCGCCGTGTTCGACGGCAAAAAGATCA<br>CTGTAACAGGGACCCTGTGGAACGGCAACAAAATTATCGACGAGCGCCTG<br>ATCAACCCCGACGGCTCCCTGCTGTTCCGAGTAACCATCAACGGAGTGACC<br>GGCTGGCGGCTGTGCGAACGCATTCTGGCG*TTGAACTTTGACCTTCTCAAGTT*<br>*GGCTGGAGACGTGGAGTCCAACCCTGGGCCC*

[a]HDvR = hepatitis delta virus ribozyme.
[b]FMDV 2A = foot and mouth disease virus self-cleaving peptide 2A.

Table S2. Sequence changes for pBAC-PAN1.

Nucleotide [a]	PAN KS156775	BAC-PAN1	Amino Acid Change [b]
491	C	T	--
2555	T	C	--
2662	T	C	F to S (NS1)
2699	A	G	--
4319	G	A	M to I (NS2B)
7313	C	T	--

[a] The sequence for ZIKV PAN isolate (accession # KS156775) is used with the addition of the first 51 nucleotides of the BAC-PAN1 sequence. The added sequence is identical to most published

ZIKV 5′ end genomic sequences. The 5′ 51 nucleotides of ZIKV PAN KS156775 is missing from the submitted sequence. [b]Single letter amino acid abbreviations used. Changes are from ZIKV PAN KS156775 to BAC-PAN 1. The mature viral protein where residue change is predicted to occur is in parenthesis.

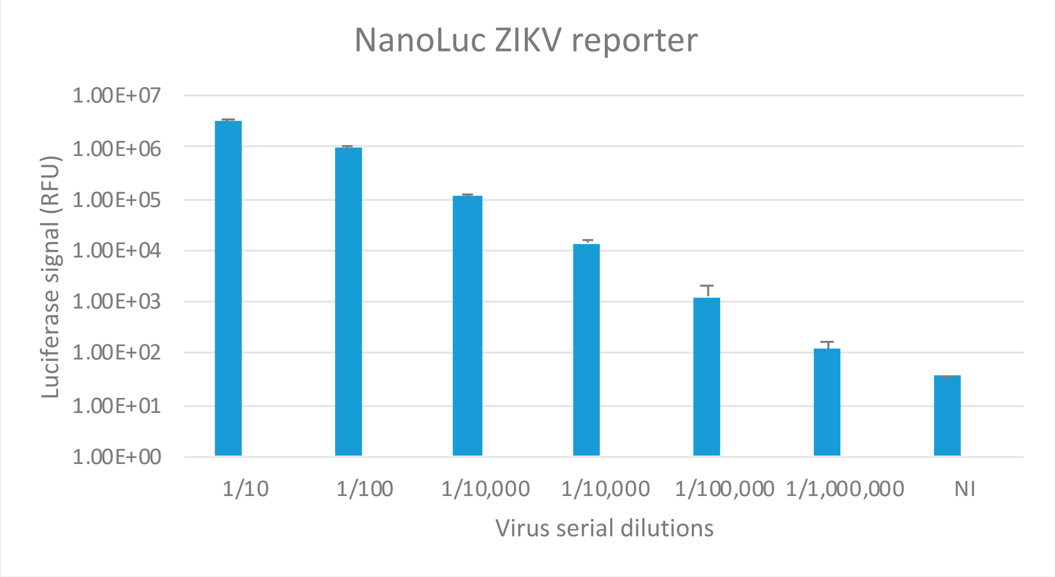

Figure S1. Luciferase activity of serial dilutions of ZIKV PAN1 Nluc. Ten-fold serial dilutions of stock virus prep were used to inoculate Vero cells in 96-well plates. Two days later, cultures were processed for luciferase activity. Inoculations performed in triplicate and mean values plus standard error shown. NI = no infection. RFU = relative fluorescence units.

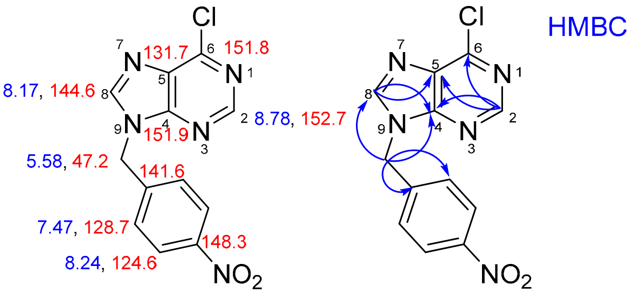

Figure S2. The ^{1}H and ^{13}C chemical shifts of chloride **36a** and the HMBC correlations observed.

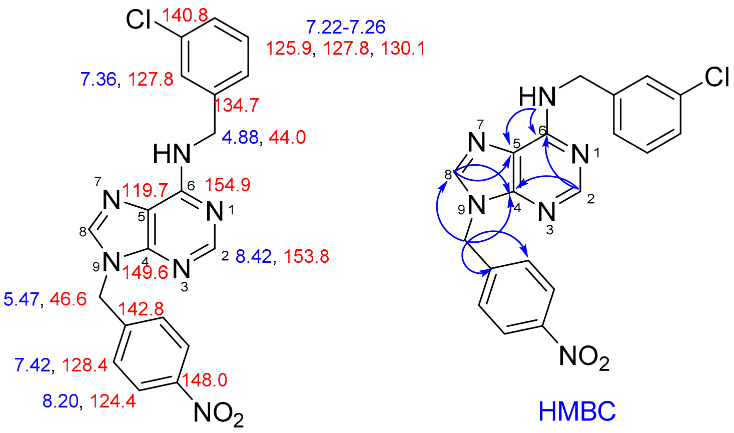

Figure S3. The ^{1}H and ^{13}C chemical shifts of compound **29** and the HMBC correlations observed.

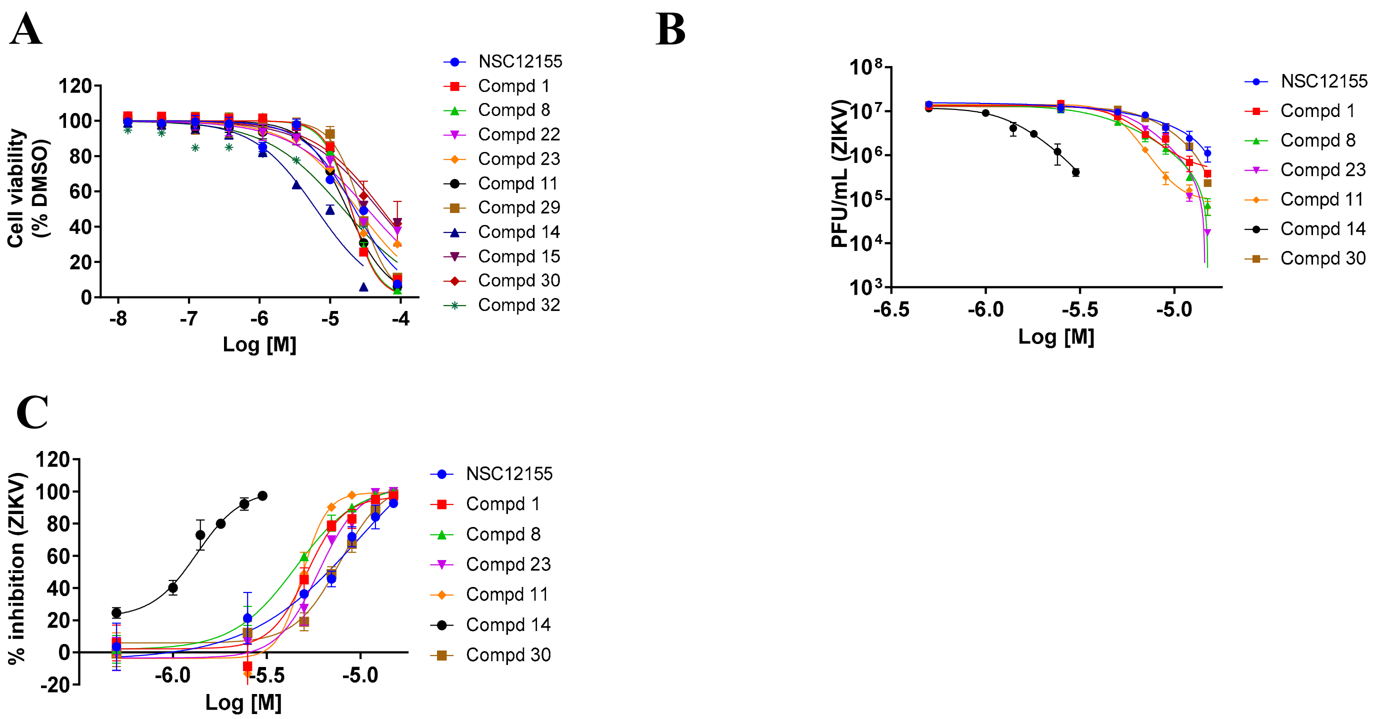

Figure S4. Dose-response curves. **A)** Cell viability assay. Huh7 cells were treated with selected compounds at different concentrations and incubated for 72 hours. After incubation, cell viability was examined by MTS assay. Cell viability curves and CC_{50} values were obtained in GraphPad Prism. **B)** Antiviral activity against ZIKV. Huh7 cells were inoculated with ZIKV at MOI of 0.2 and treated with selected compounds at different concentrations. After 72 hours, supernatants were collected and virus titer was measured by plaque assay. Pfu = plaque-forming units. **C)** Virus titer of ZIKV infected supernatants expressed as percent inhibition normalizing vs DMSO control (0% inhibition) and the limit of detection value (200 pfu/mL) as 100% inhibition.

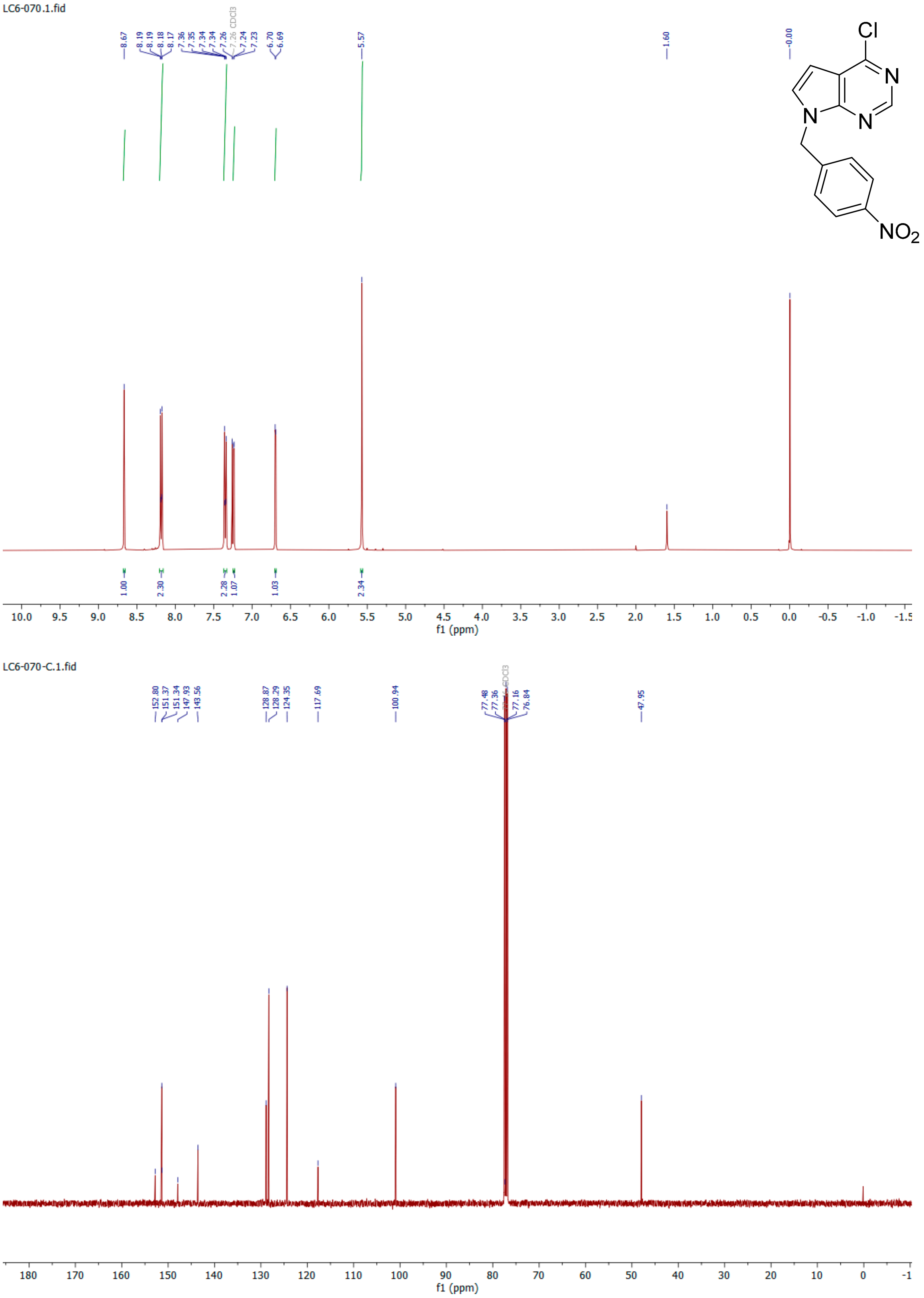

Figure S5. ^{1}H and ^{13}C spectra of chloride **34a**.

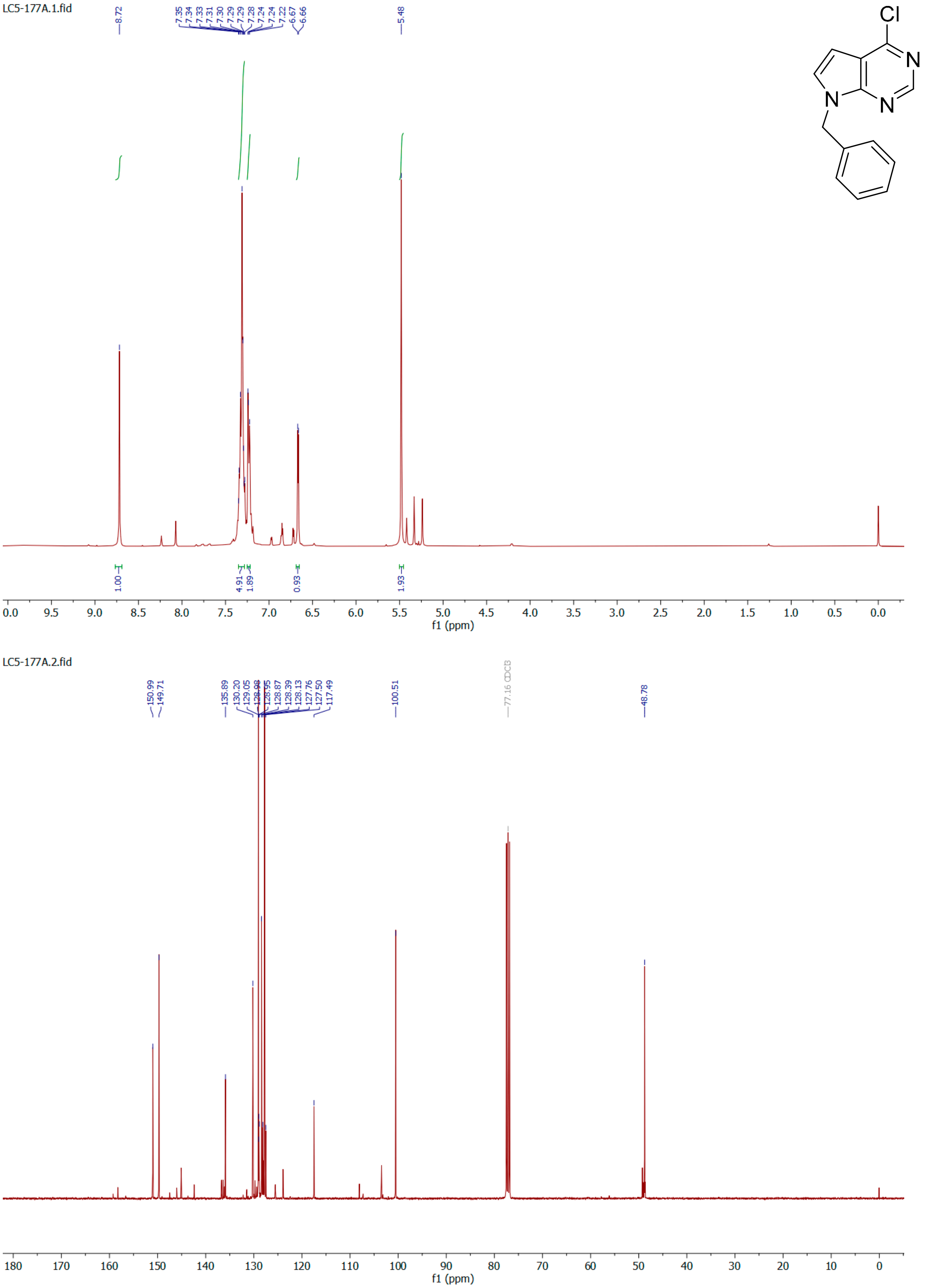

Figure S6. ^{1}H and ^{13}C spectra of chloride **34b**.

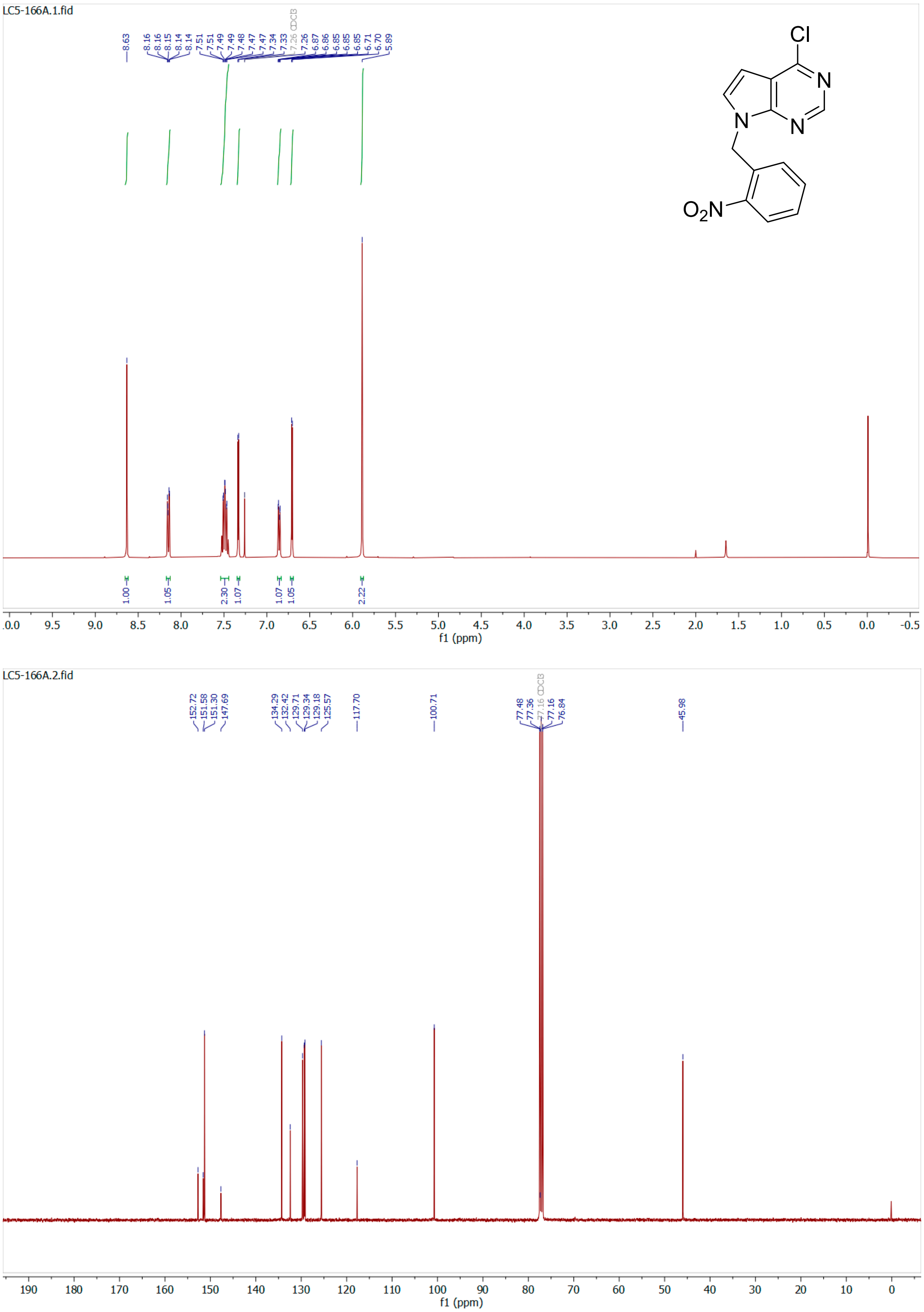

Figure S7. ¹H and ¹³C spectra of chloride **34c**.

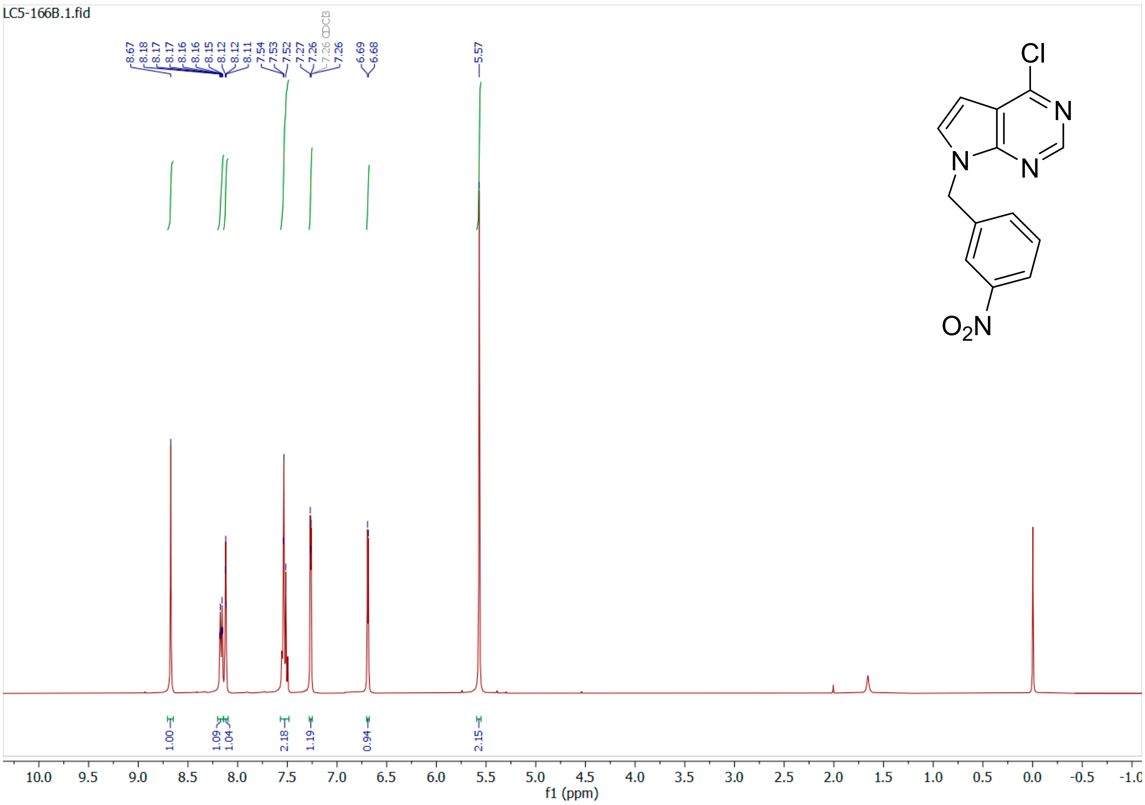

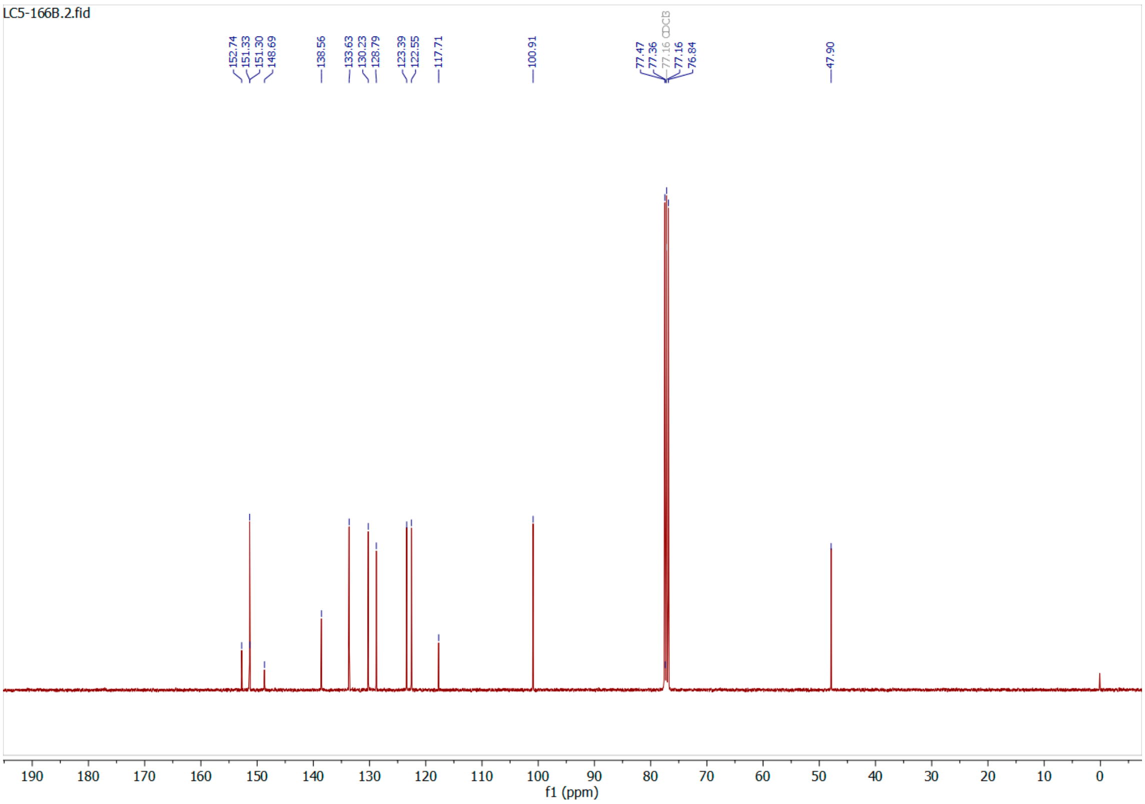

Figure S8. ^{1}H and ^{13}C spectra of chloride **34d**.

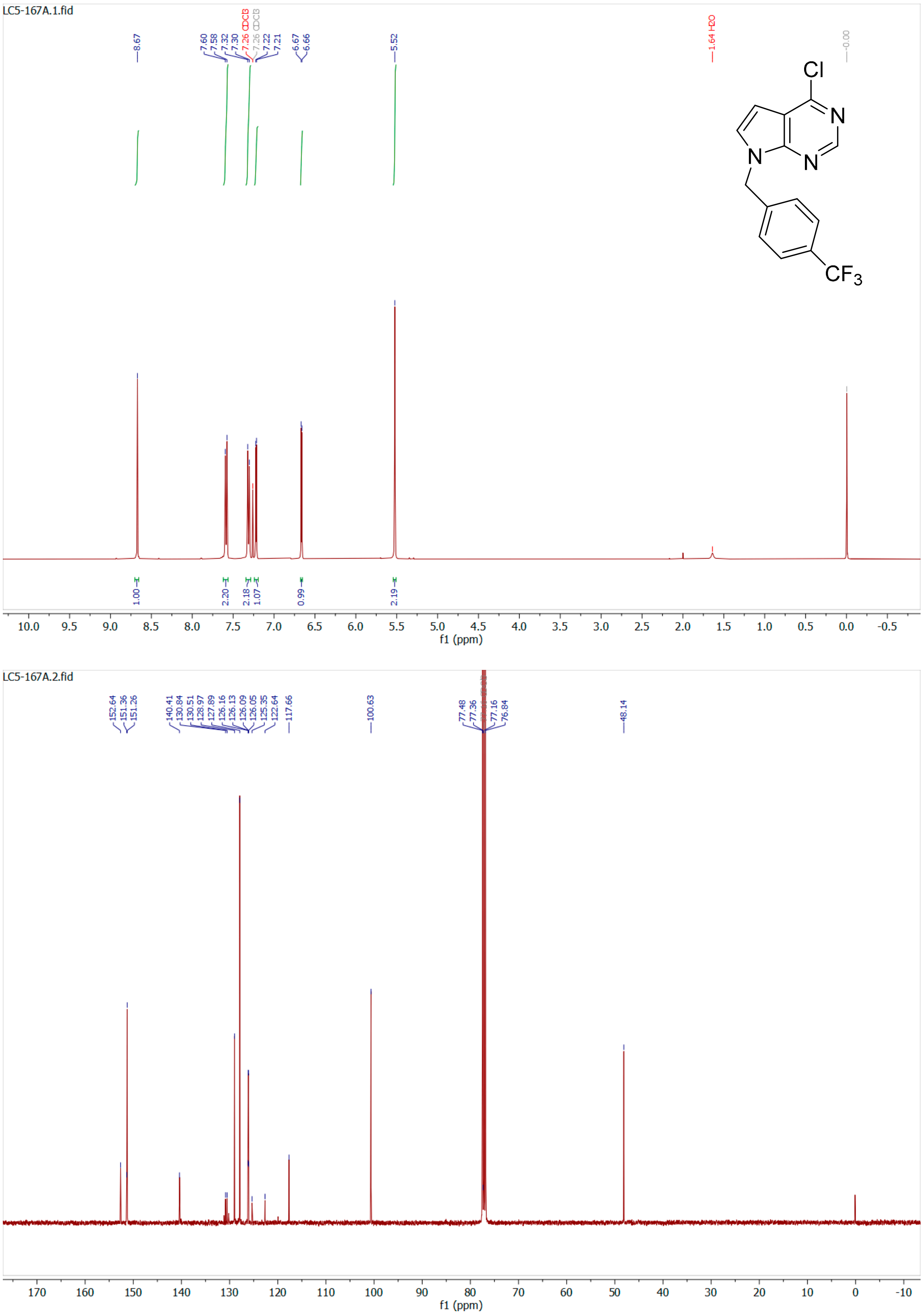

Figure S9. ^{1}H and ^{13}C spectra of chloride **34e**.

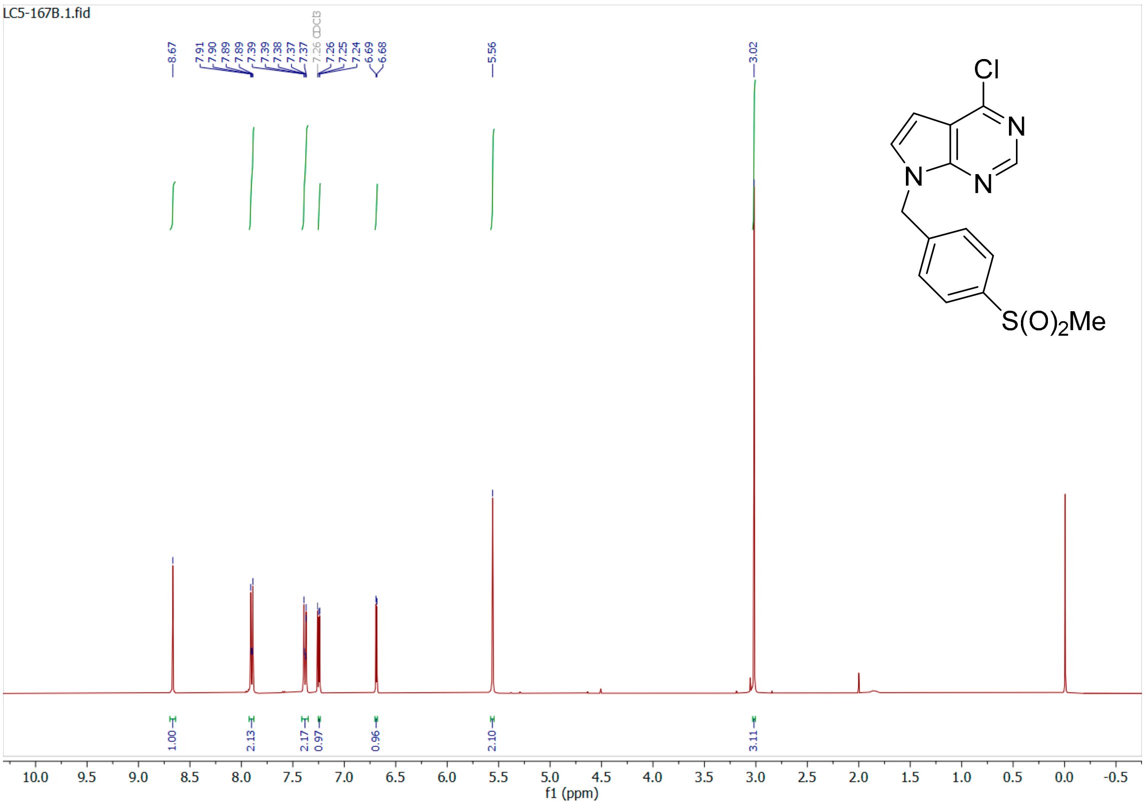

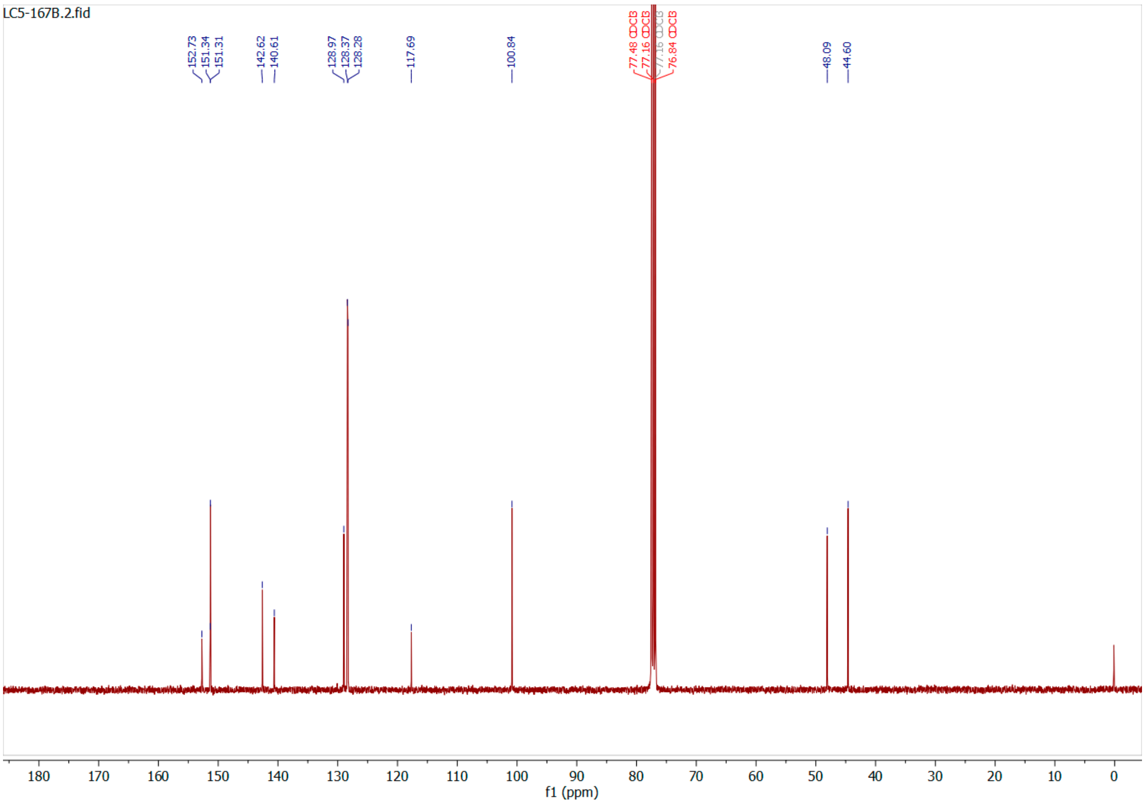

Figure S10. ^{1}H and ^{13}C spectra of chloride **34f**.

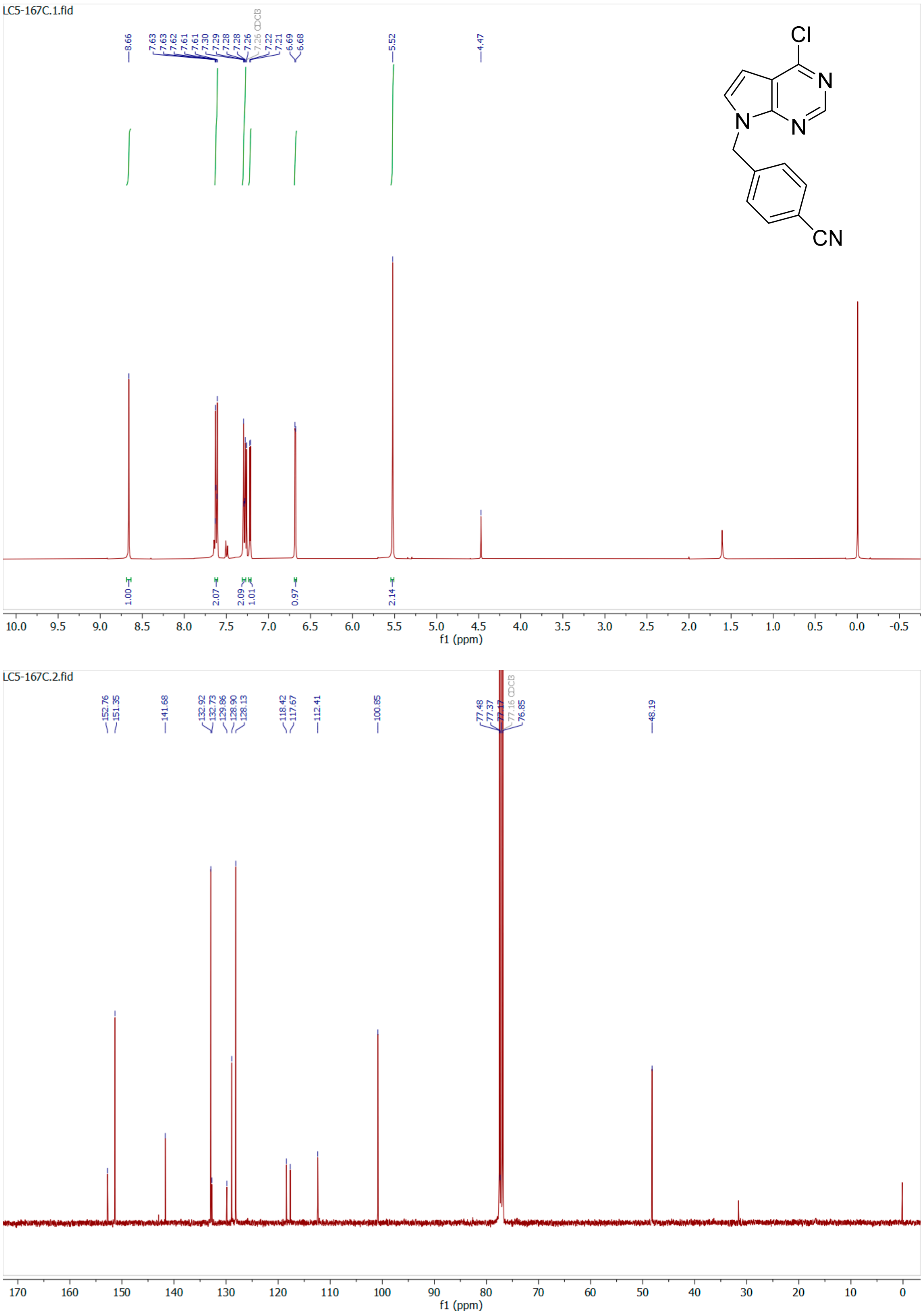

Figure S11. ^{1}H and ^{13}C spectra of chloride **34g**.

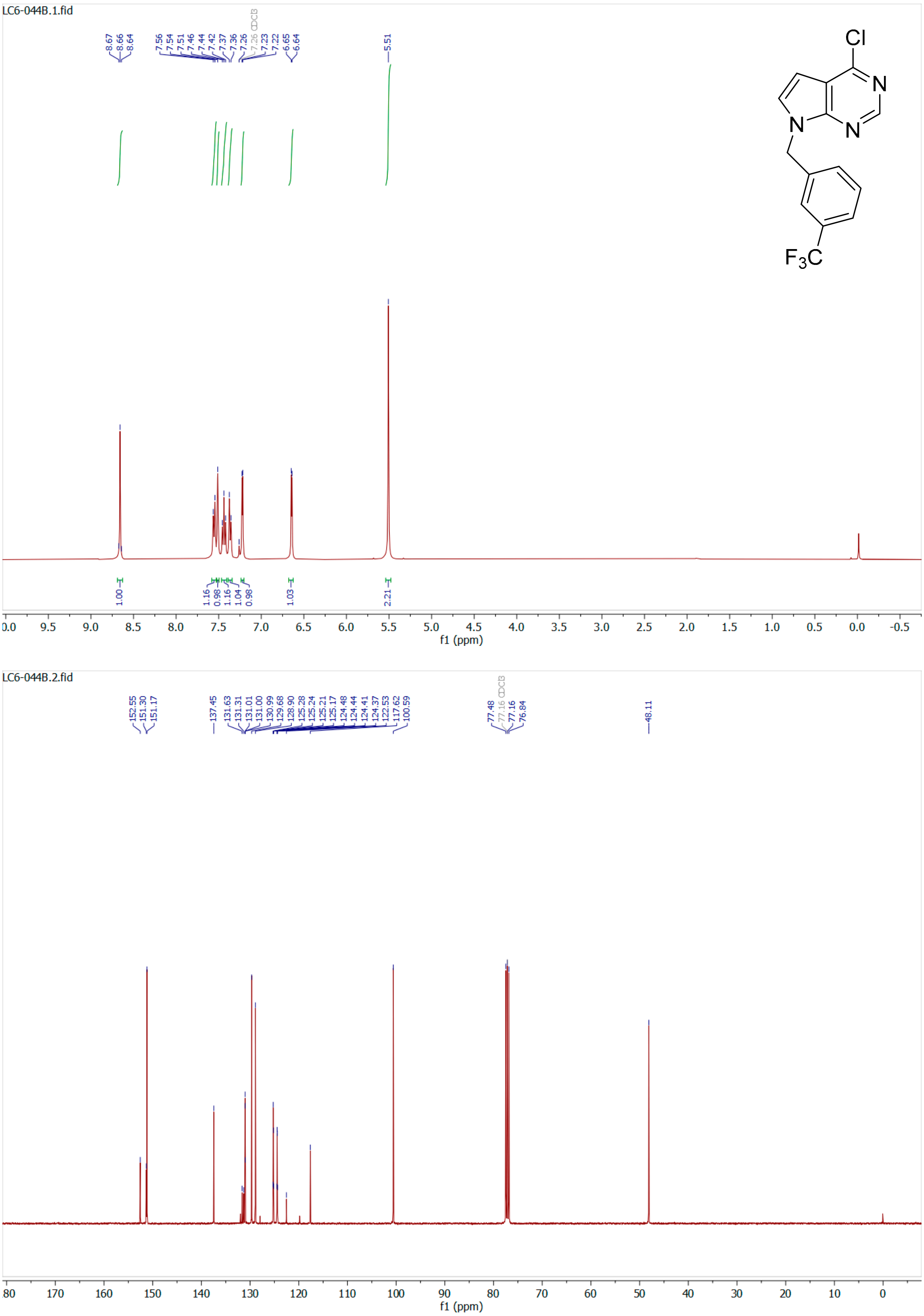

Figure S12. ^{1}H and ^{13}C spectra of chloride **34h**.

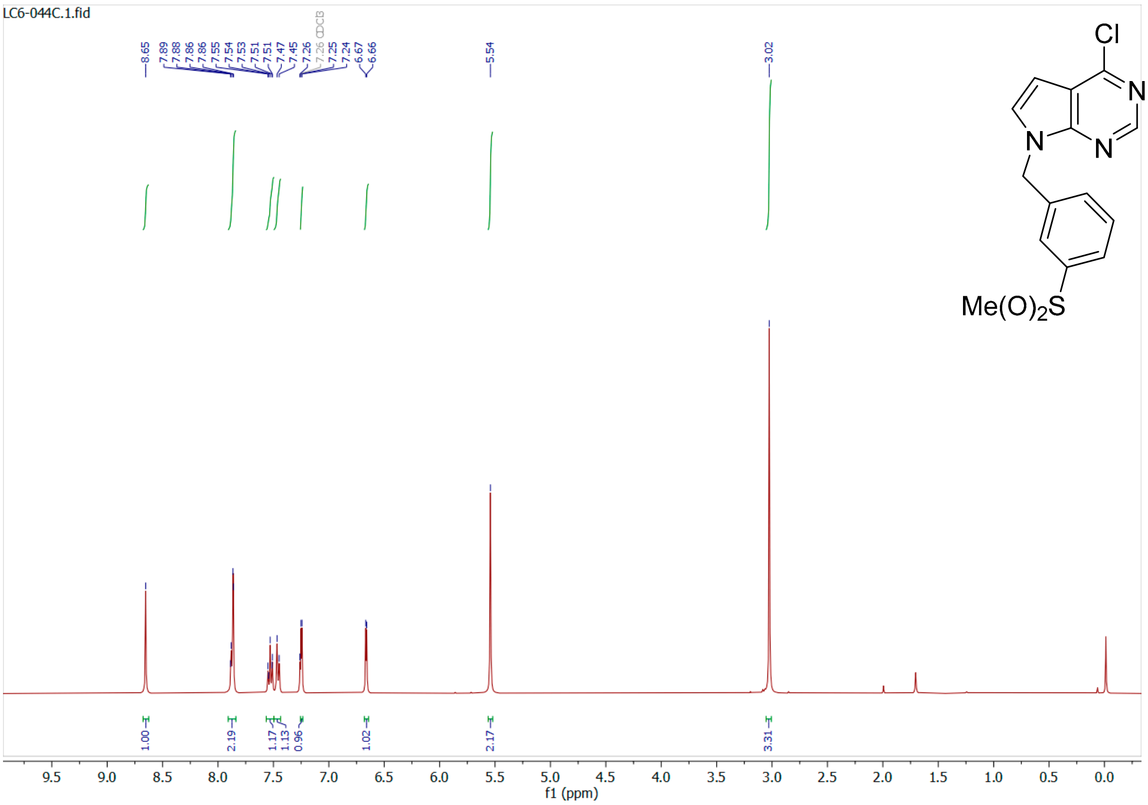

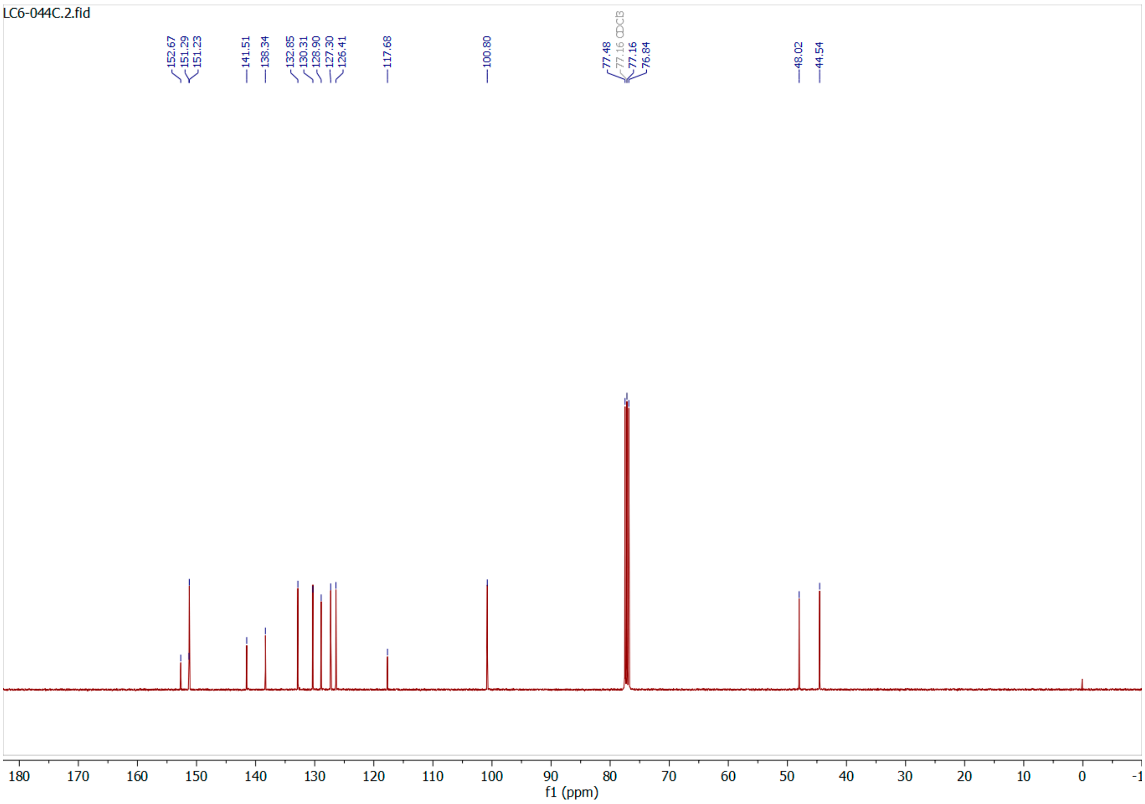

Figure S13. ^{1}H and ^{13}C spectra of chloride **34i**.

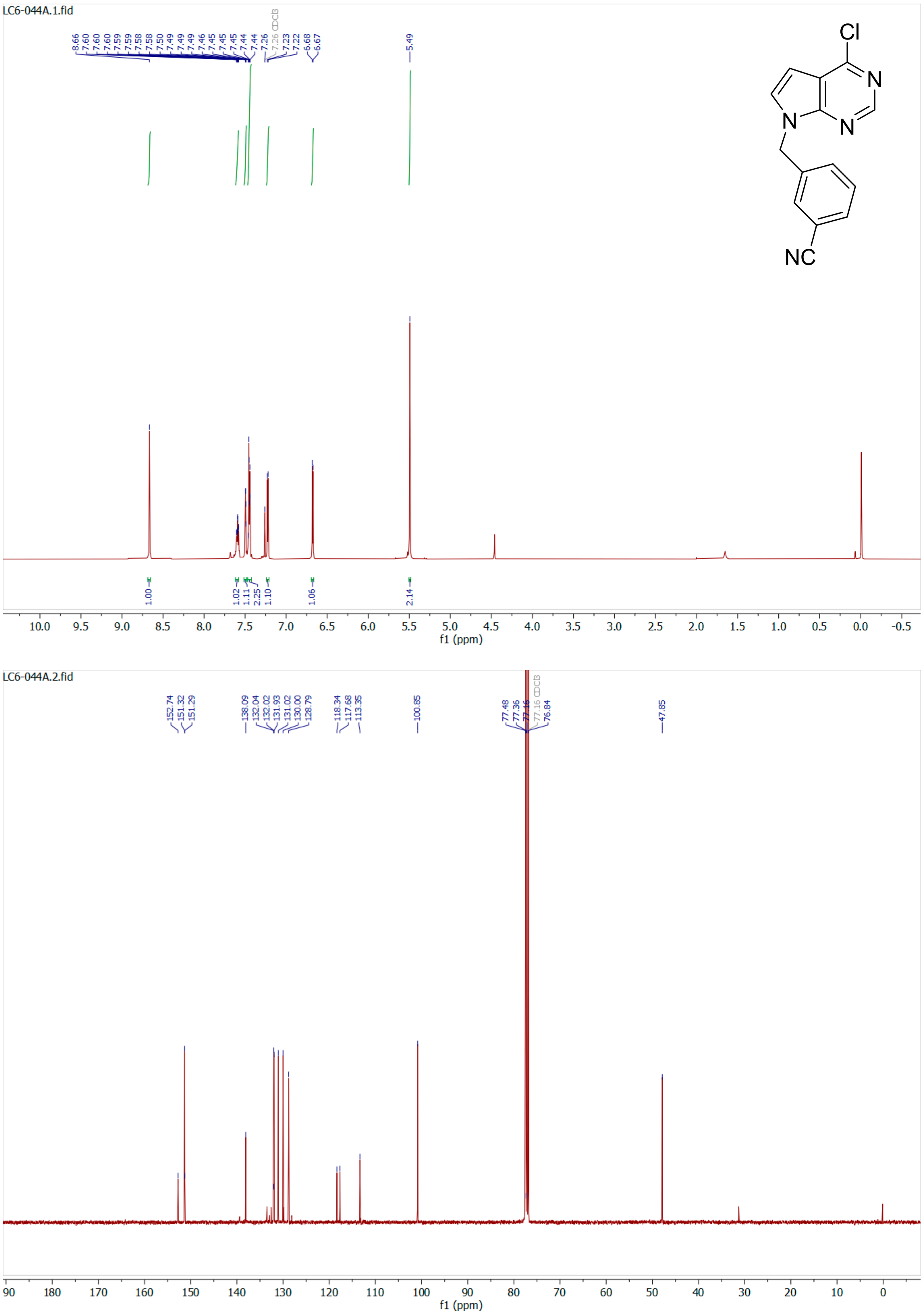

Figure S14. ^{1}H and ^{13}C spectra of chloride **34j**.

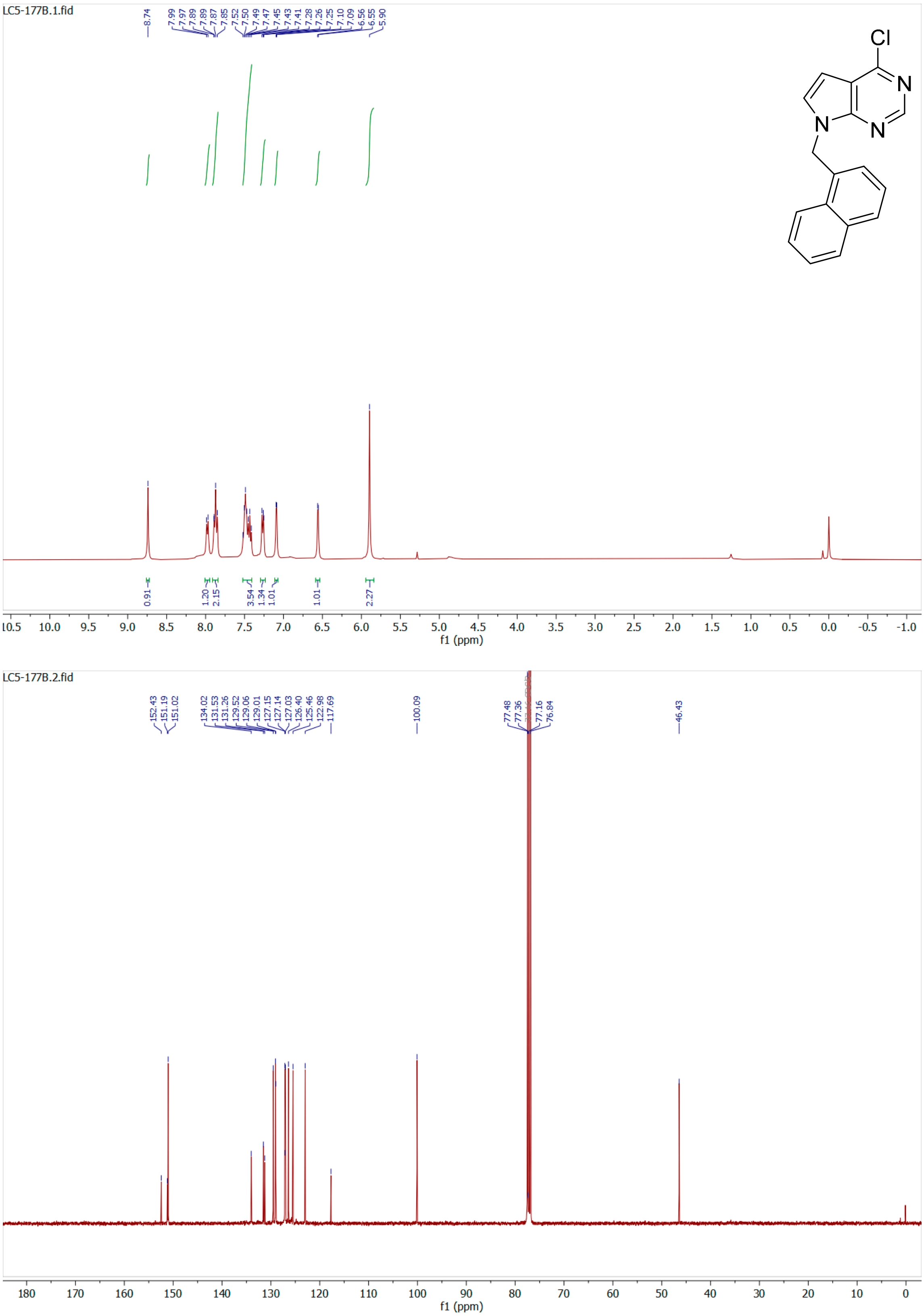

Figure S15. ^{1}H and ^{13}C spectra of chloride **34k**.

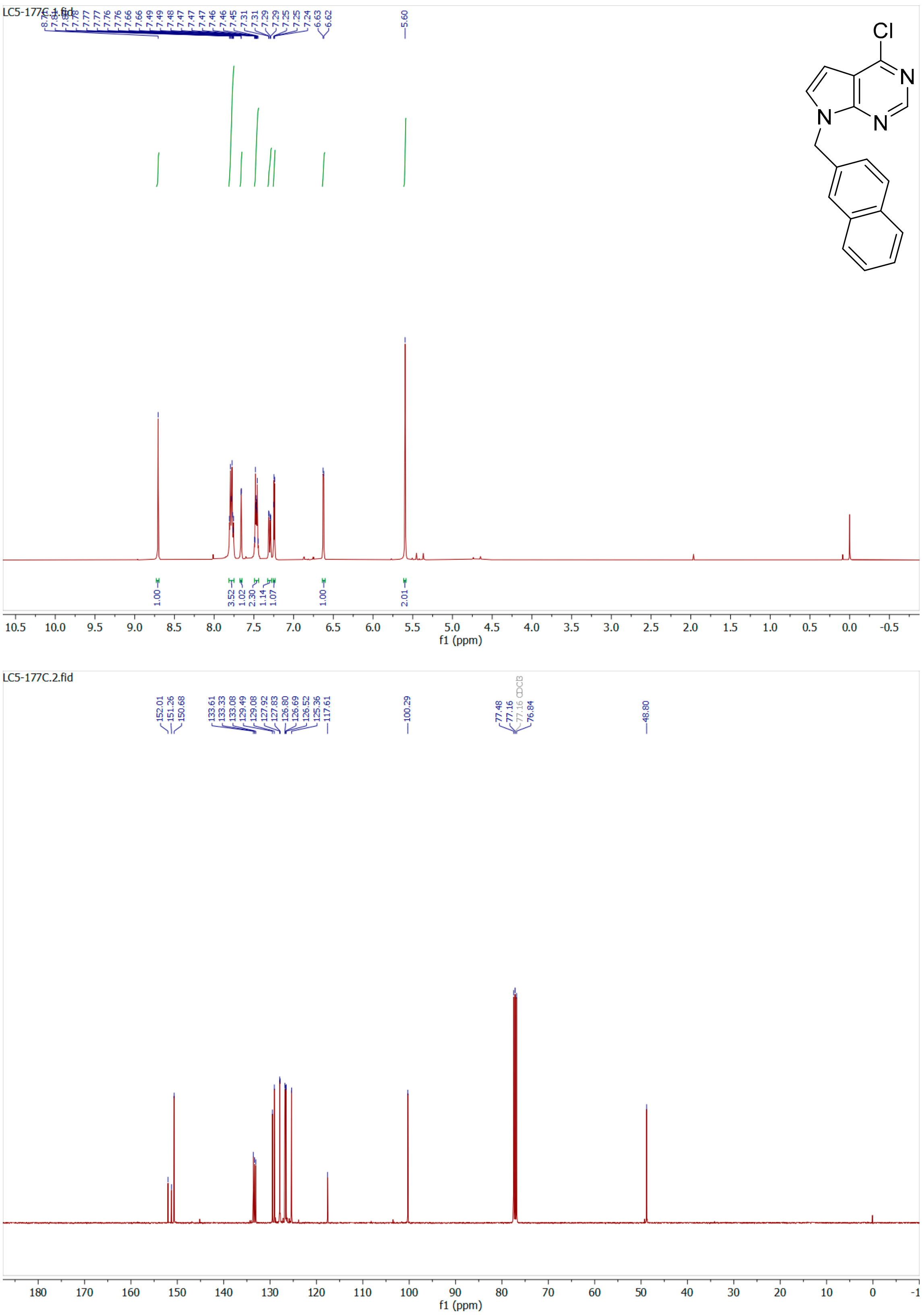

Figure S16. ^{1}H and ^{13}C spectra of chloride **34l**.

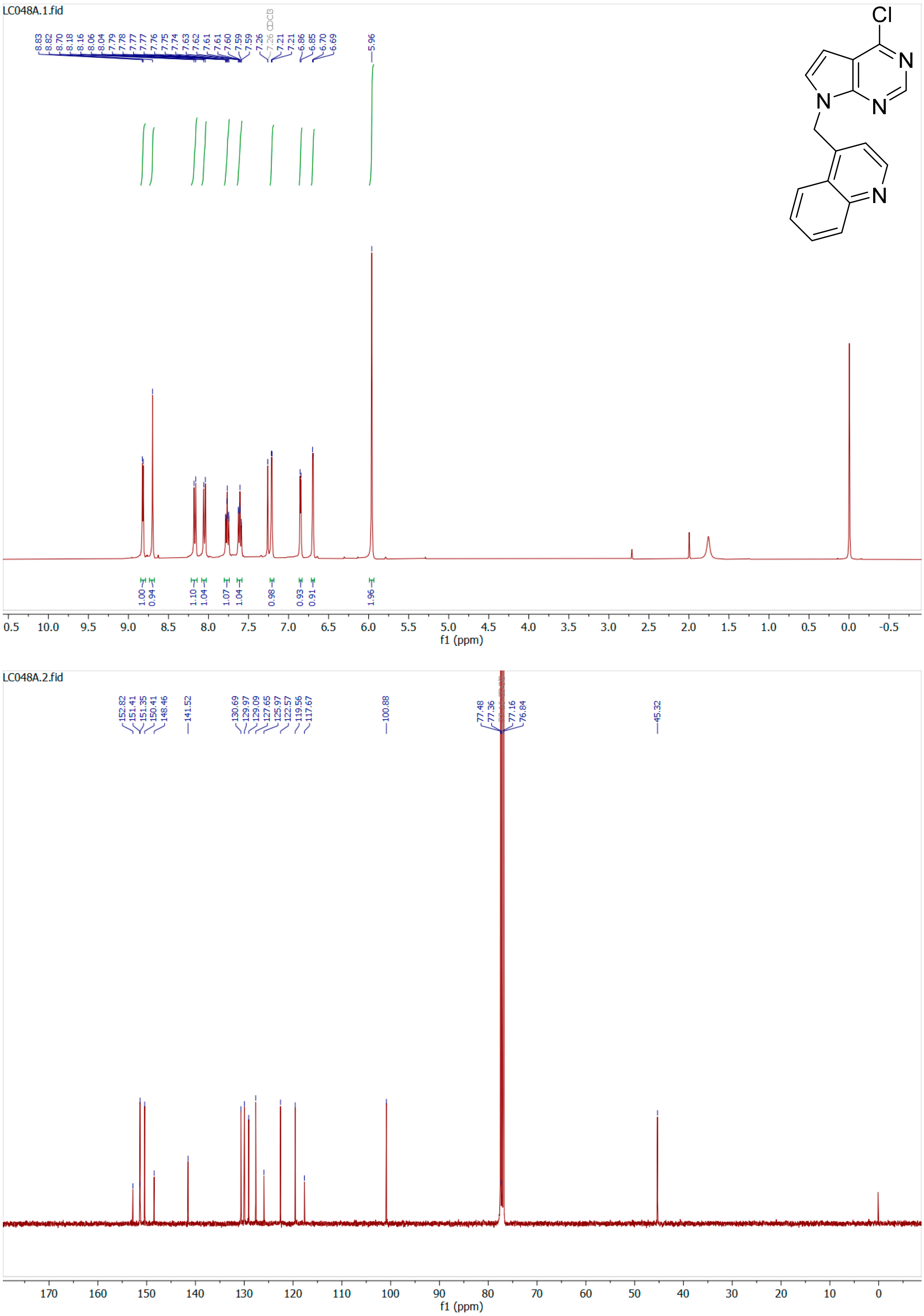

Figure S17. ^{1}H and ^{13}C spectra of chloride **34m**.

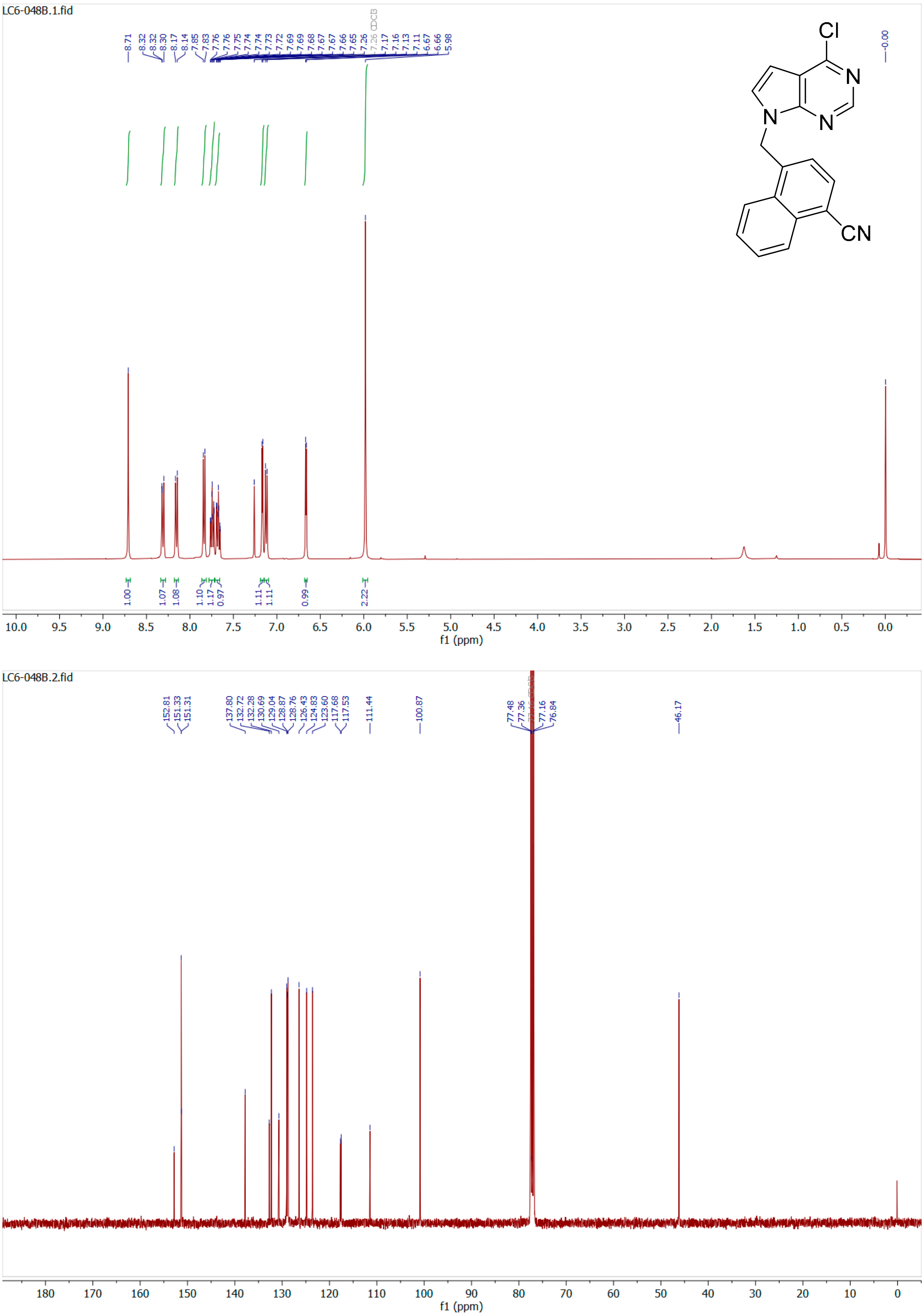

Figure S18. ^{1}H and ^{13}C spectra of chloride **34n**.

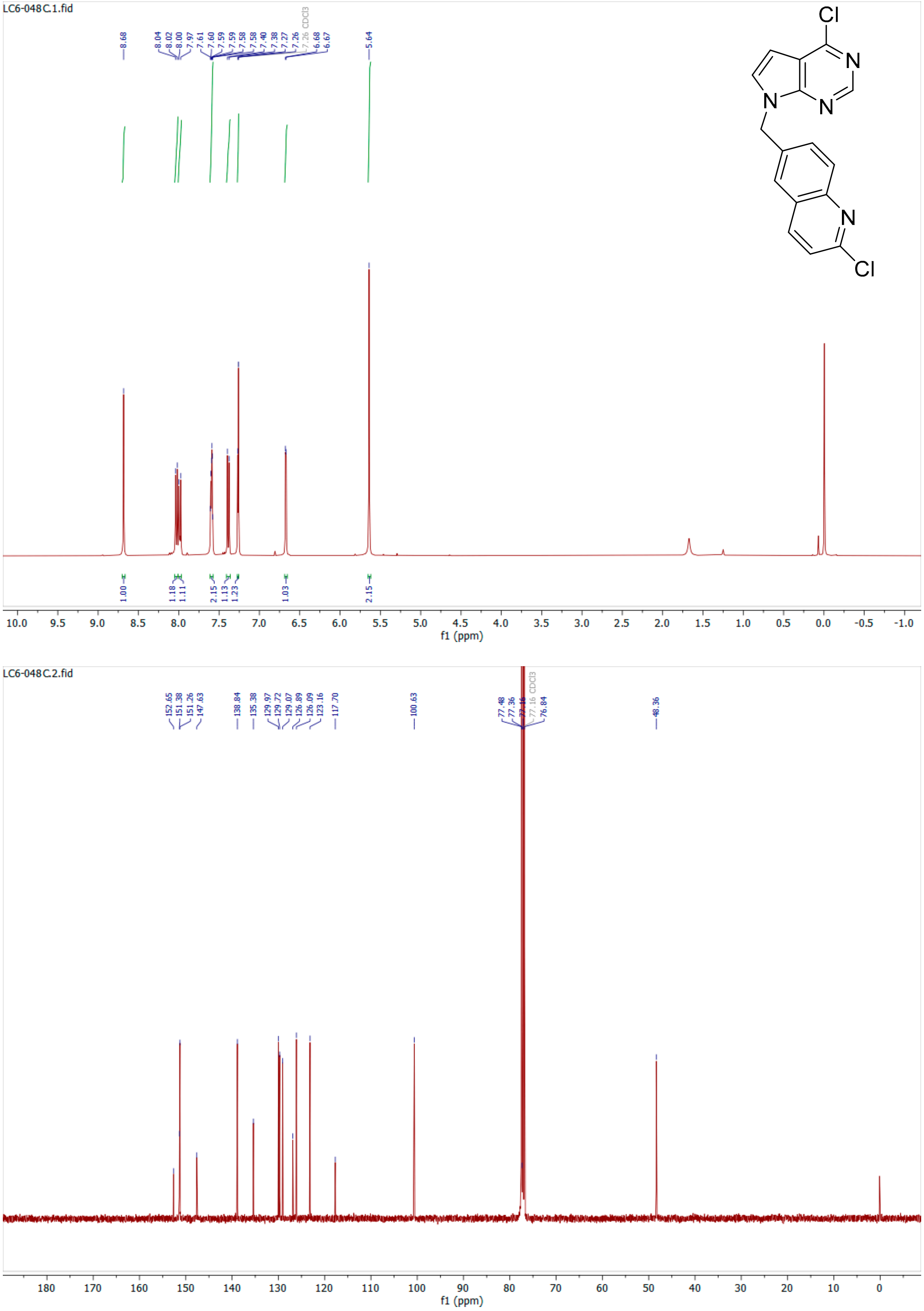

Figure S19. ^{1}H and ^{13}C spectra of chloride **34o**.

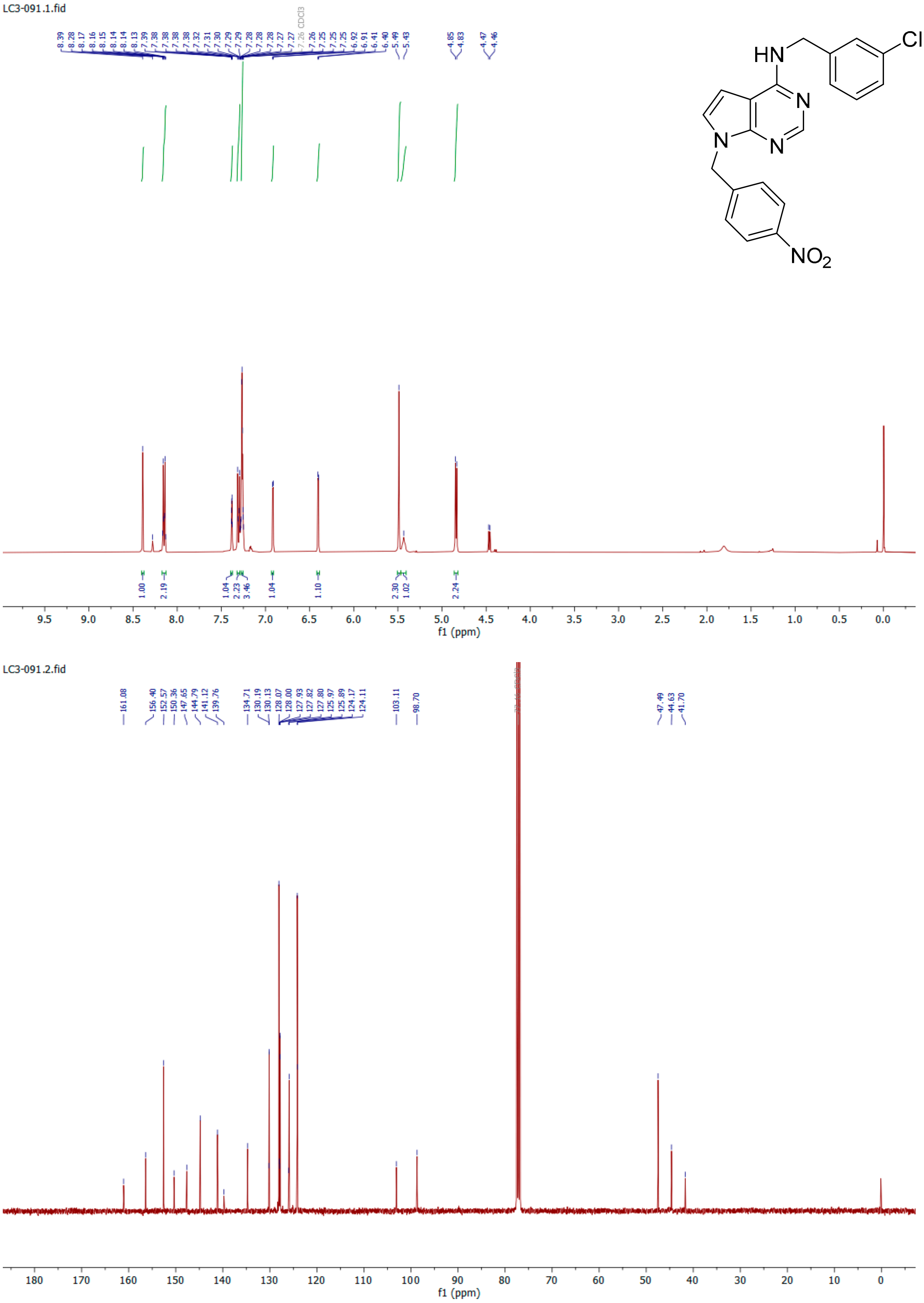

Figure S20. ^1H and ^{13}C spectra of compound **1**.

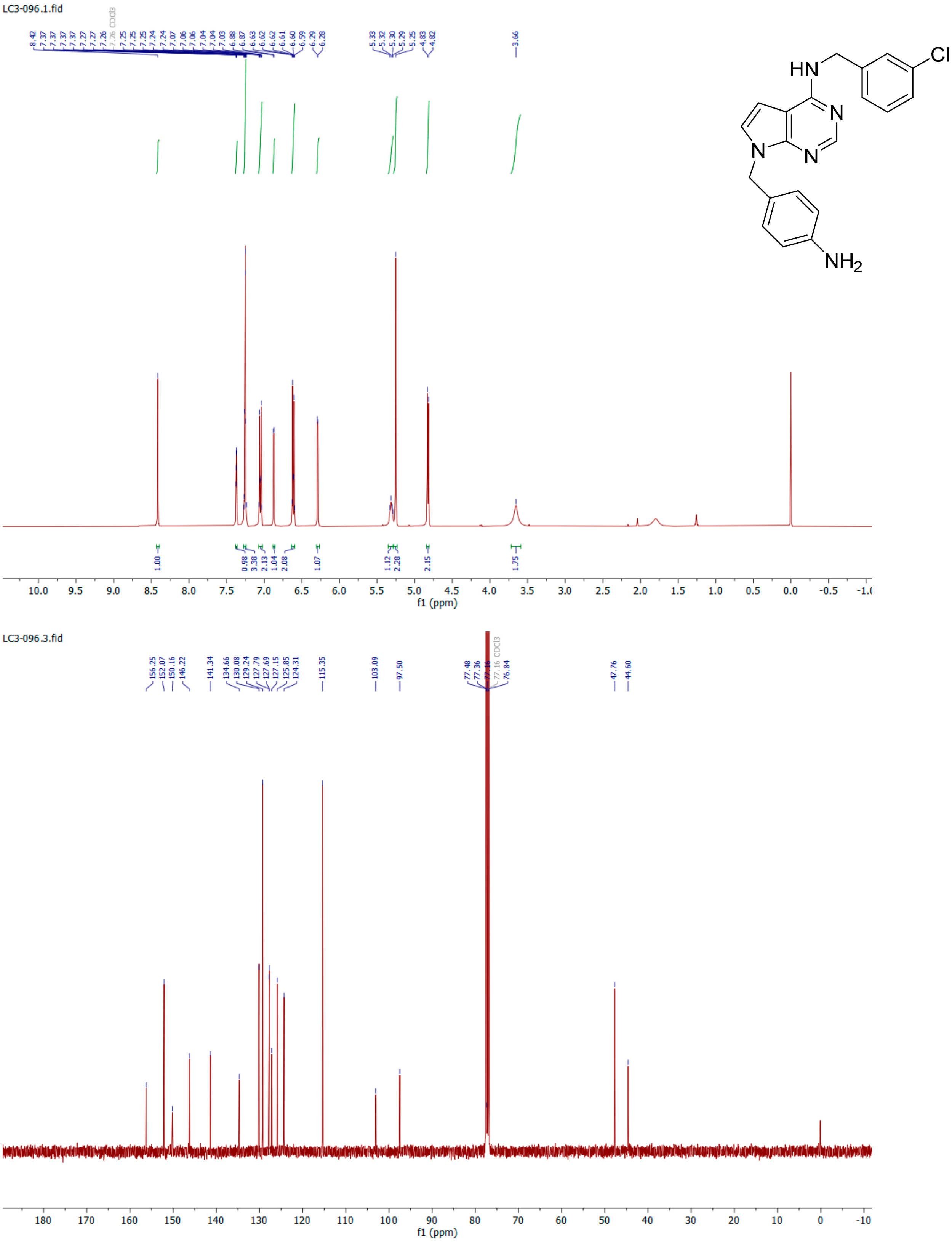

Figure S21. ^{1}H and ^{13}C spectra of compound **2**.

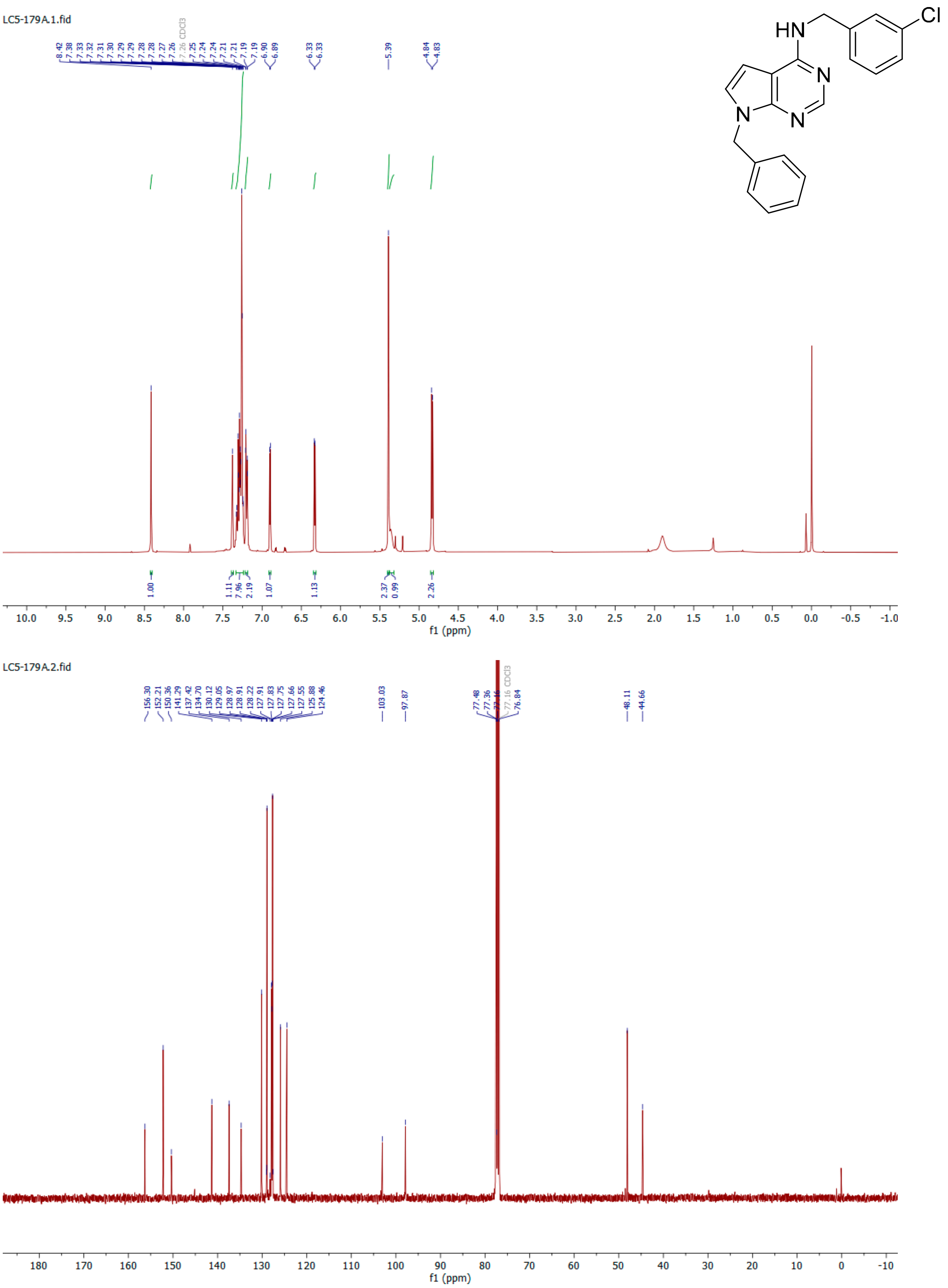

Figure S22. ^{1}H and ^{13}C spectra of compound **3**.

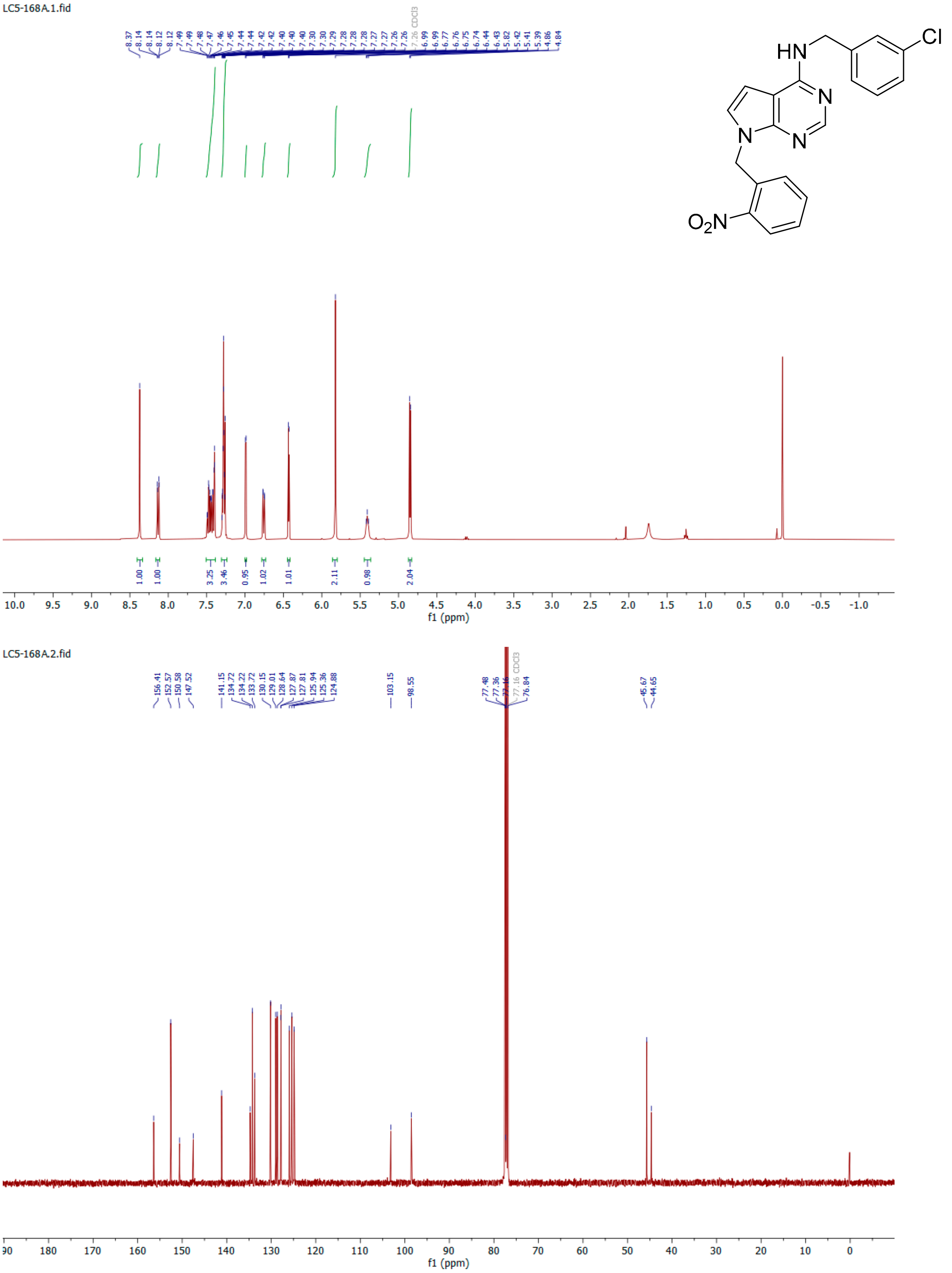

Figure S23. ^{1}H and ^{13}C spectra of compound **4**.

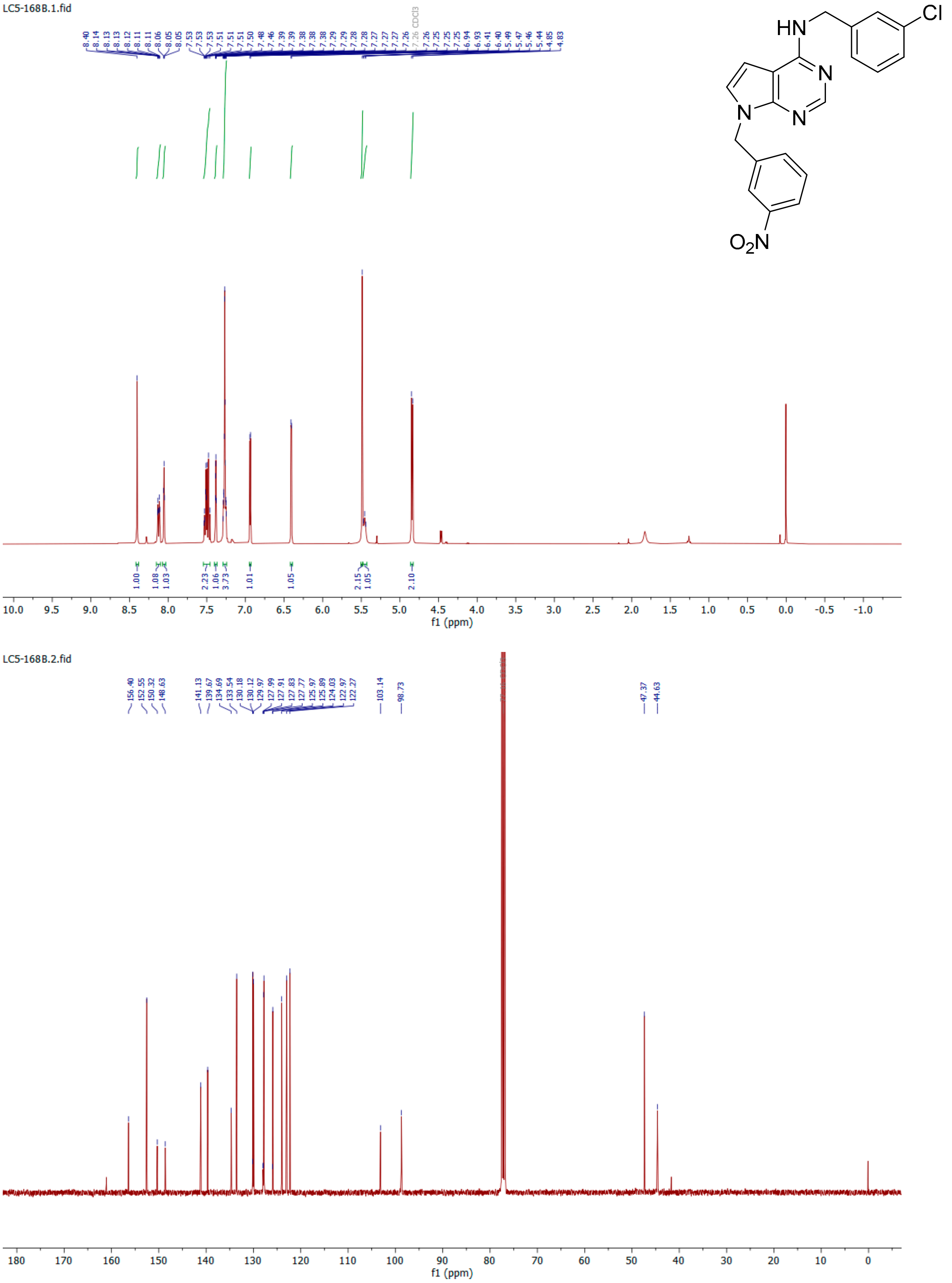

Figure S24. ^{1}H and ^{13}C spectra of compound **5**.

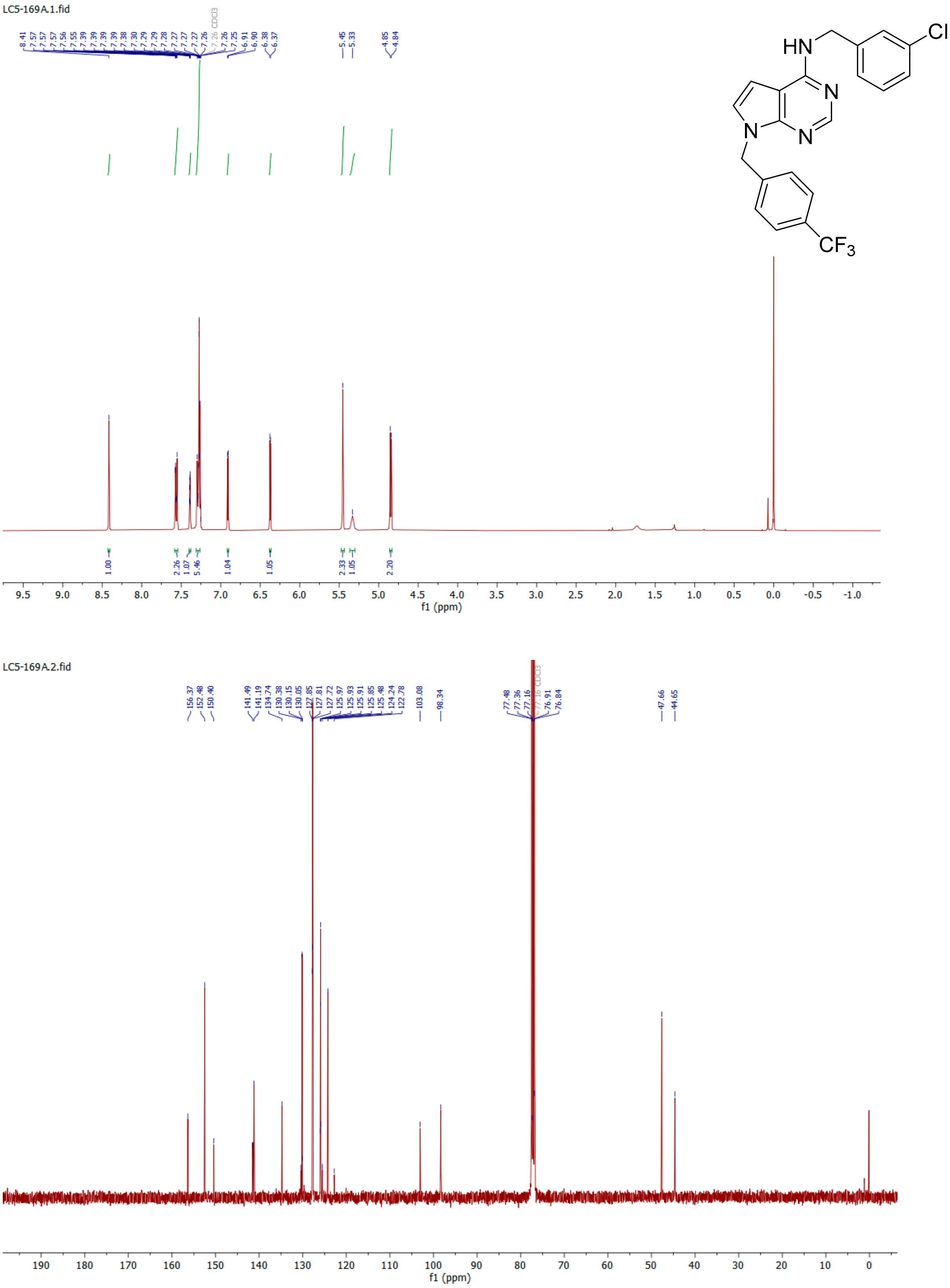

Figure S25. ^{1}H and ^{13}C spectra of compound **6**.

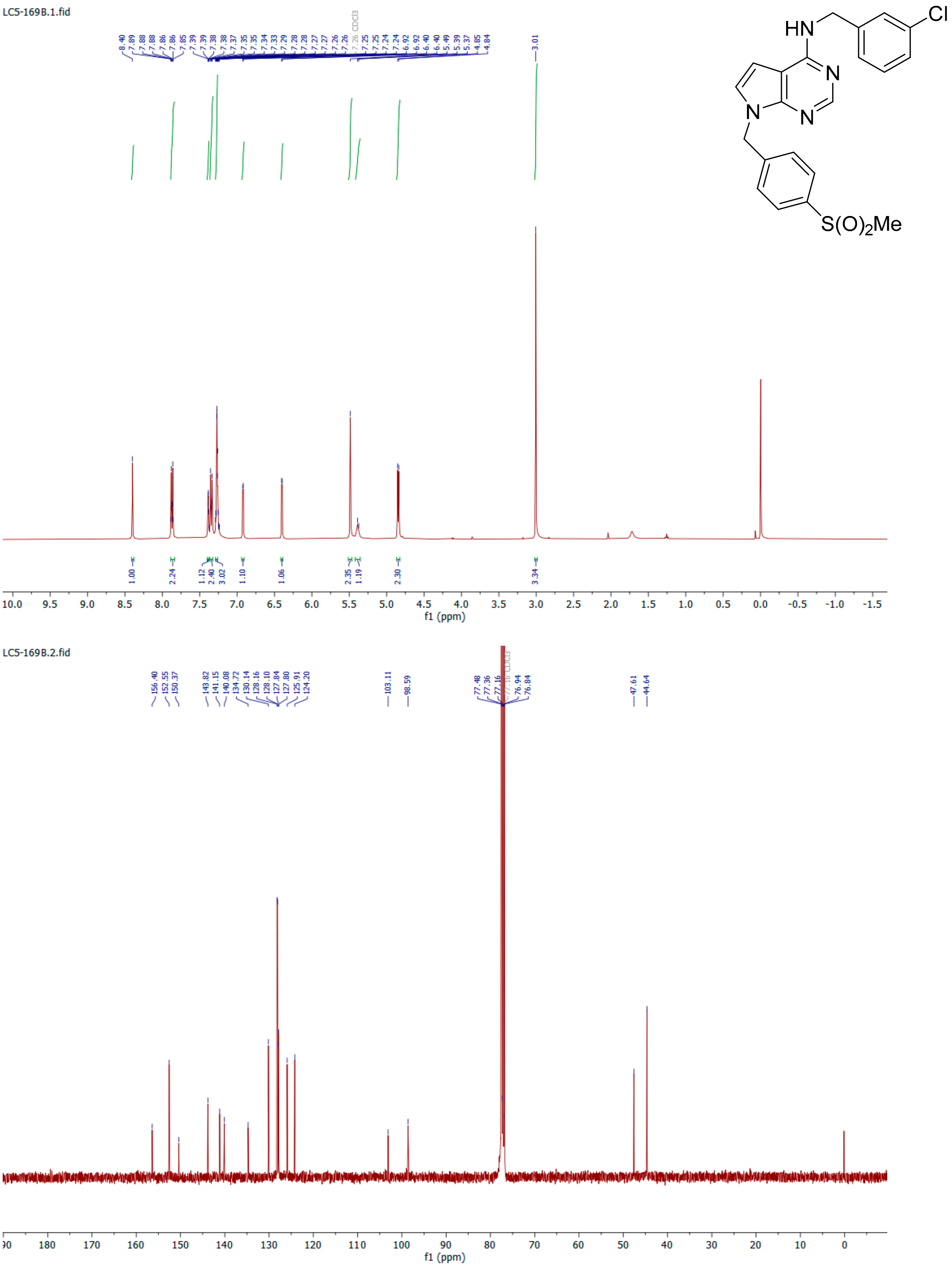

Figure S26. ^{1}H and ^{13}C spectra of compound **7**.

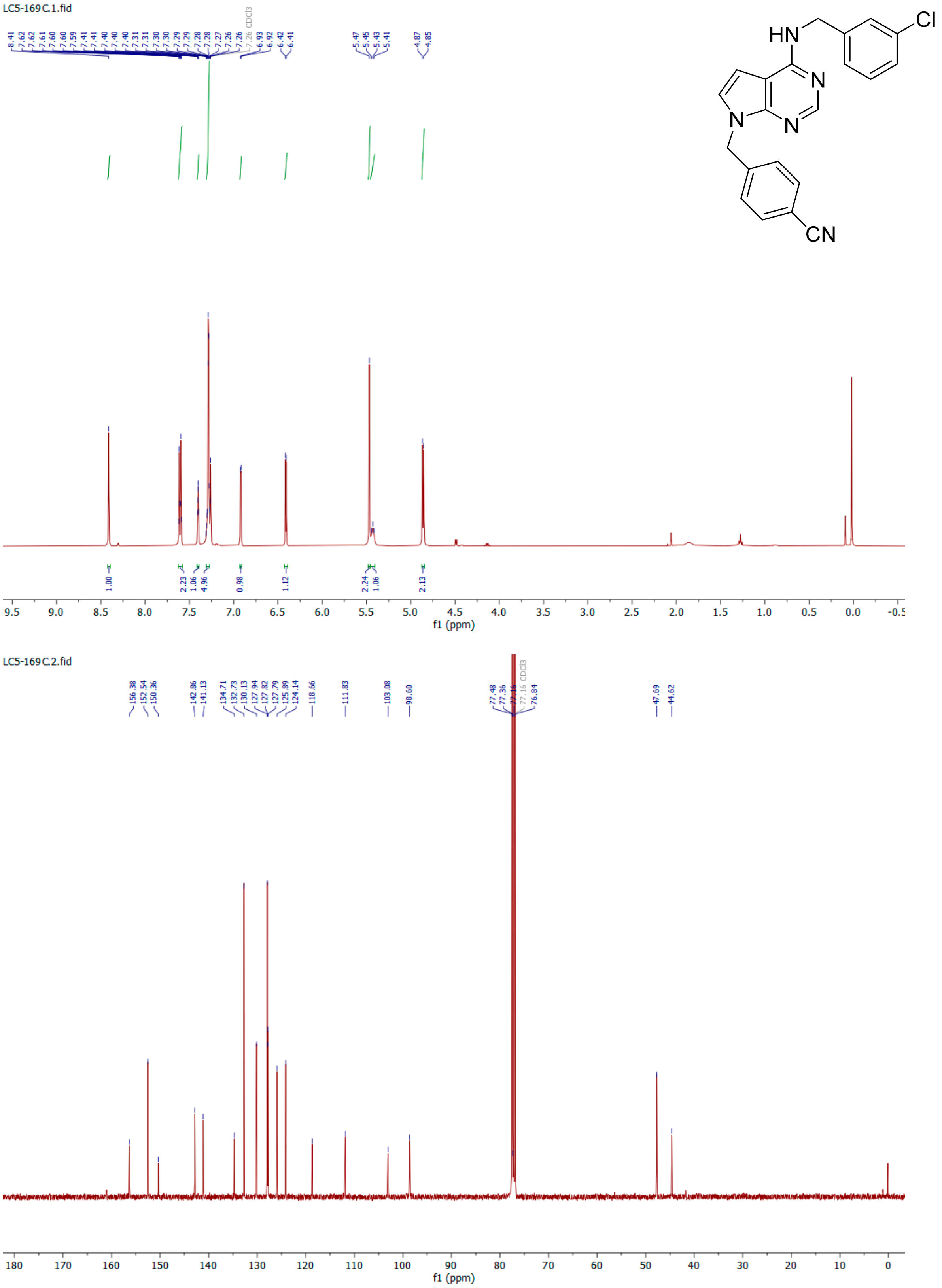

Figure S27. ^{1}H and ^{13}C spectra of compound **8**.

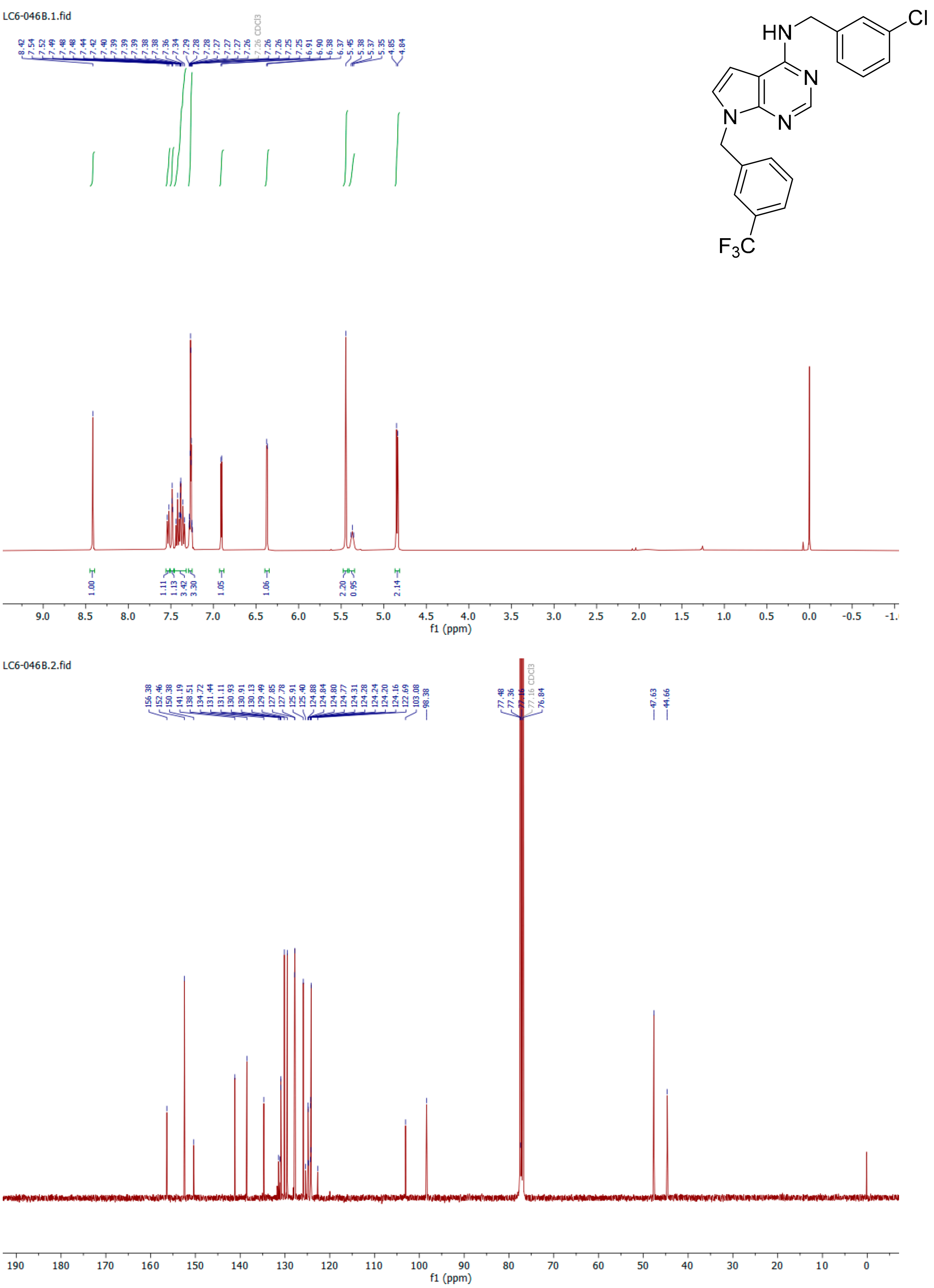

Figure S28. ^{1}H and ^{13}C spectra of compound **9**.

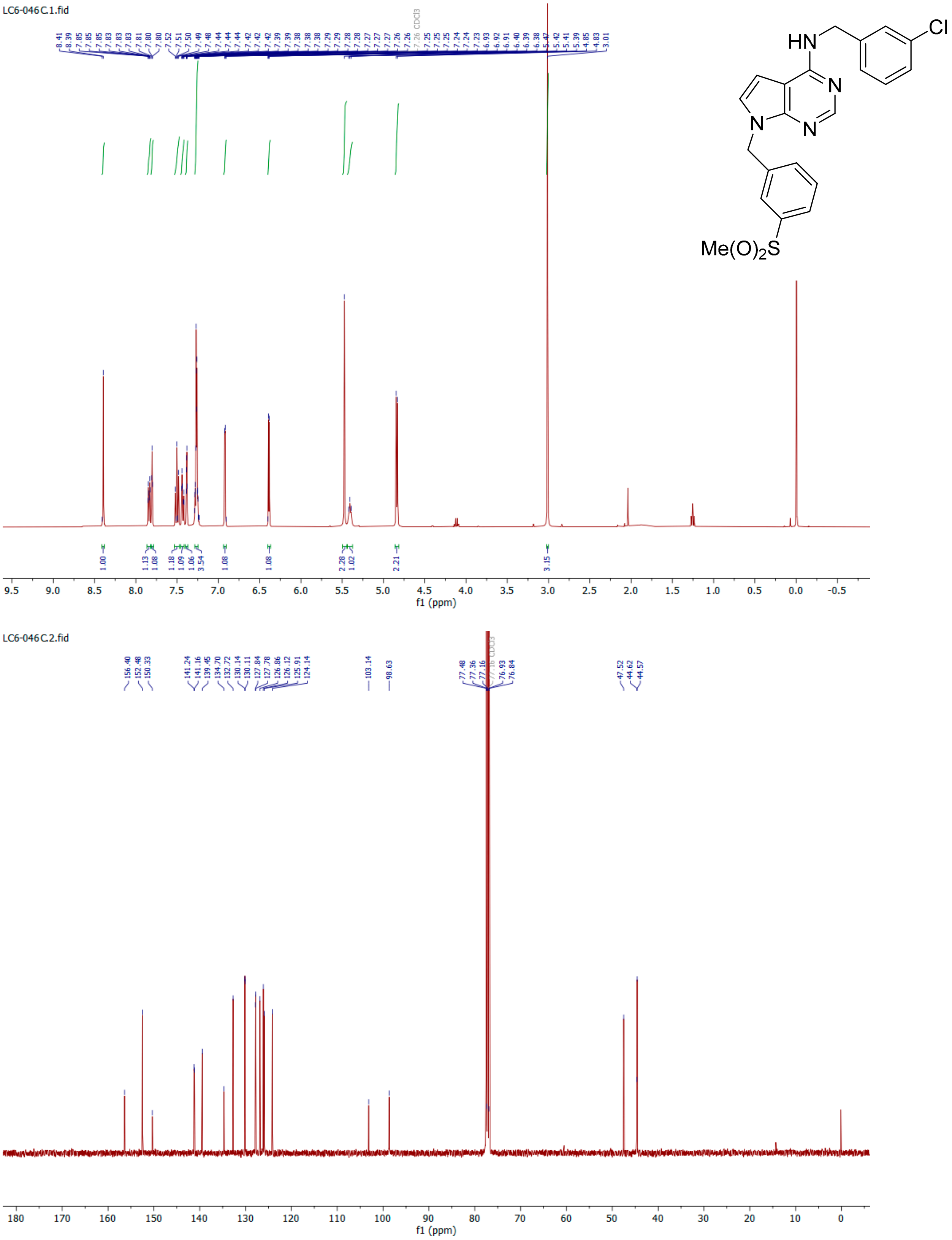

Figure S29. ^{1}H and ^{13}C spectra of compound **10**.

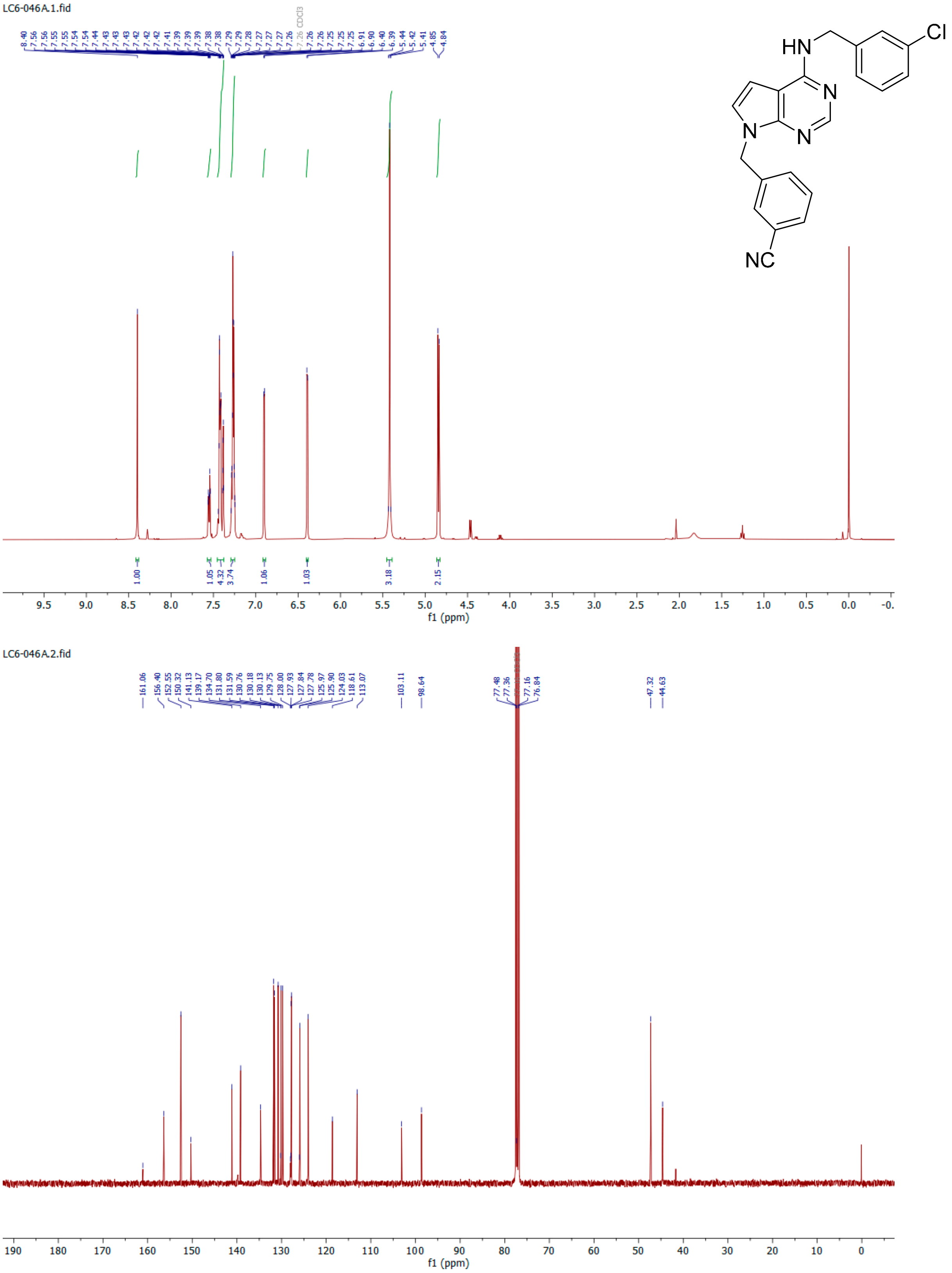

Figure S30. ^{1}H and ^{13}C spectra of compound **11**.

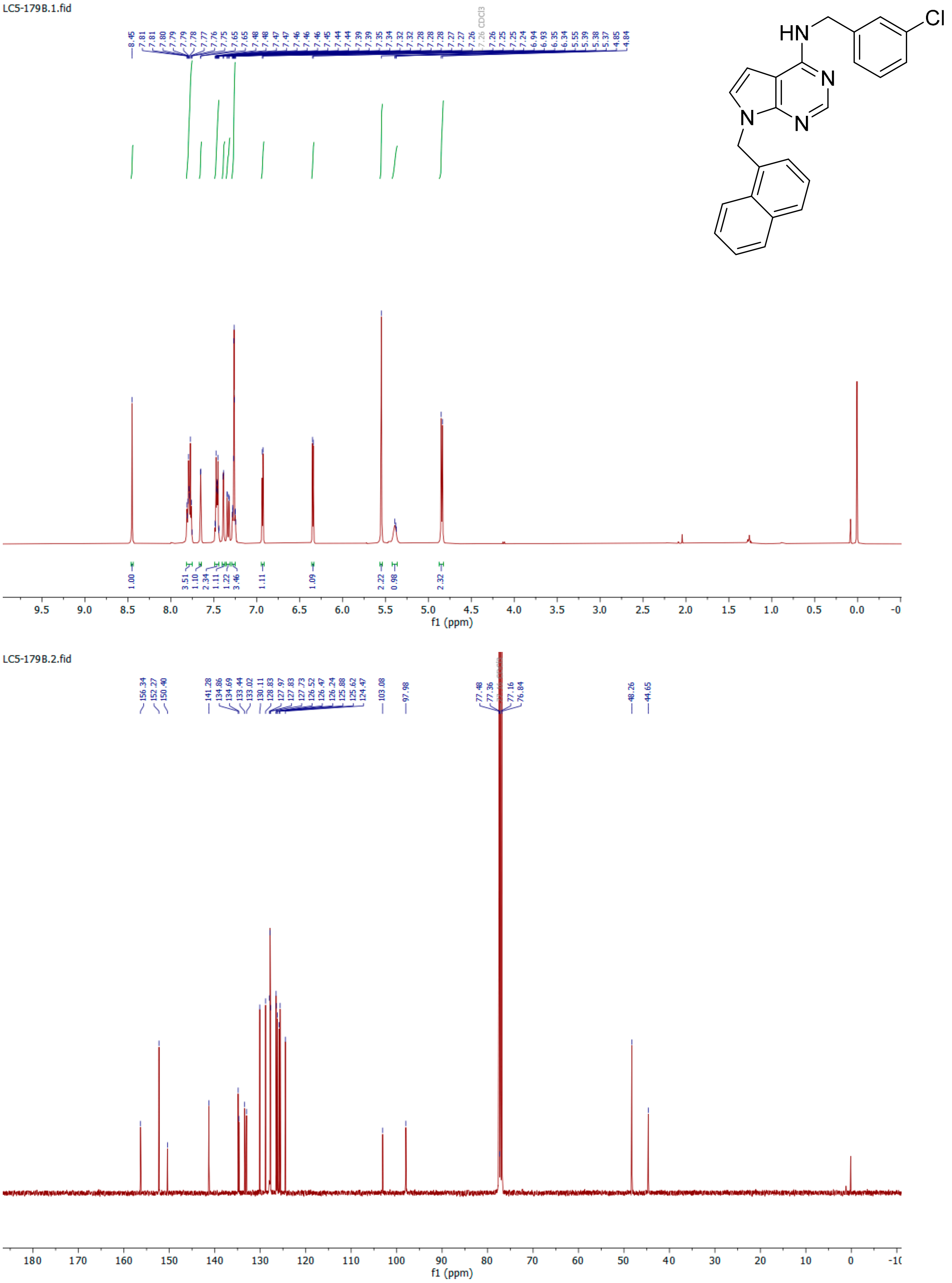

Figure S31. ^{1}H and ^{13}C spectra of compound **12**.

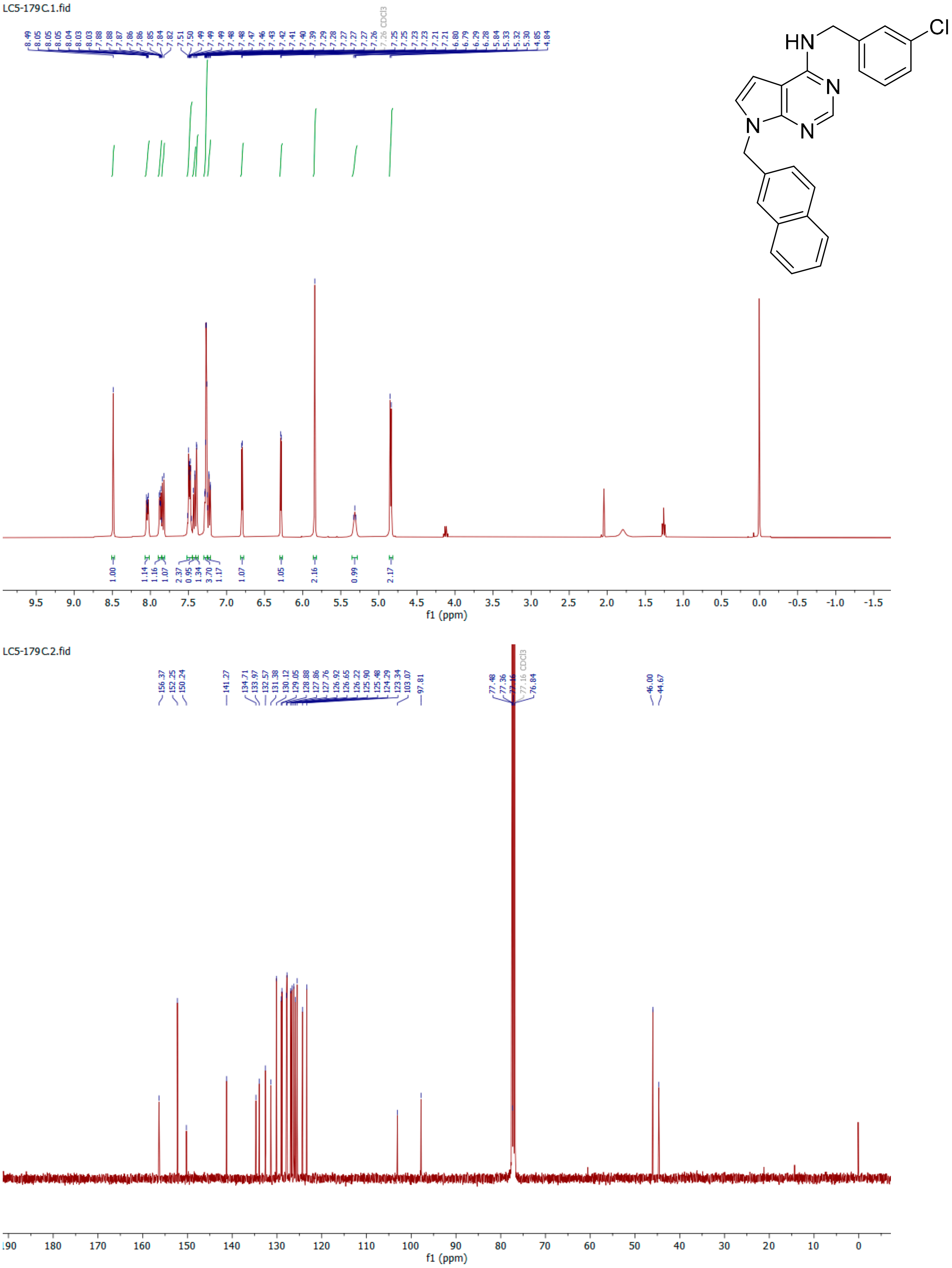

Figure S32. ^{1}H and ^{13}C spectra of compound **13**.

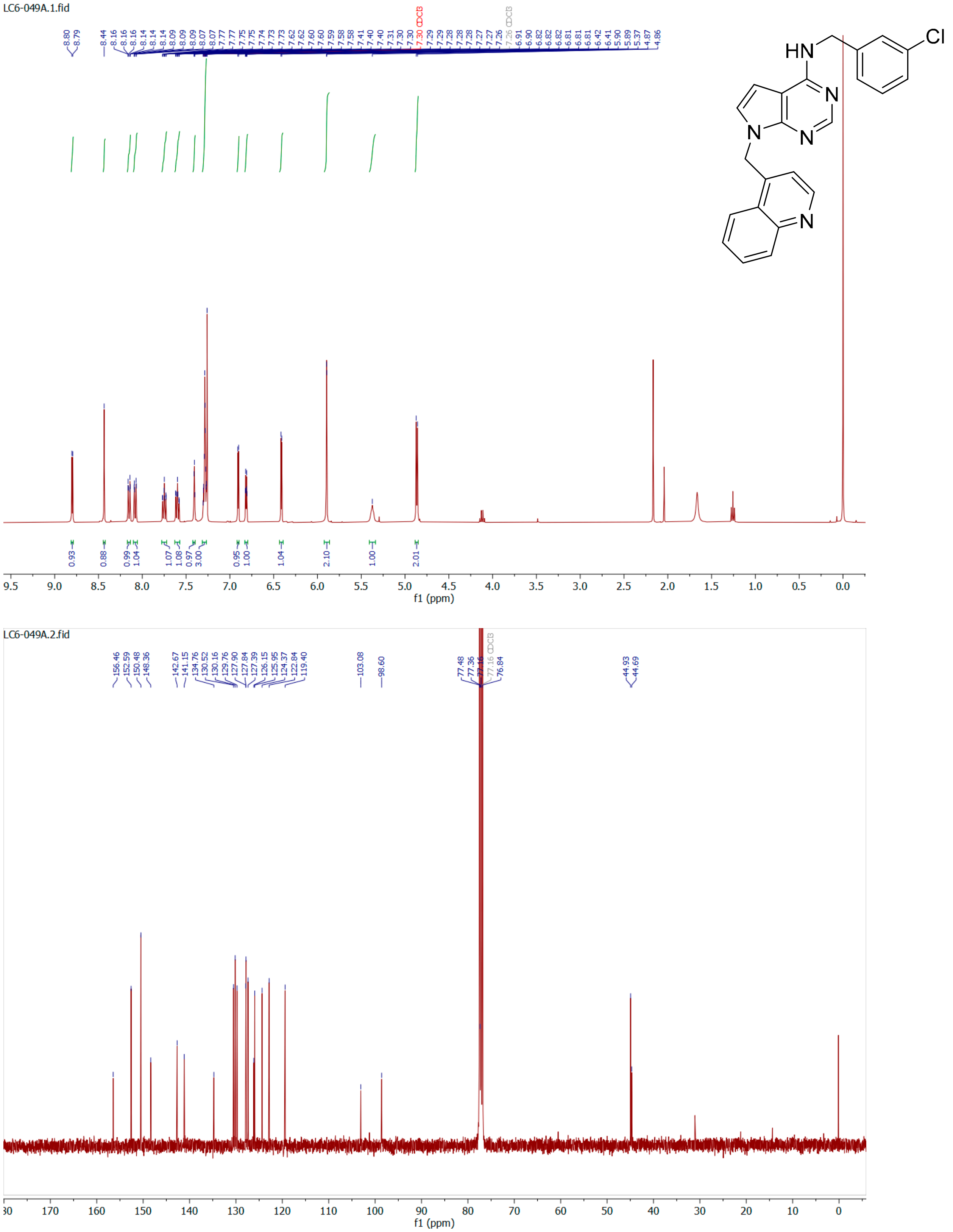

Figure S33. ^{1}H and ^{13}C spectra of compound **14**.

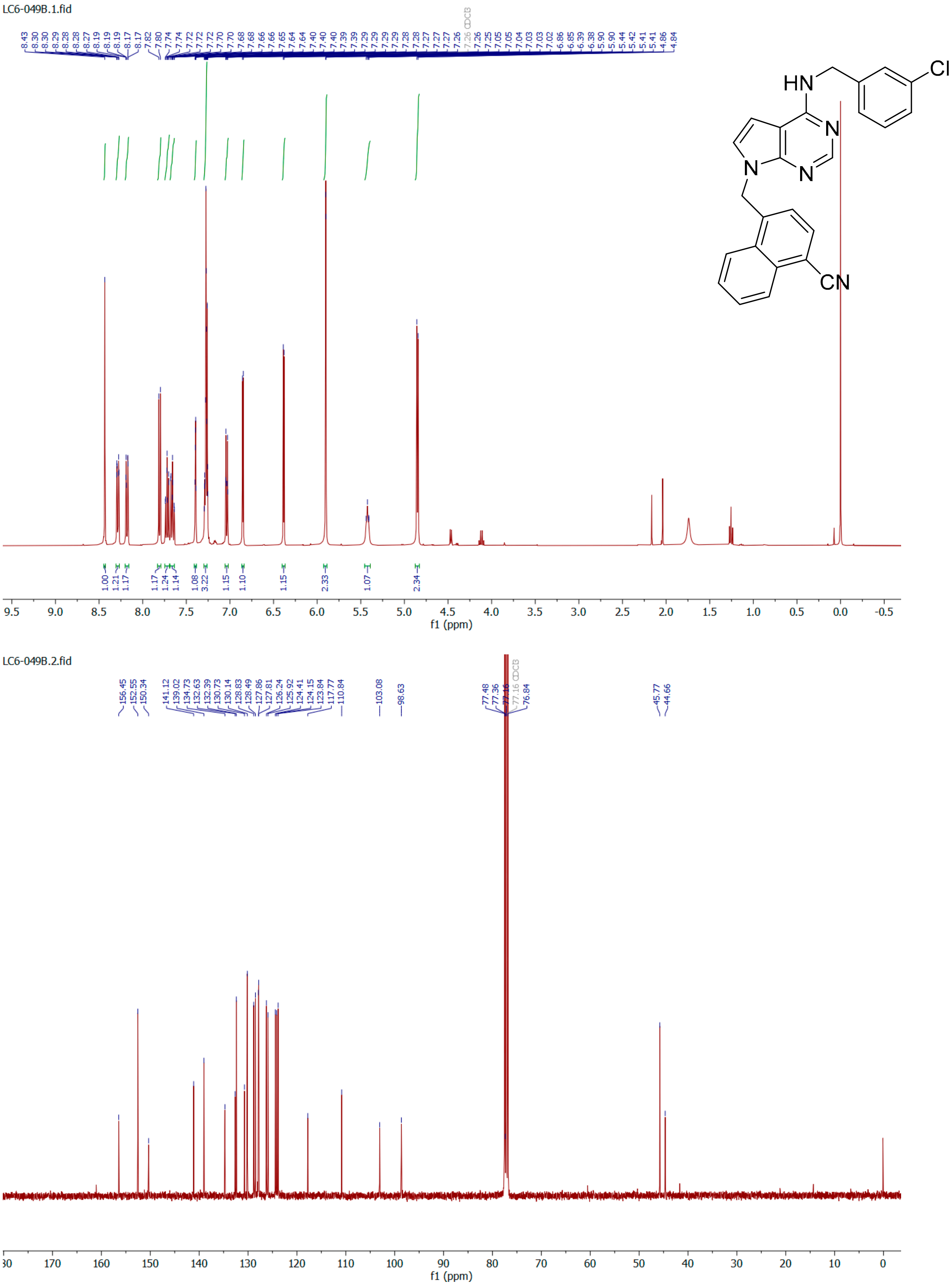

Figure S34. ^{1}H and ^{13}C spectra of compound **15**.

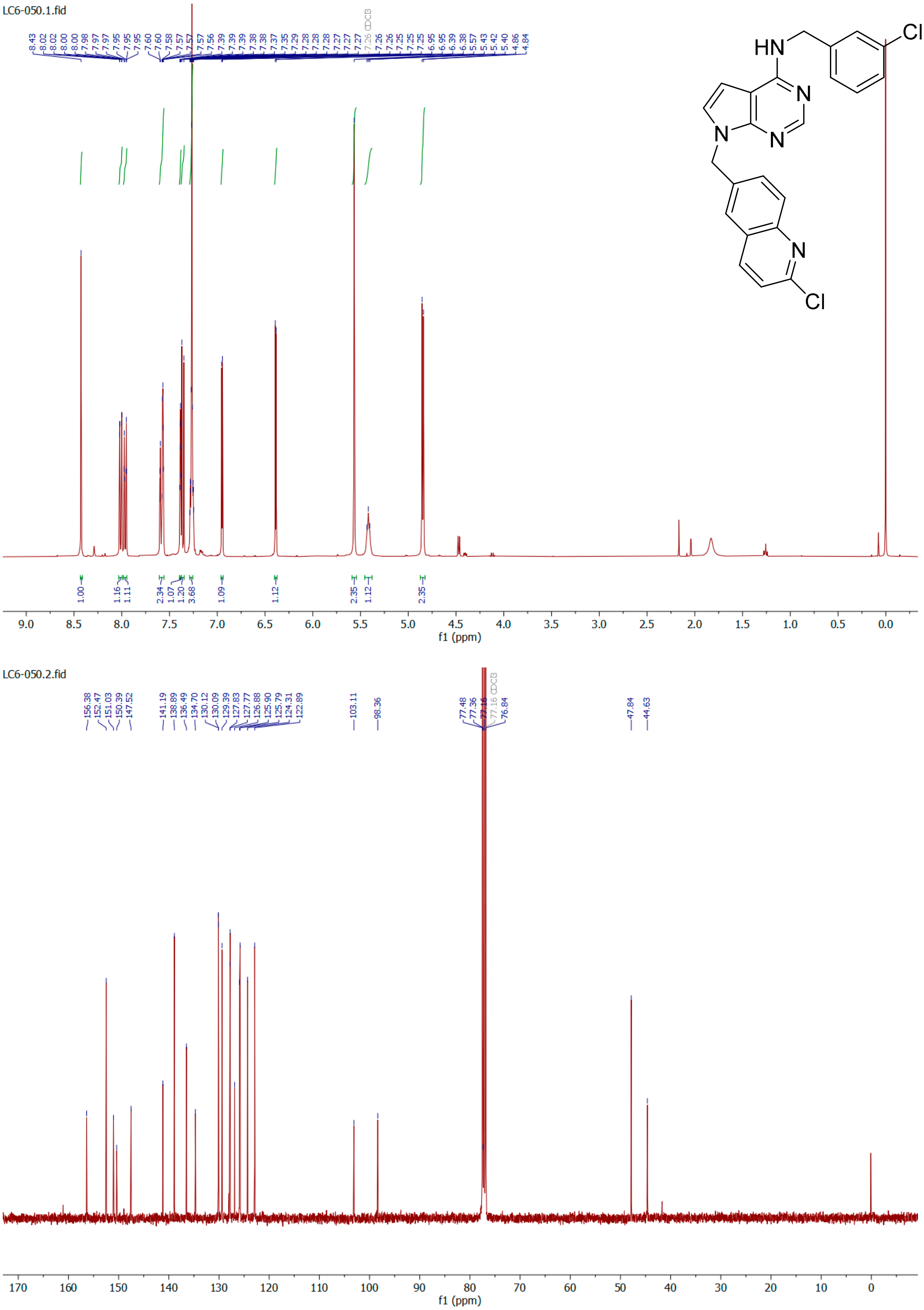

Figure S35. ^{1}H and ^{13}C spectra of compound **16**.

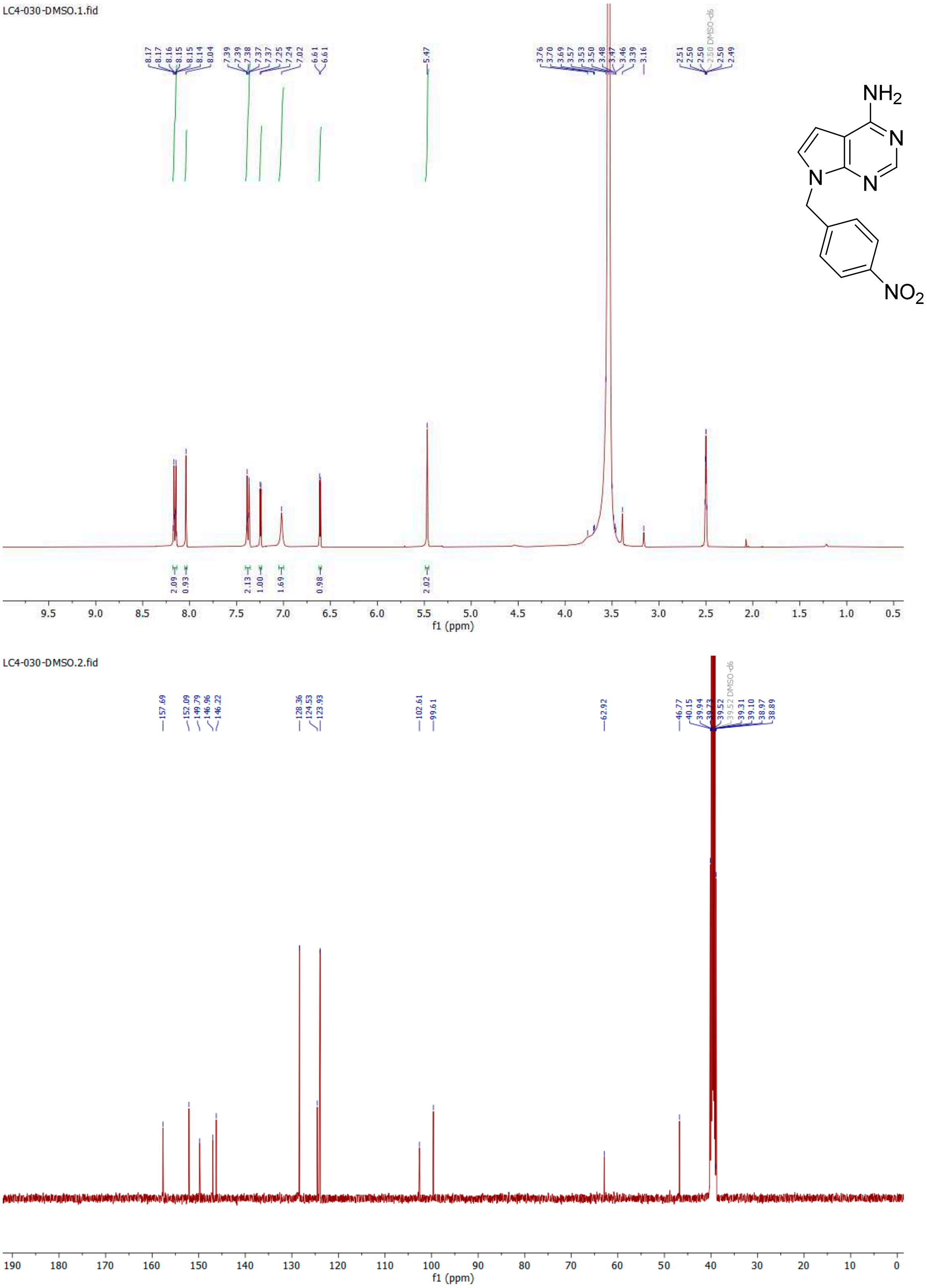

Figure S36. ^{1}H and ^{13}C spectra of compound **17**.

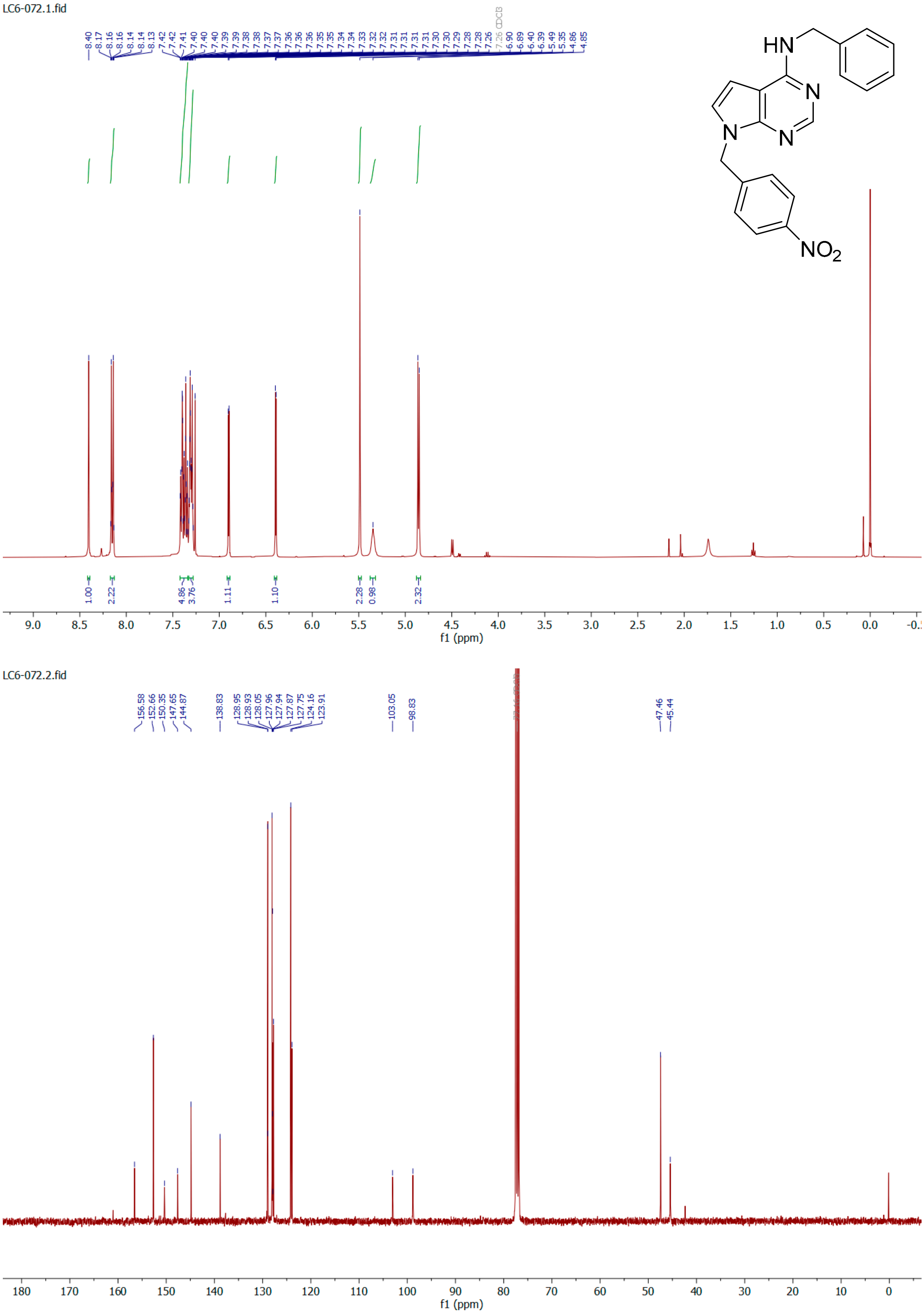

Figure S37. ^{1}H and ^{13}C spectra of compound **18**.

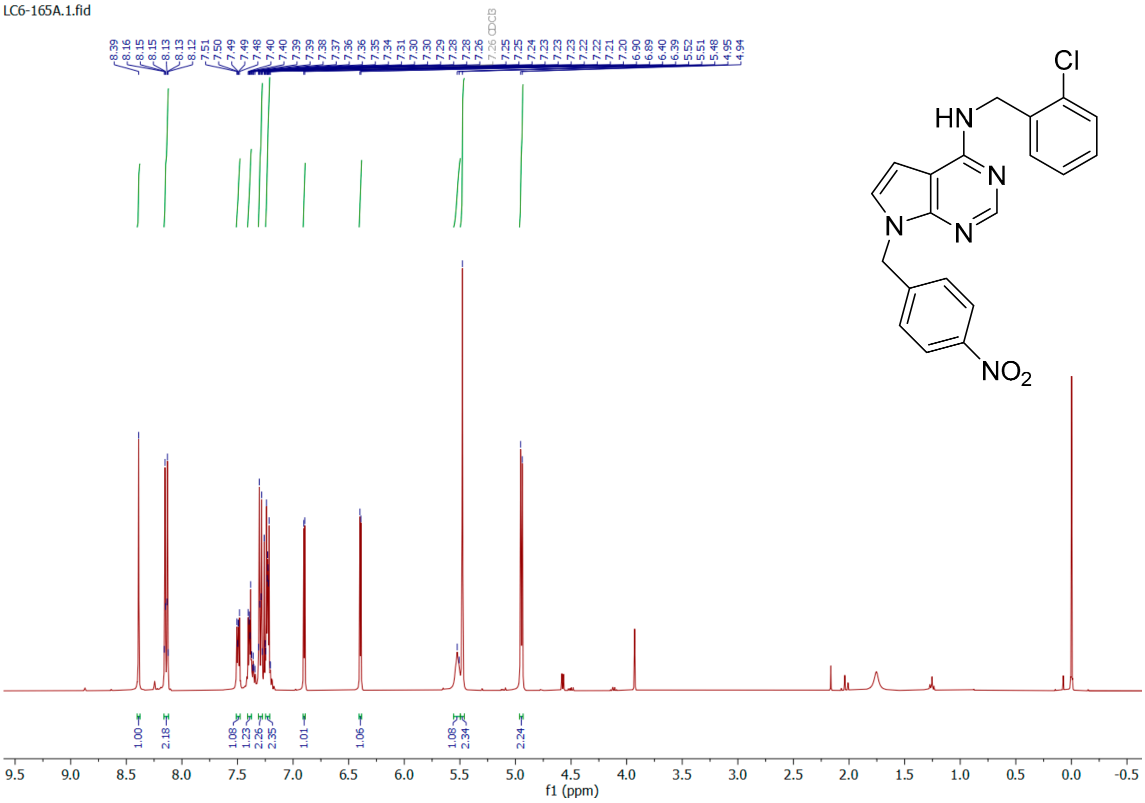

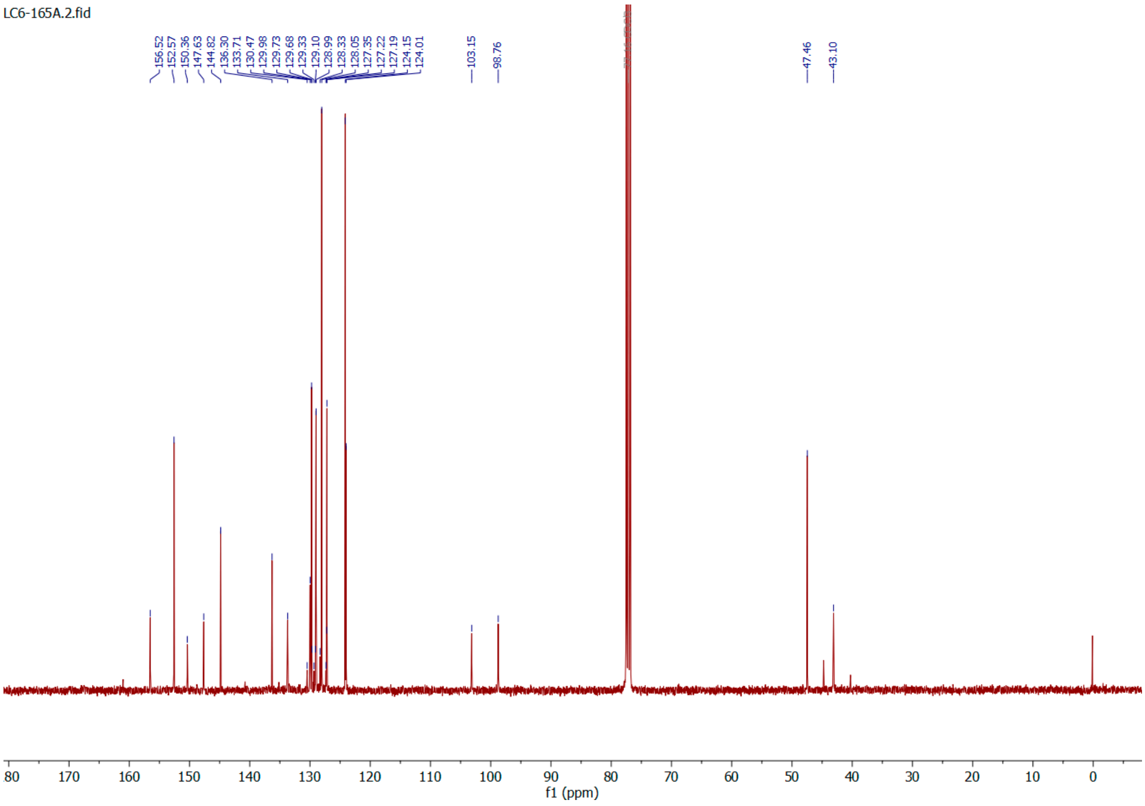

Figure S38. ^{1}H and ^{13}C spectra of compound **19**.

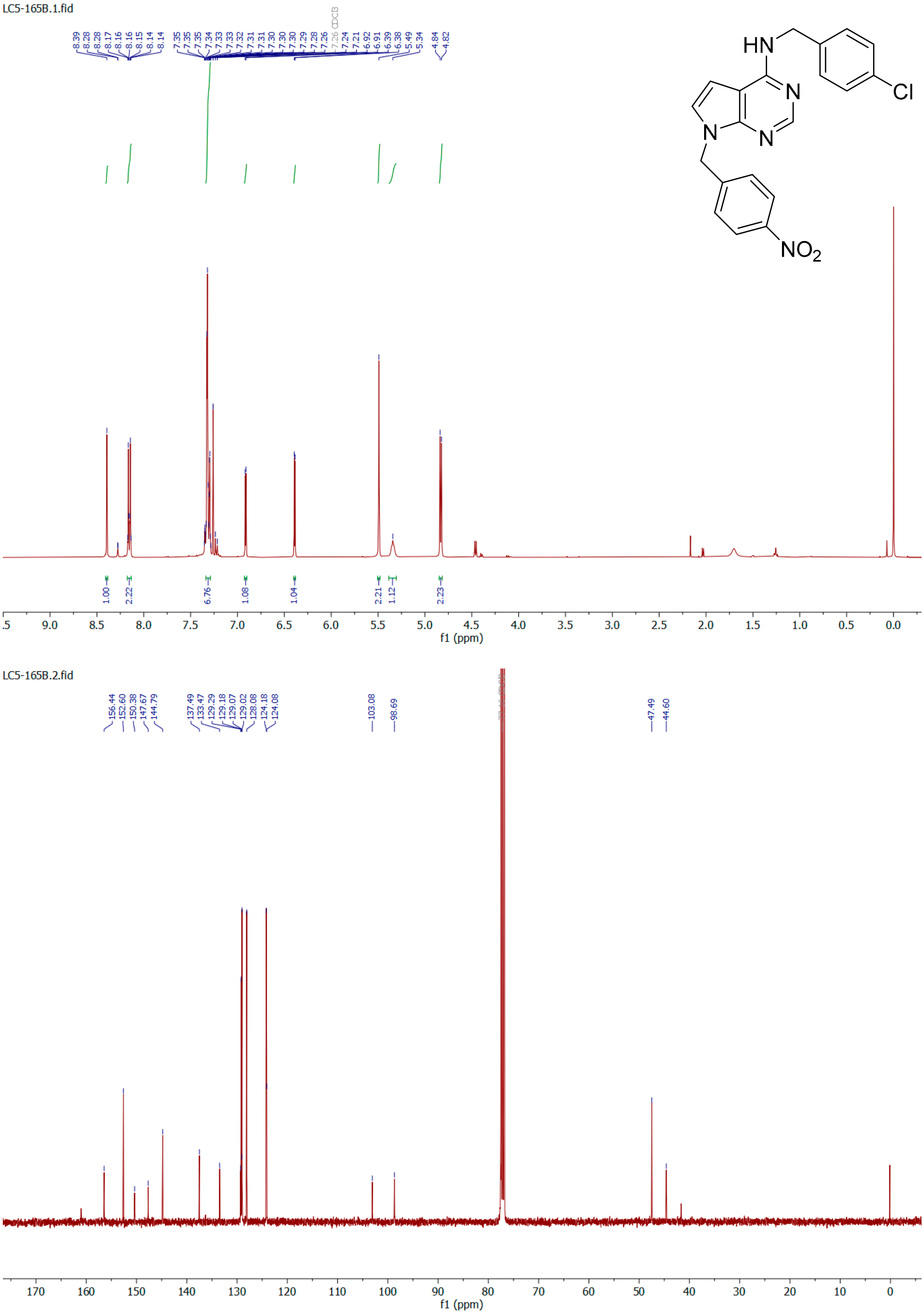

Figure S39. ^{1}H and ^{13}C spectra of compound **20**.

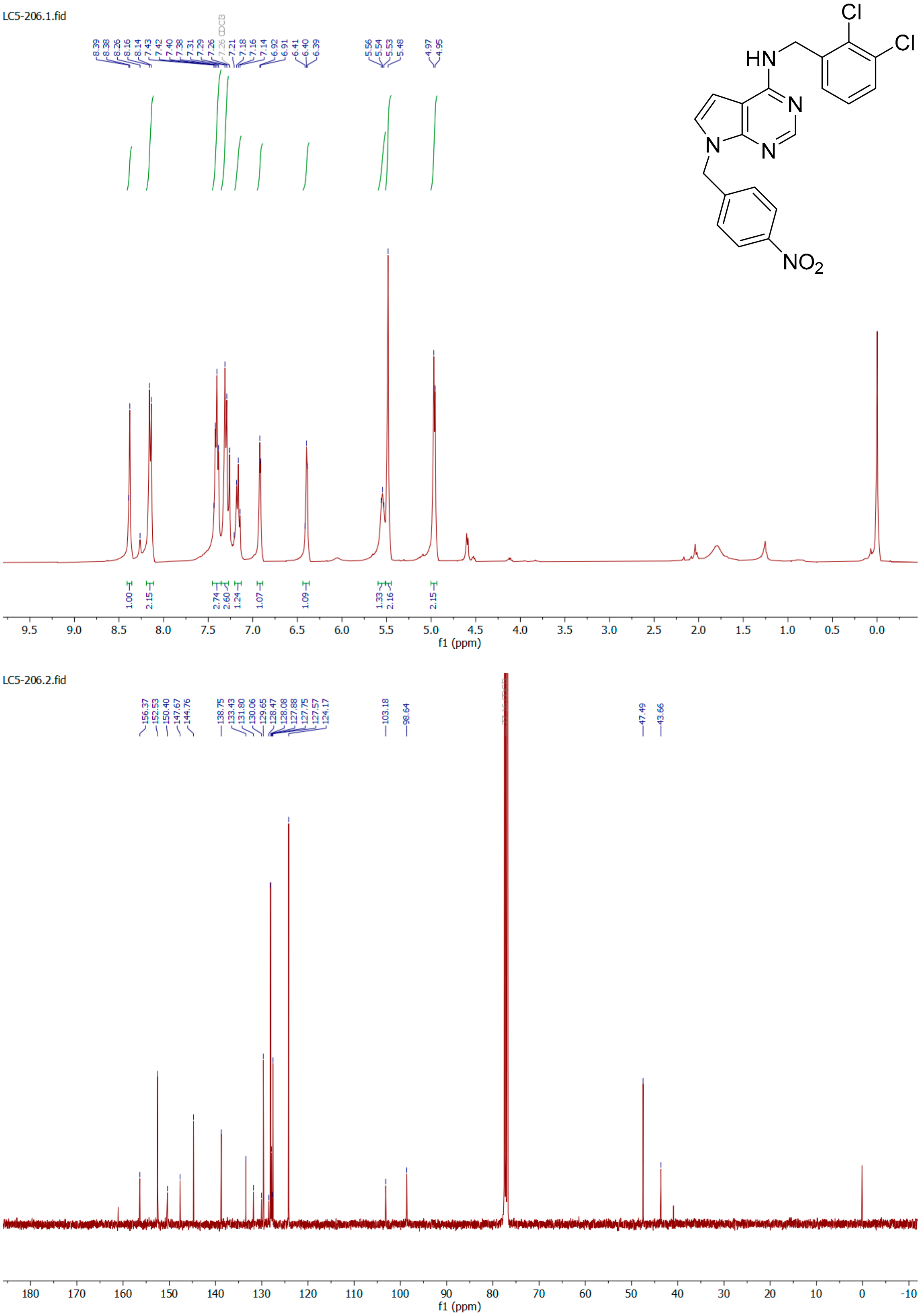

Figure S40. ^{1}H and ^{13}C spectra of compound **21**.

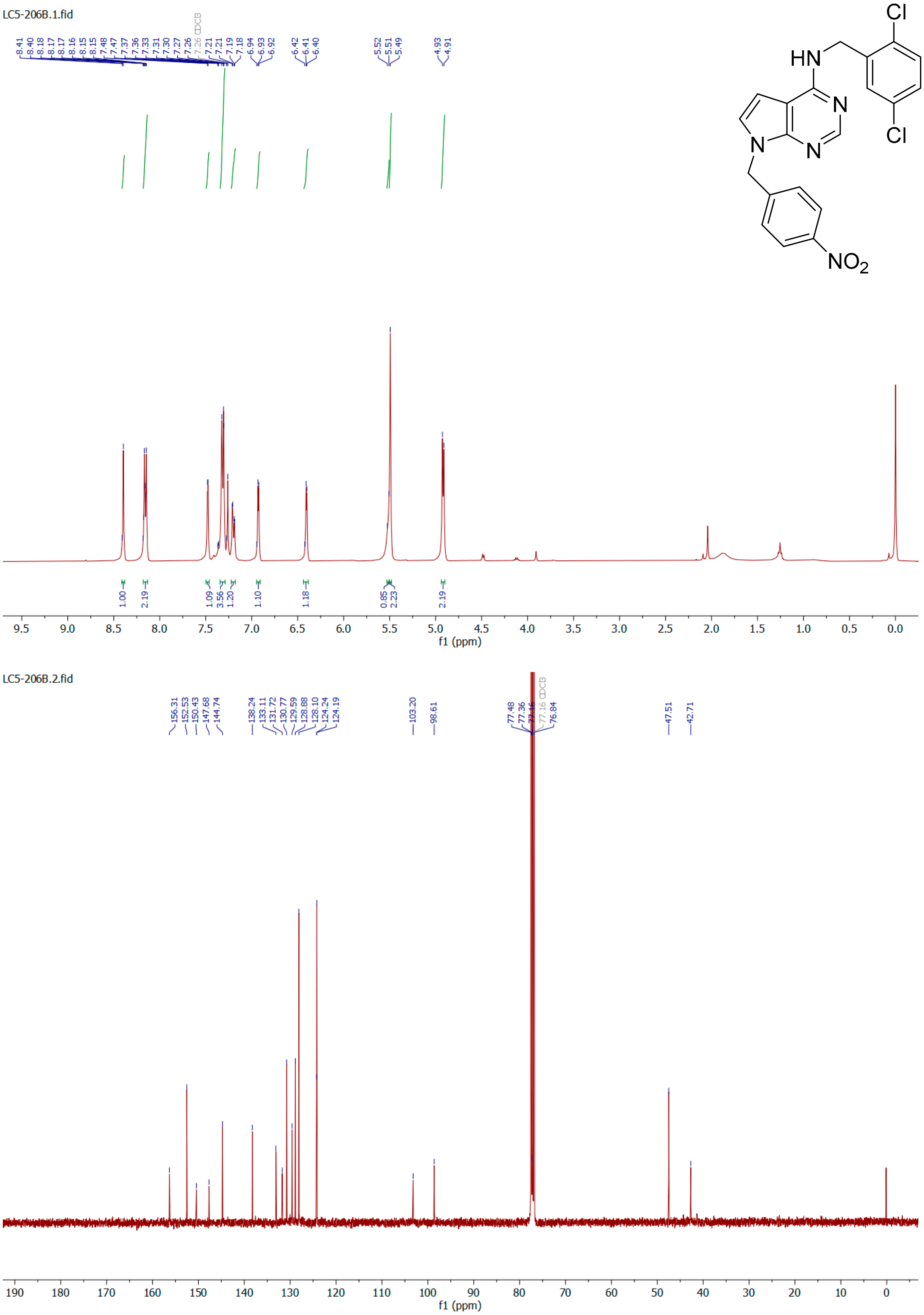

Figure S41. ^{1}H and ^{13}C spectra of compound **22**.

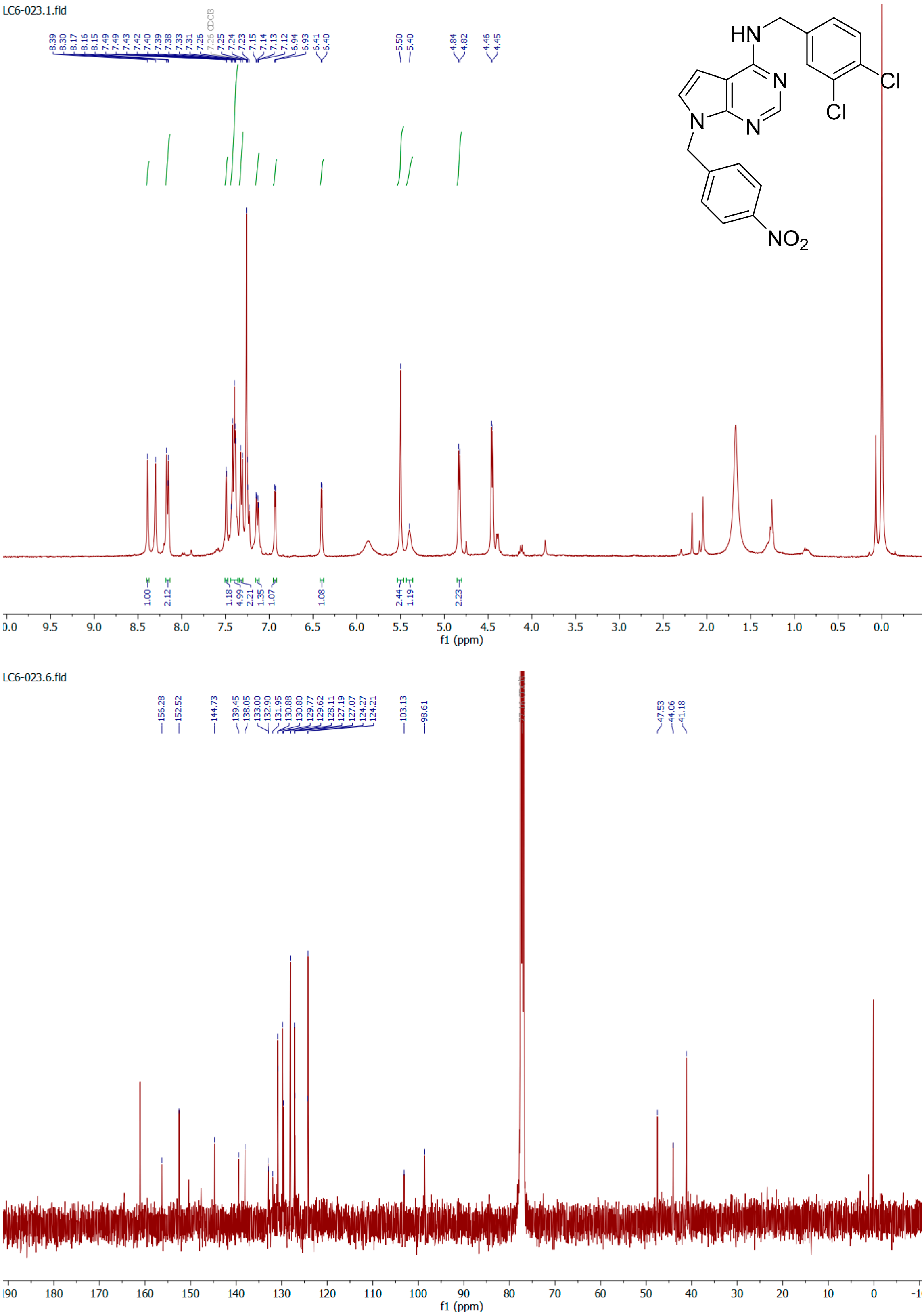

Figure S42. ^{1}H and ^{13}C spectra of compound **23**.

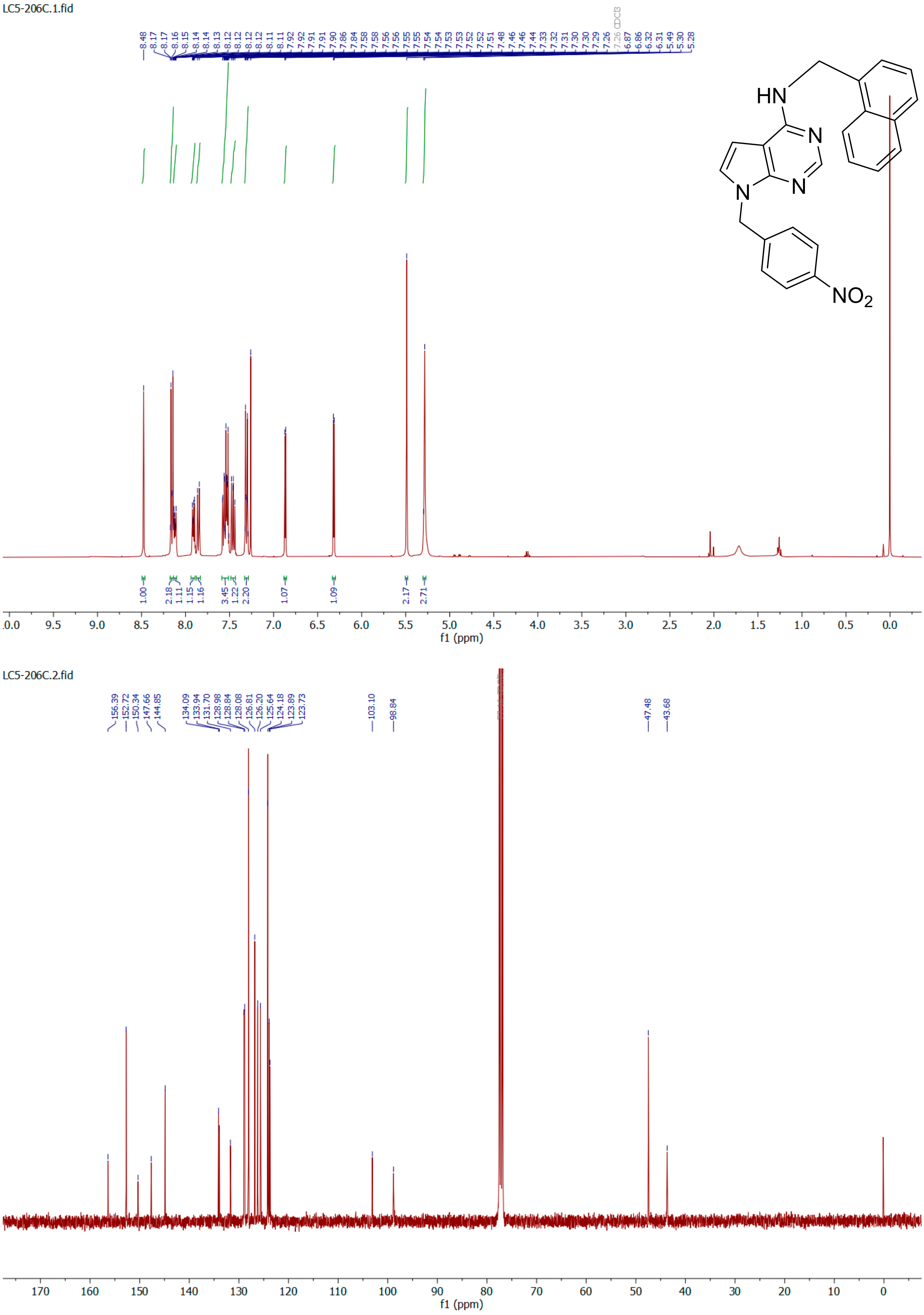

Figure S43. ^{1}H and ^{13}C spectra of compound **24**.

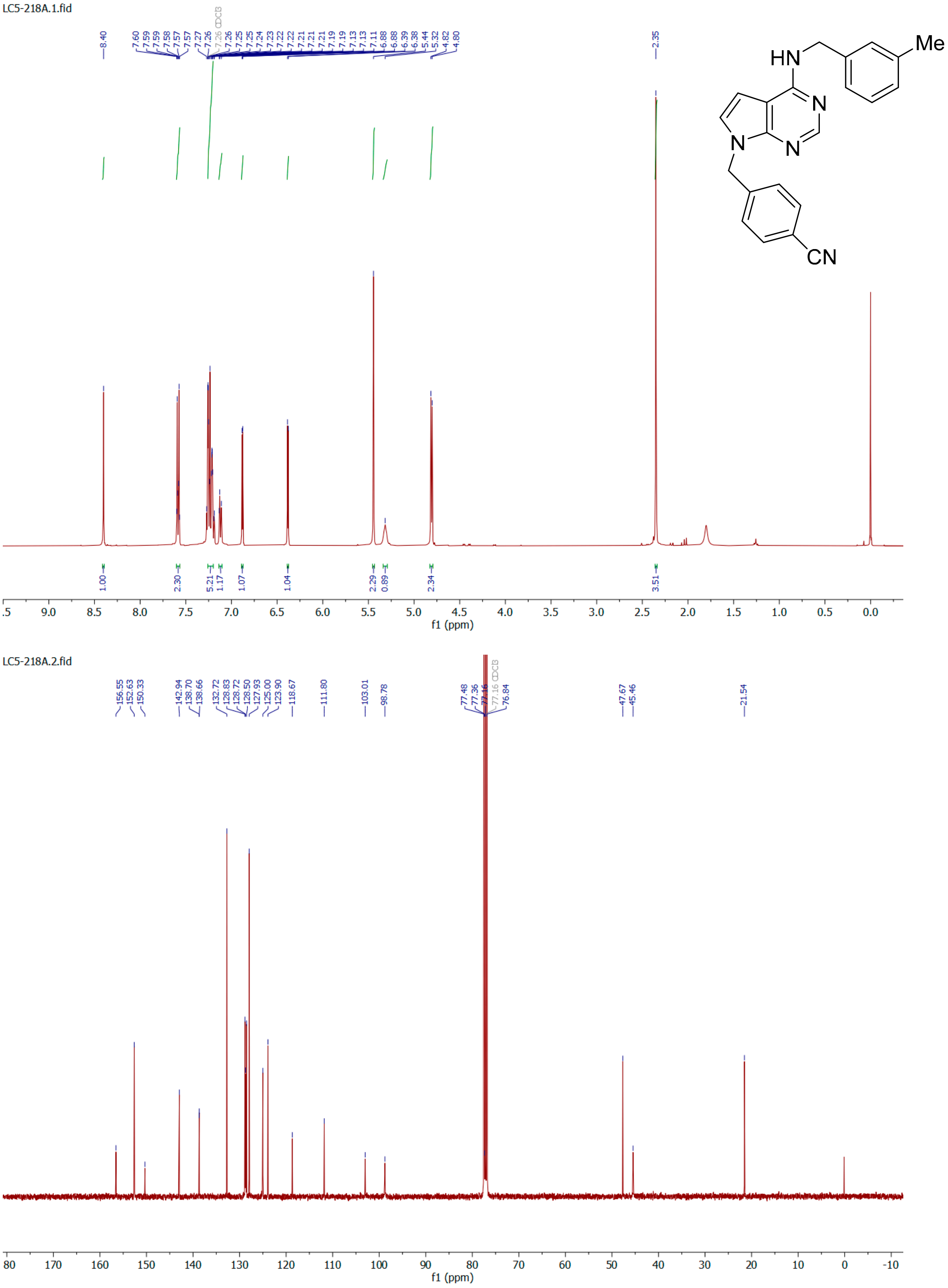

Figure S44. ^{1}H and ^{13}C spectra of compound **25**.

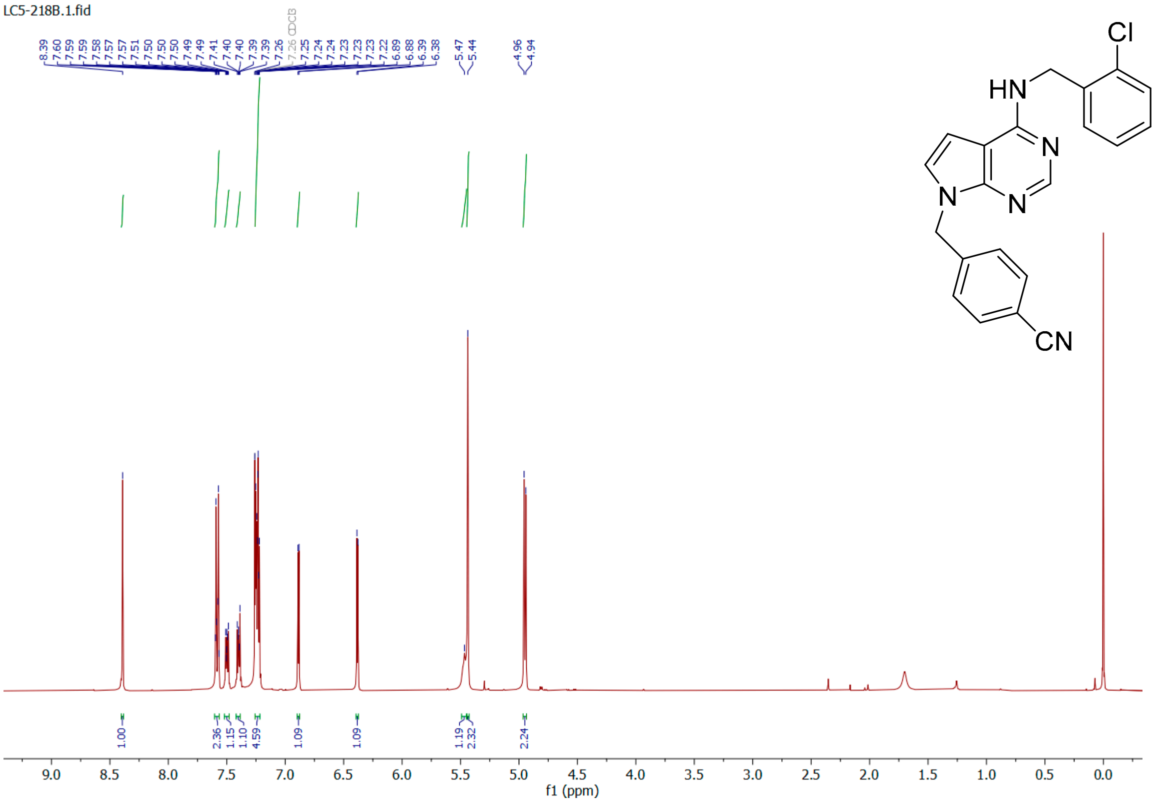

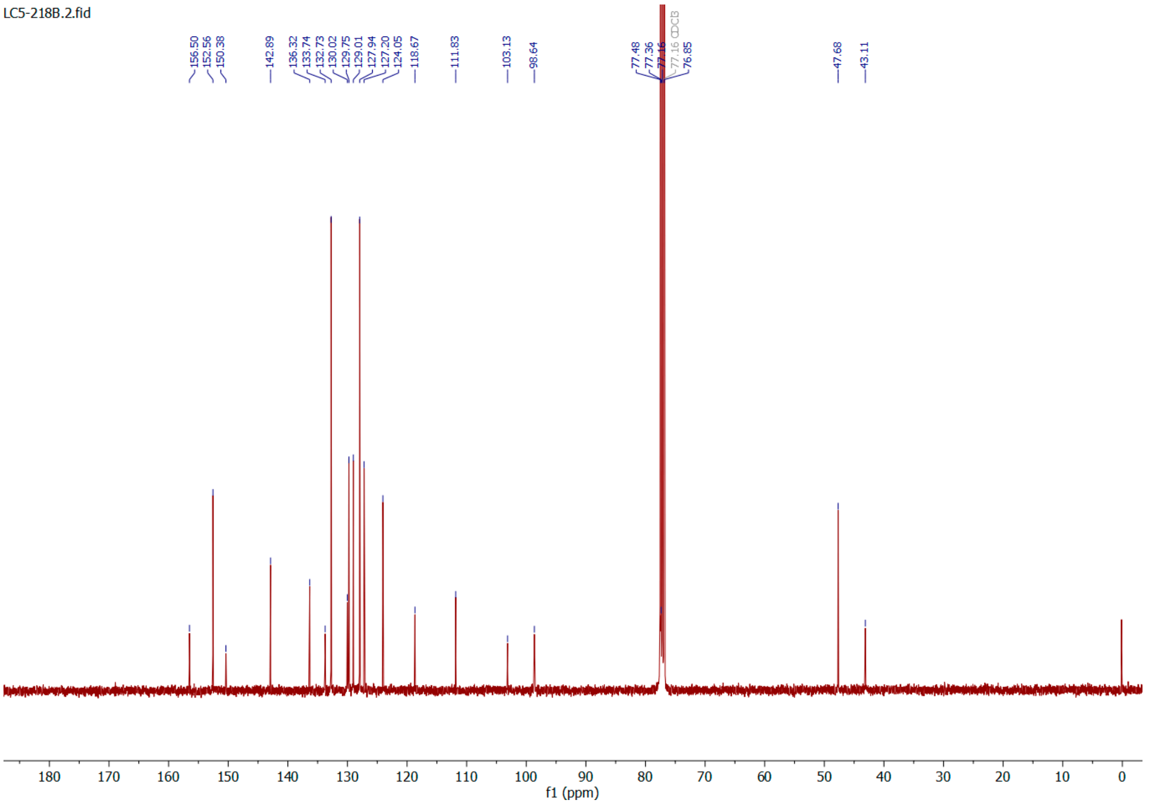

Figure S45. ^{1}H and ^{13}C spectra of compound **26**.

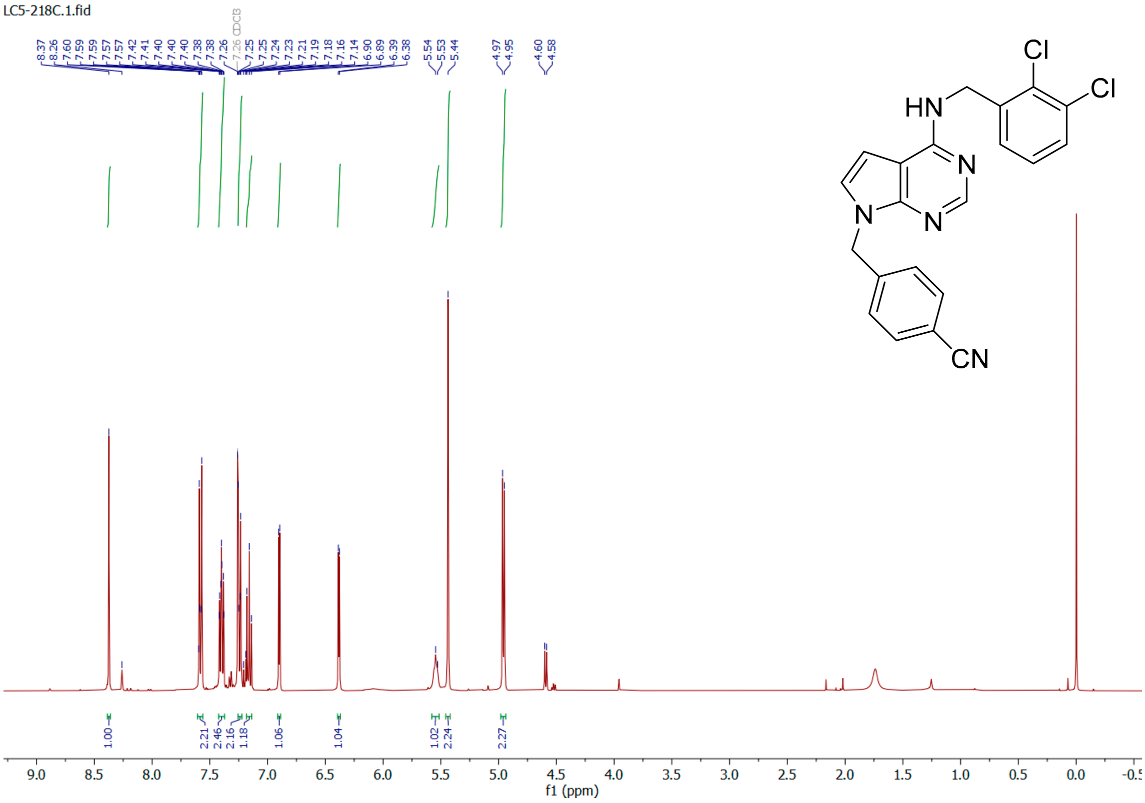

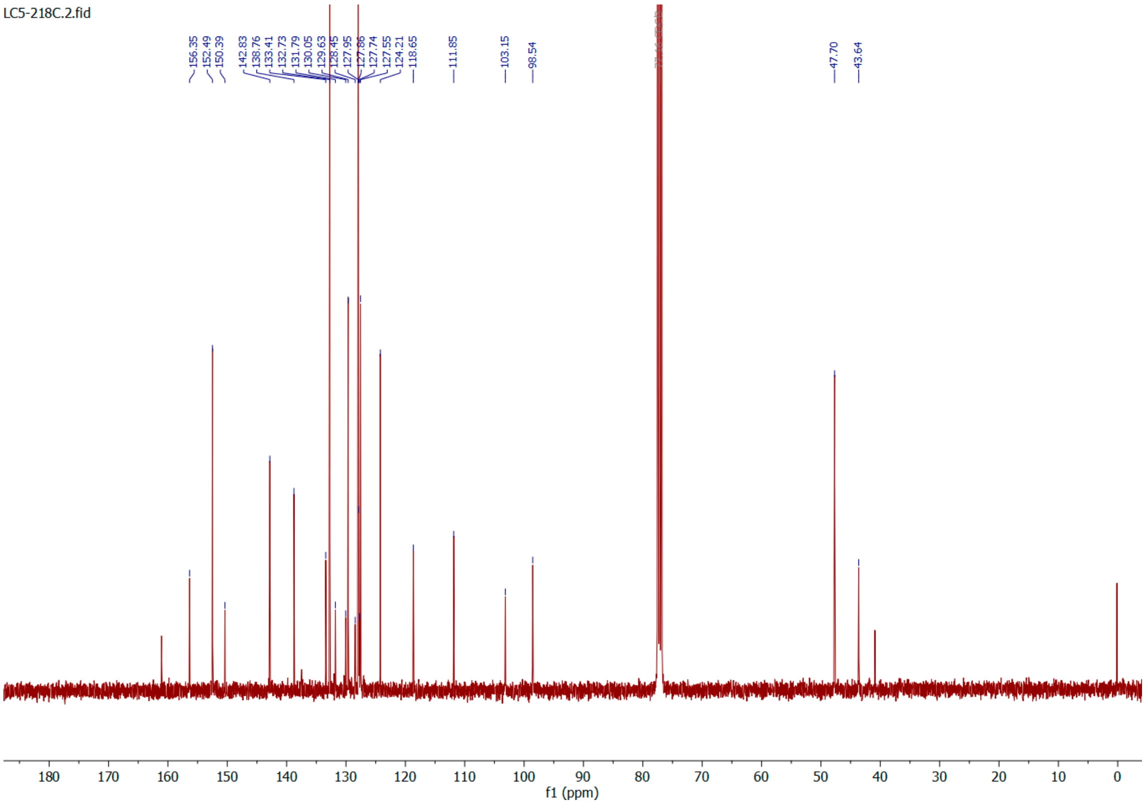

Figure S46. ^{1}H and ^{13}C spectra of compound **27**.

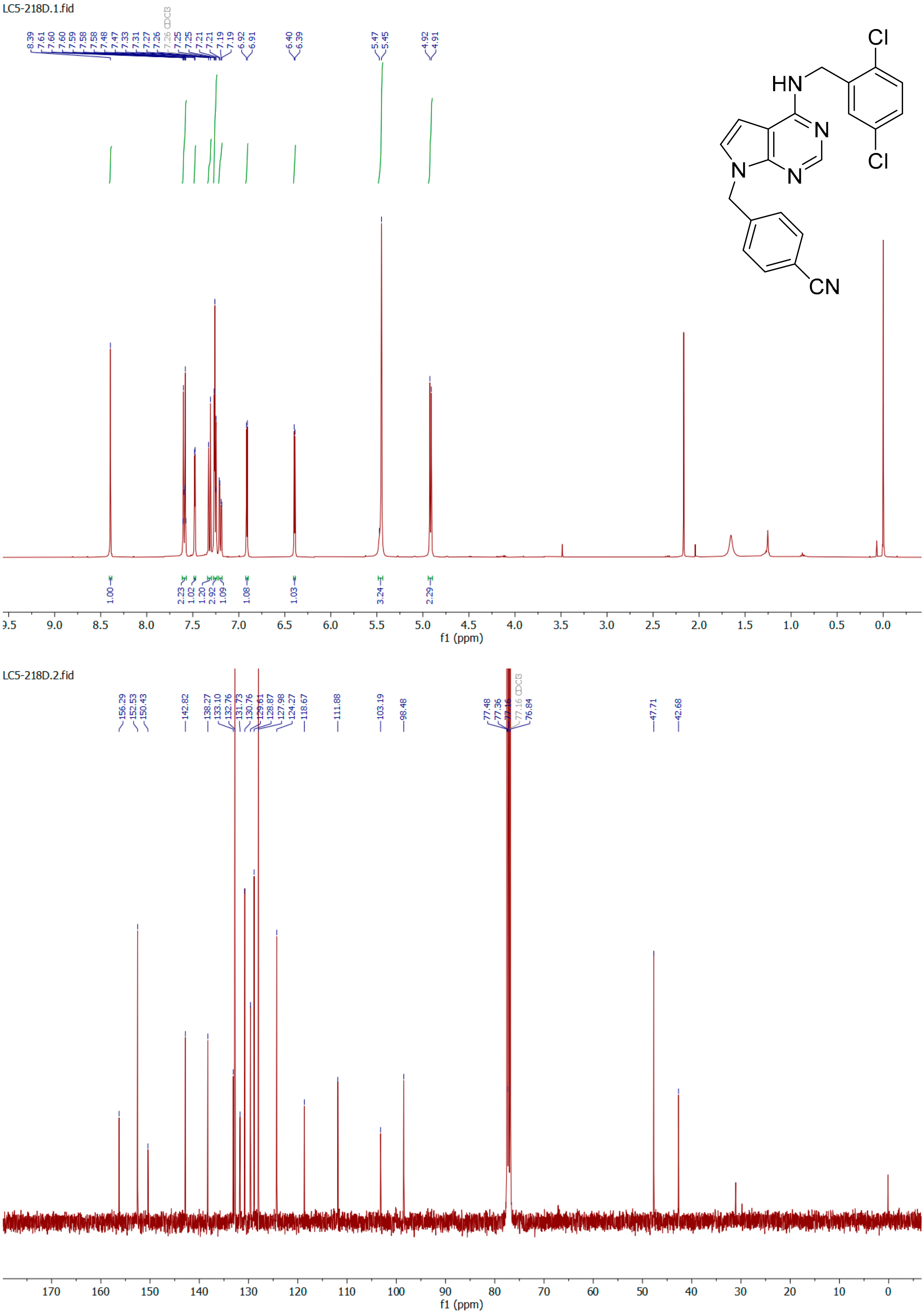

Figure S47. ^{1}H and ^{13}C spectra of compound **28**.

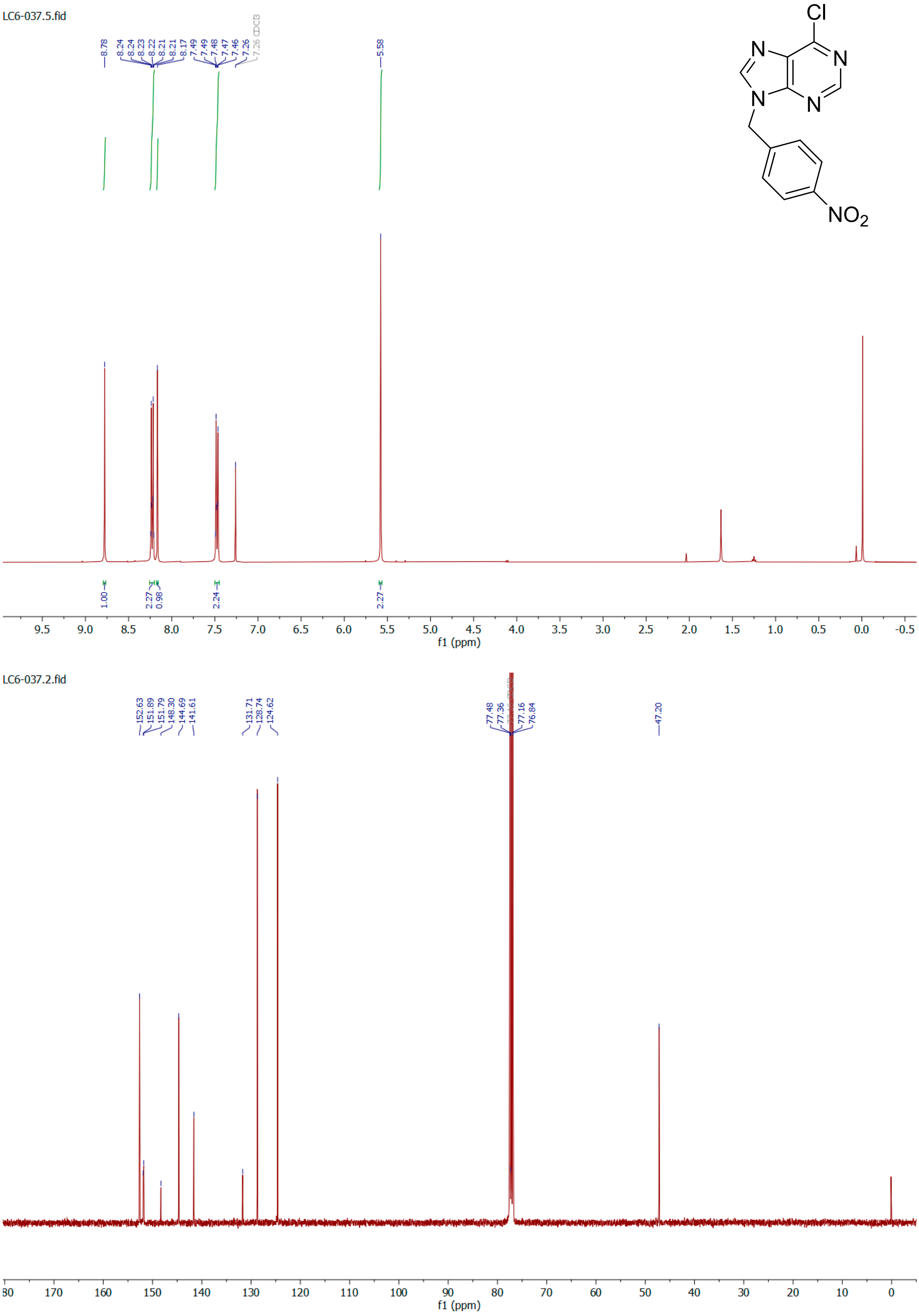

Figure S48. ^{1}H and ^{13}C spectra of compound **36a**.

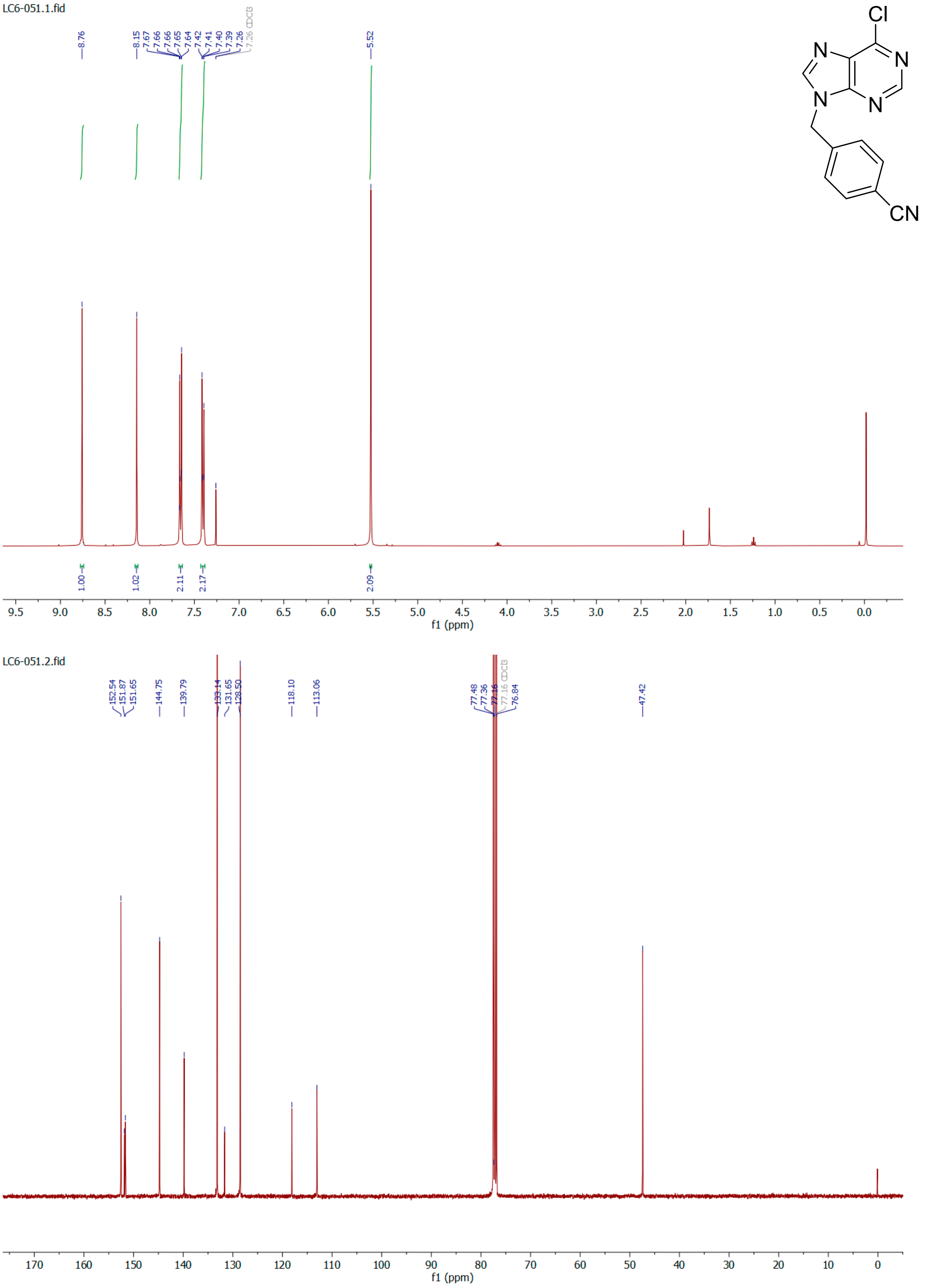

Figure S49. ^{1}H and ^{13}C spectra of compound **36b**.

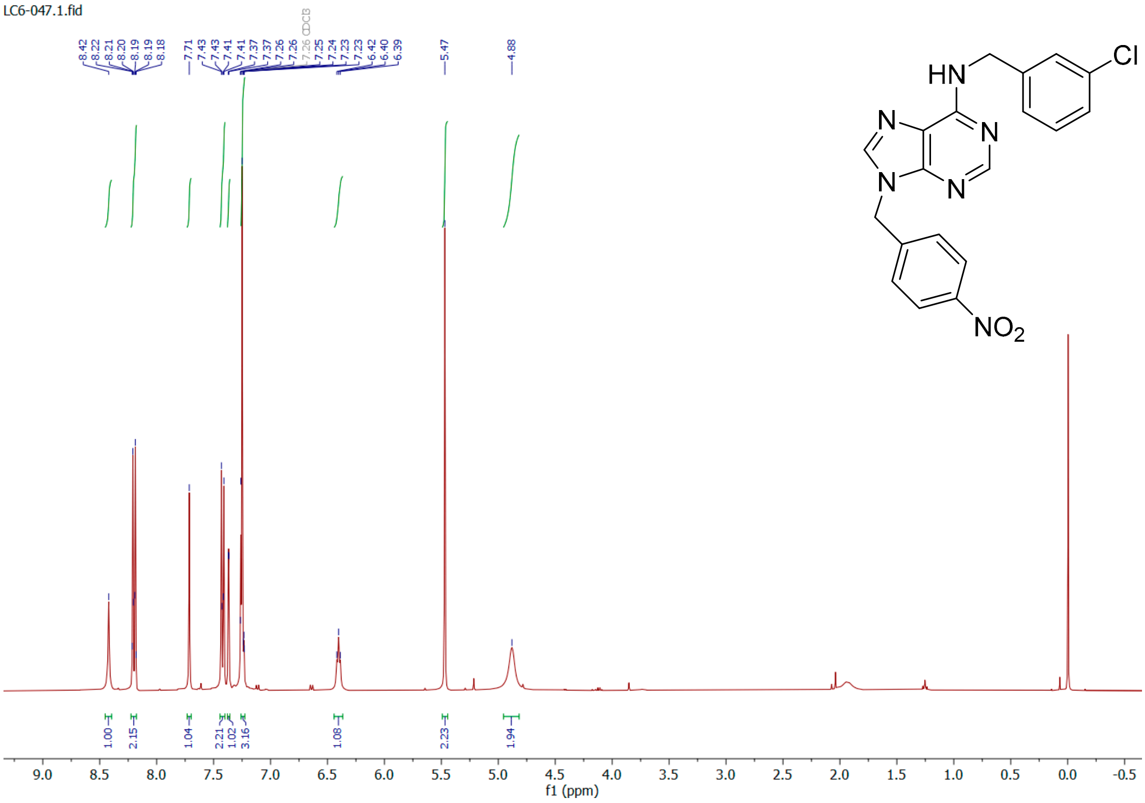

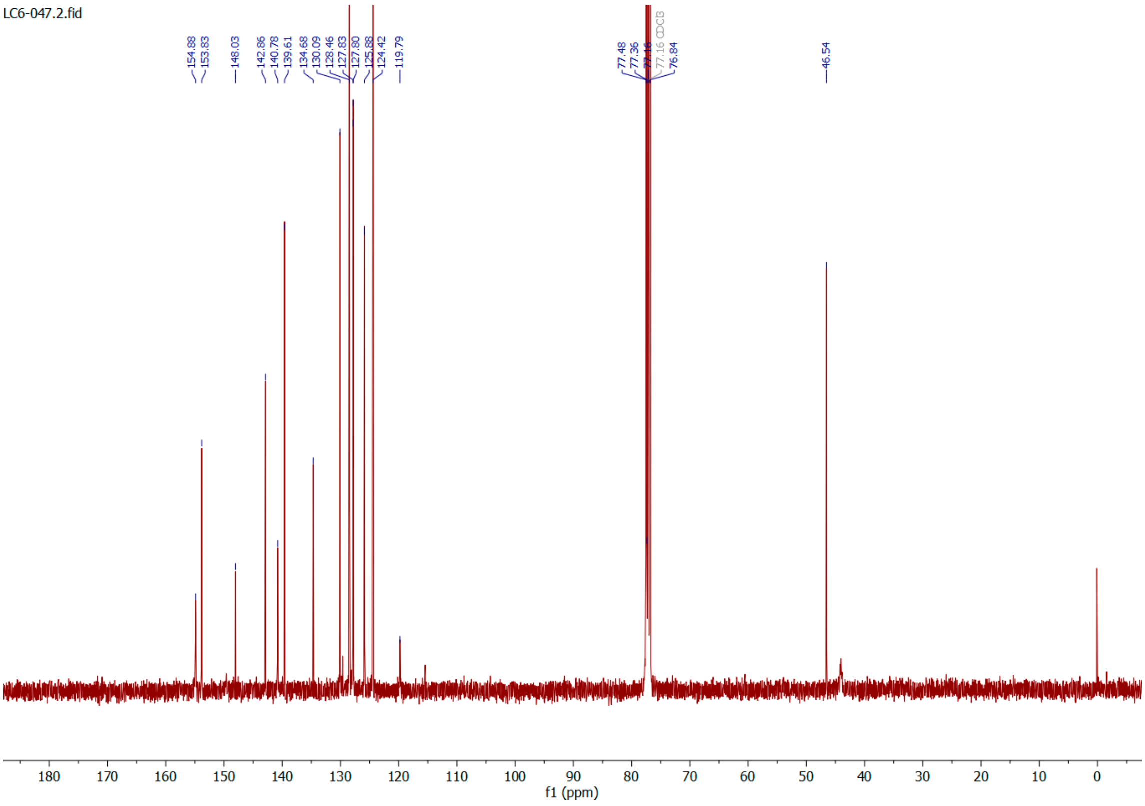

Figure S50. ^{1}H and ^{13}C spectra of compound **29**.

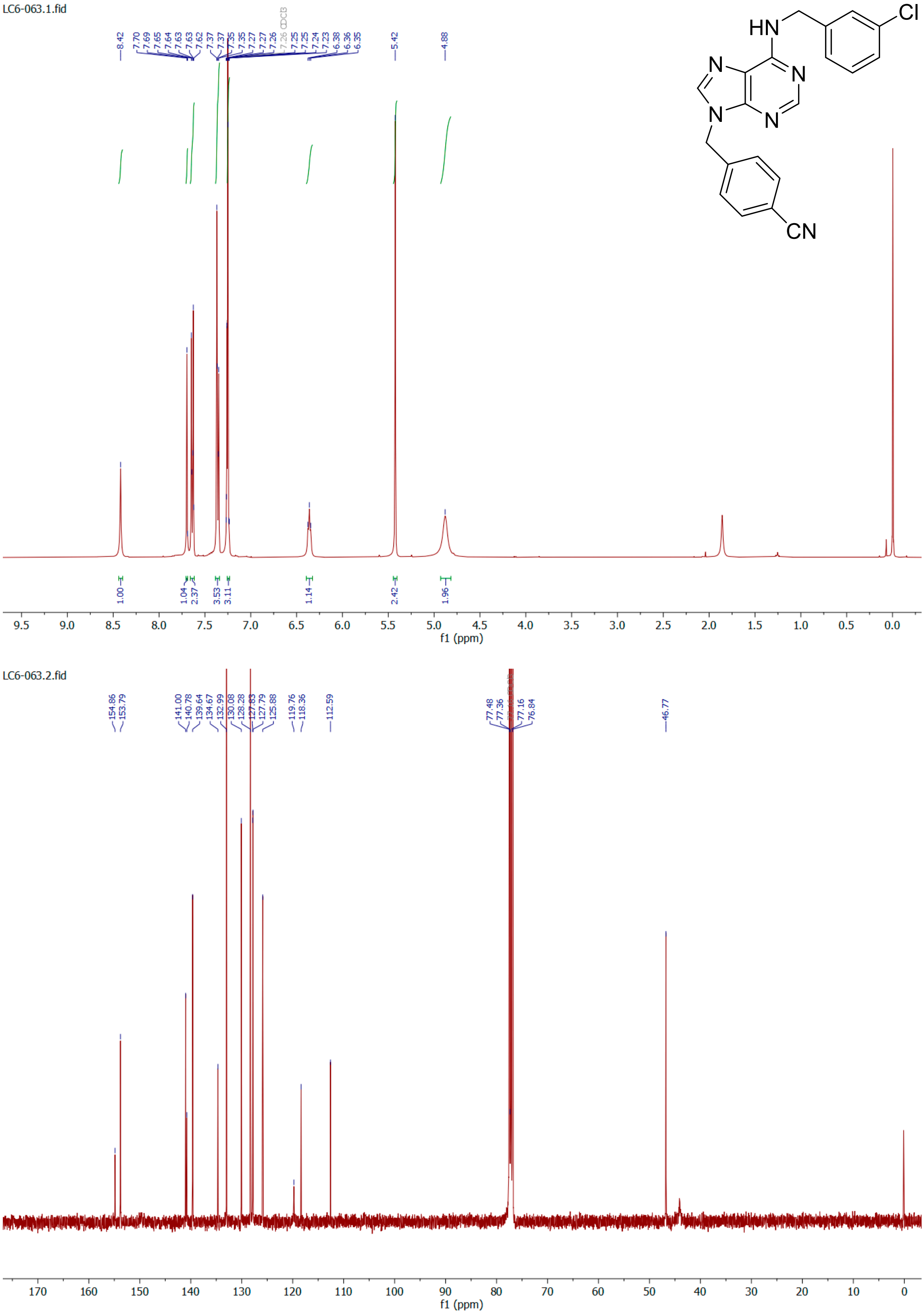

Figure S51. ^{1}H and ^{13}C spectra of compound **30**.

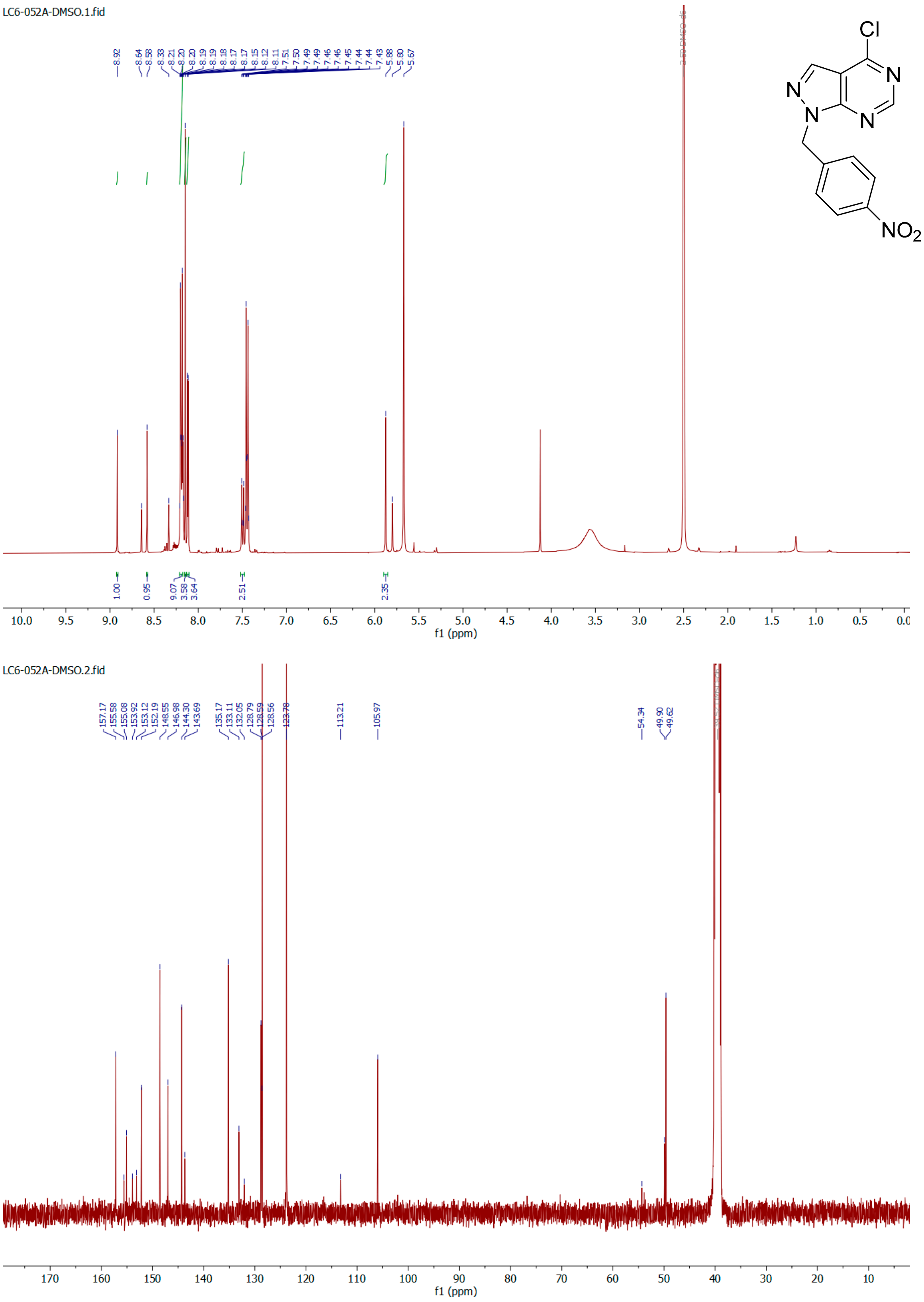

Figure S52. ^{1}H and ^{13}C spectra of compound **38a**.

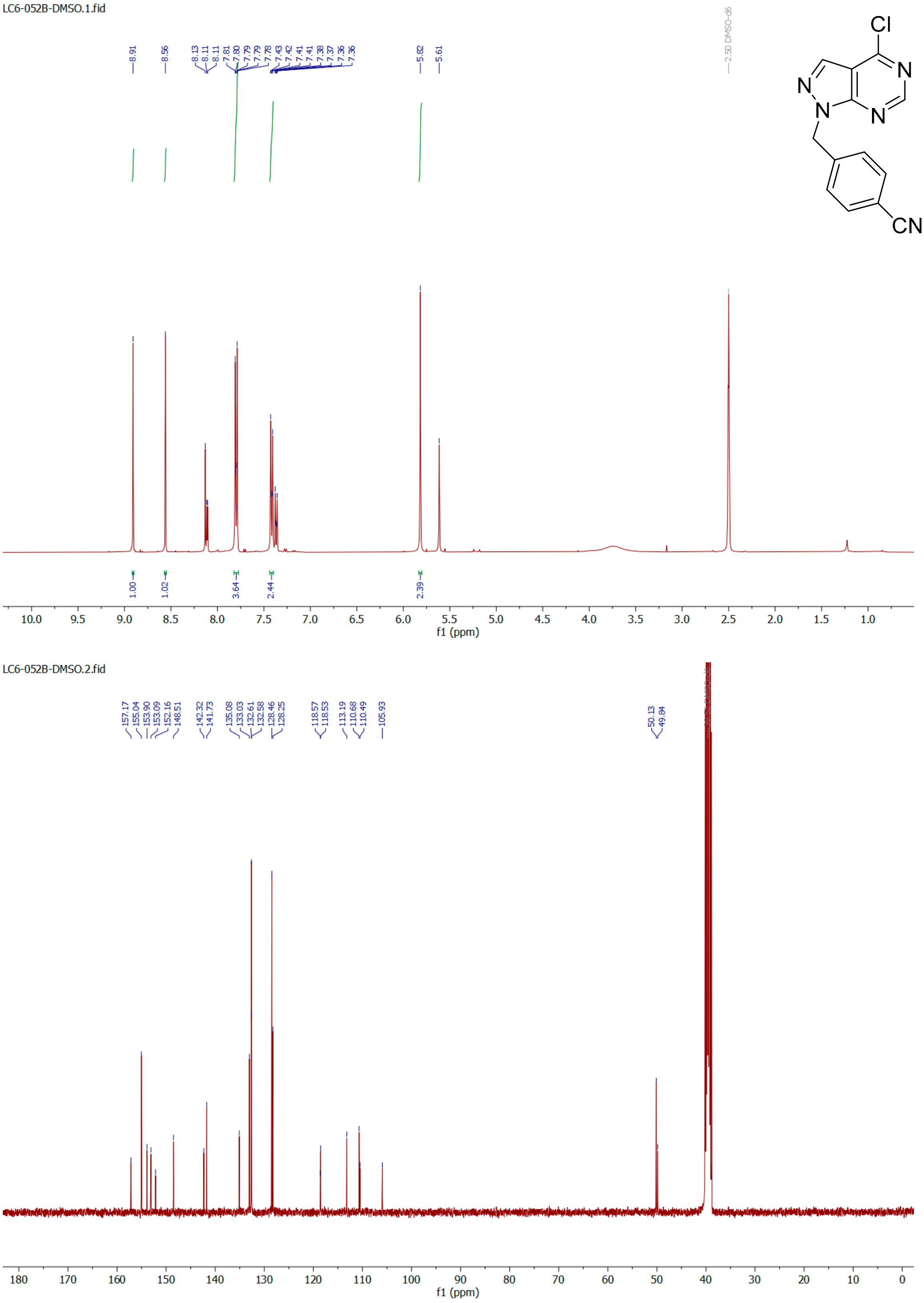

Figure S53. ^{1}H and ^{13}C spectra of compound **38b**.

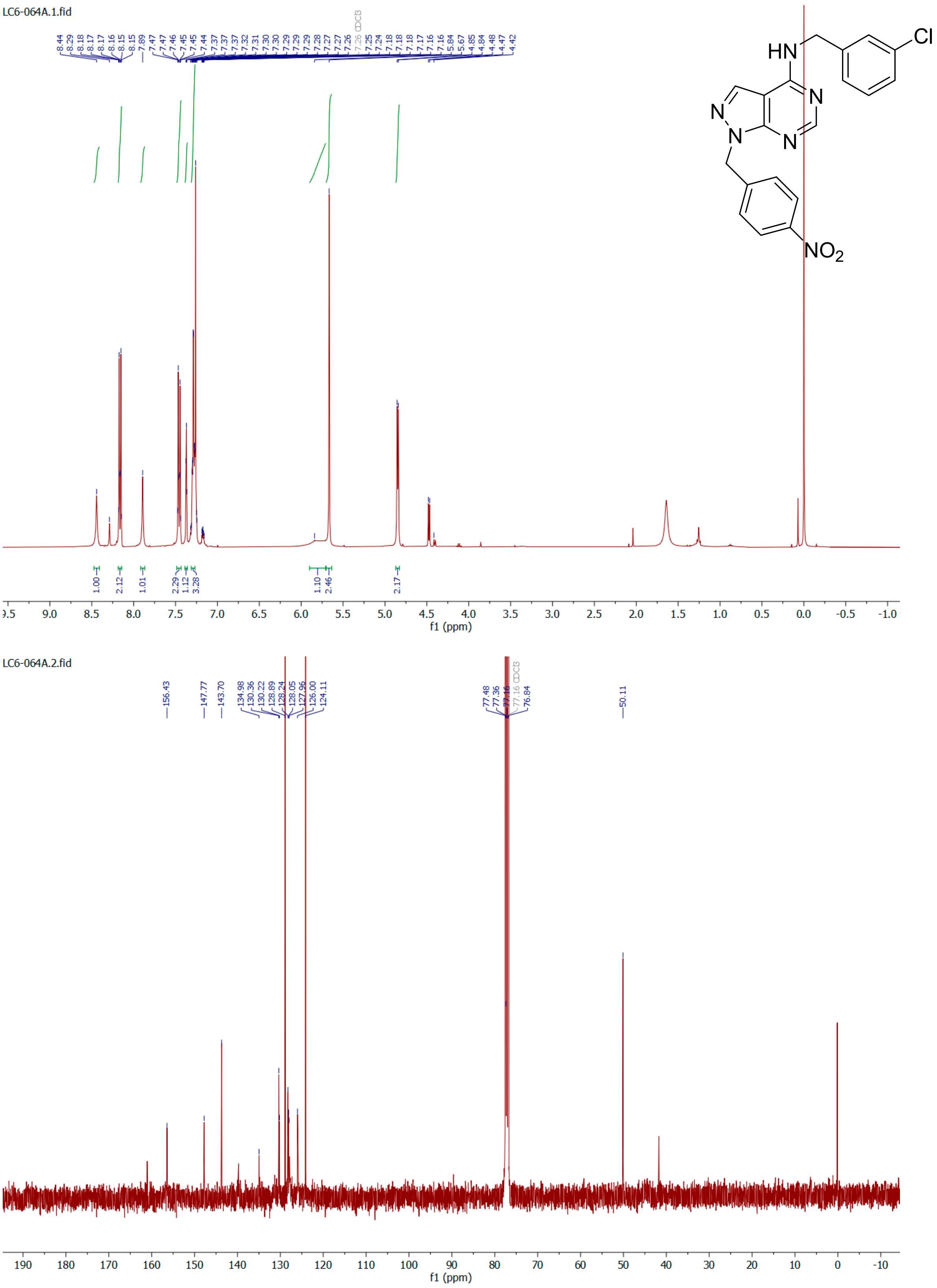

Figure S54. ^{1}H and ^{13}C spectra of compound **31**.

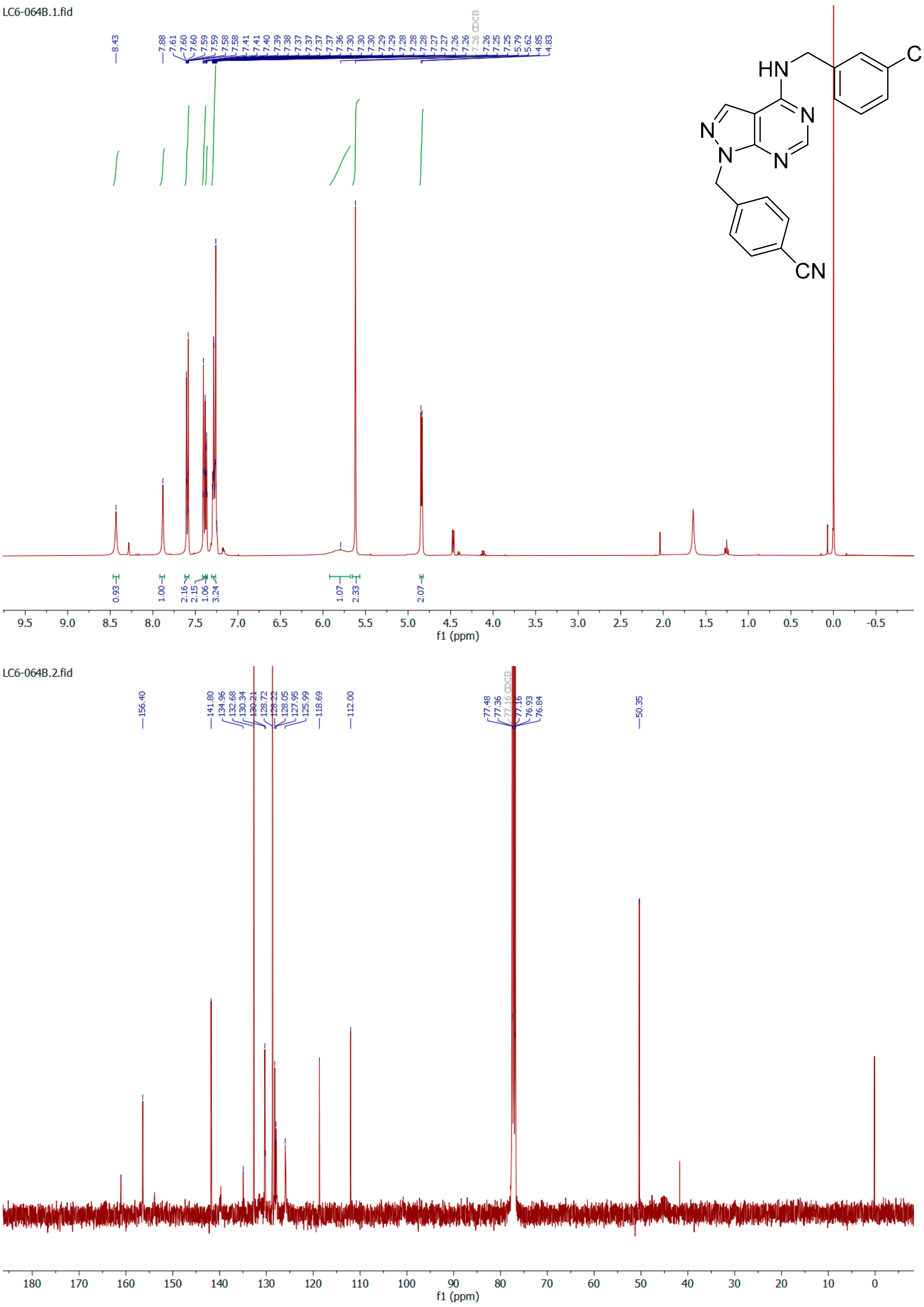

Figure S55. ^{1}H and ^{13}C spectra of compound **32**.

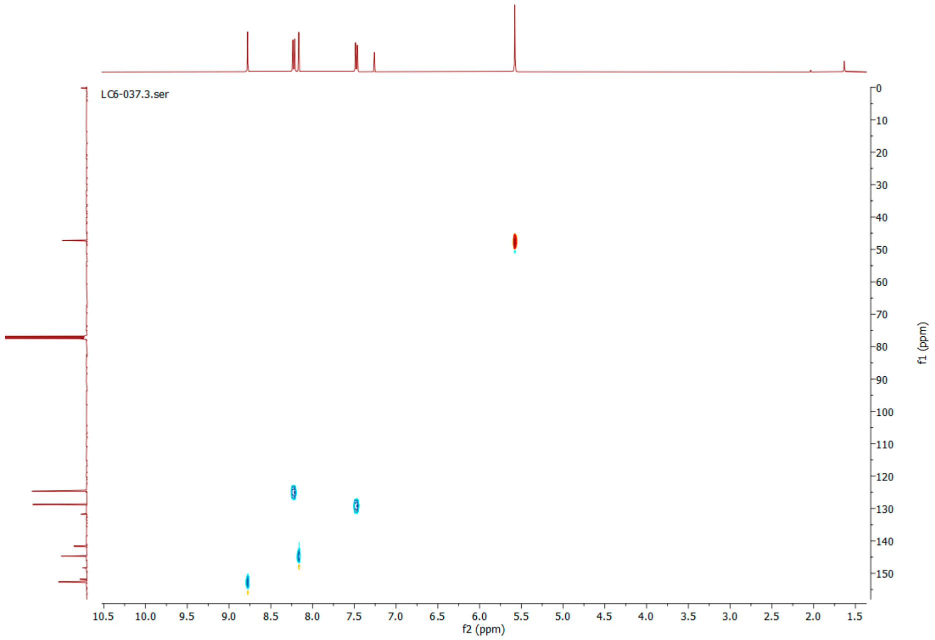

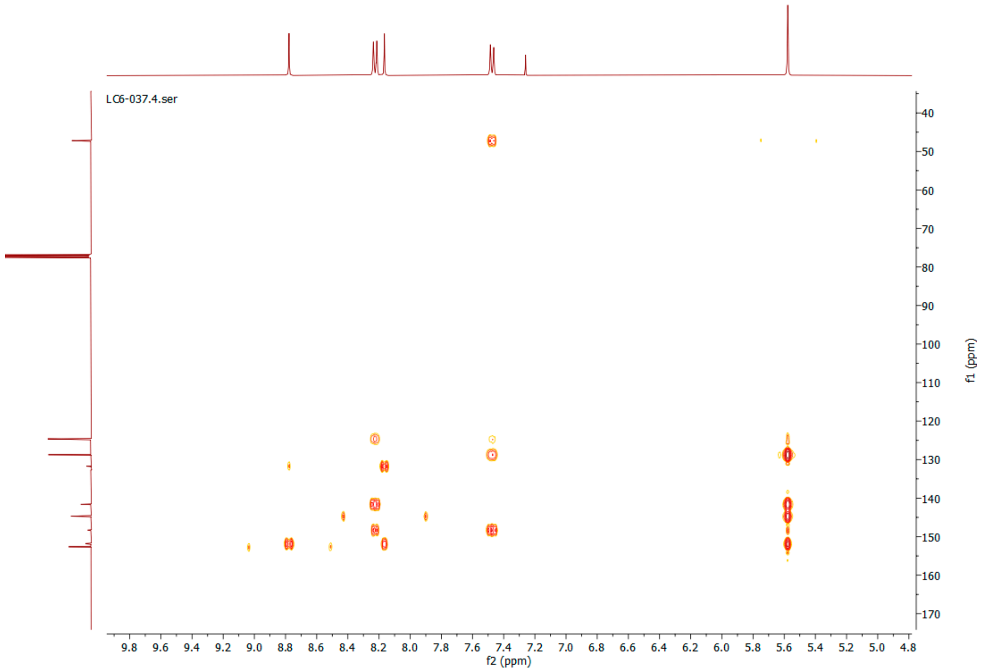

Figure S56. HSQC and HMBC spectra of chloride **36a**.

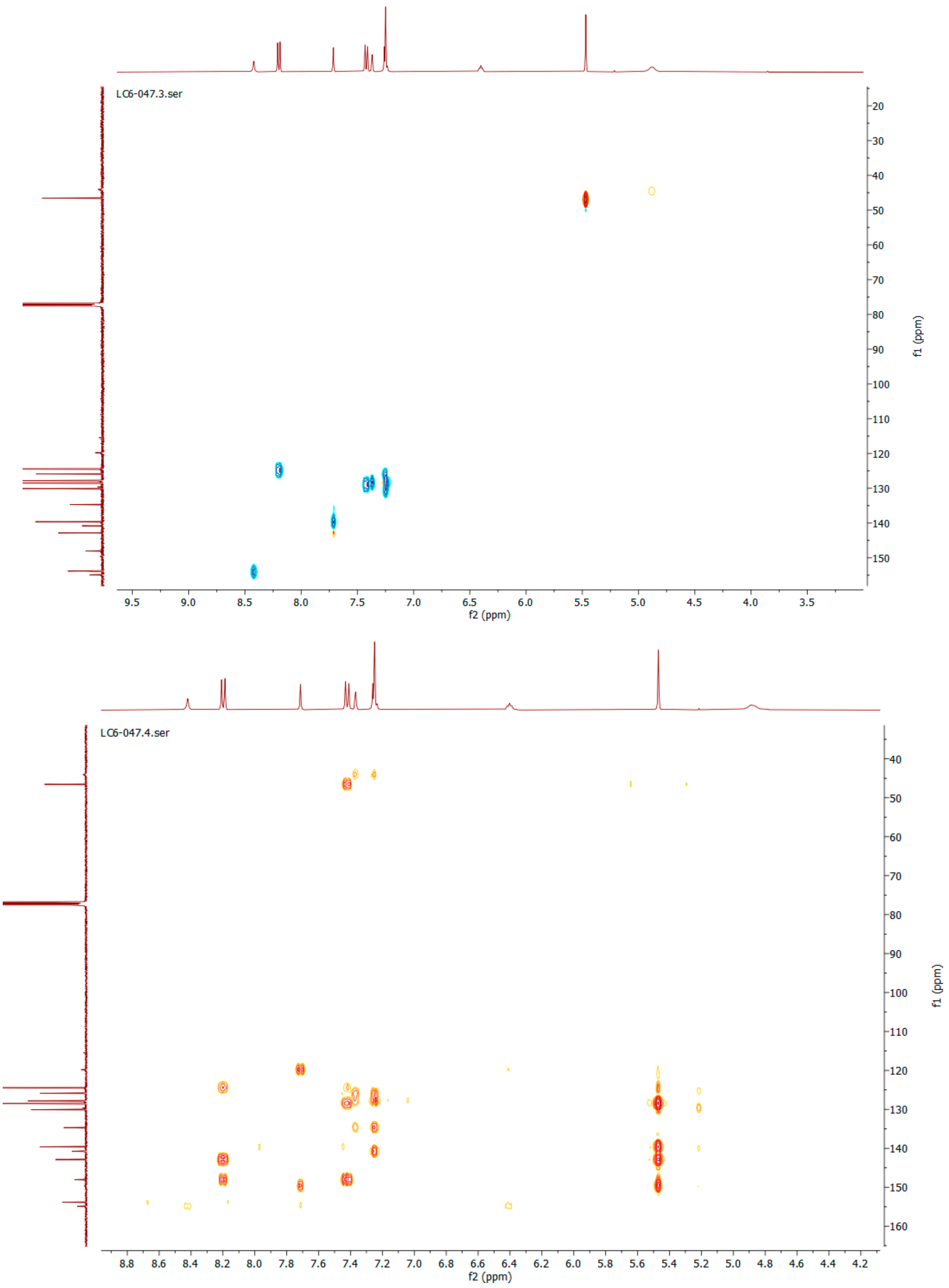

Figure S57. HSQC and HMBC spectra of compound **29**.
